# Unveiling p65 as the target of diphyllin in ameliorating metabolic dysfunction-associated steatotic liver disease via targeted protein degradation technology

**DOI:** 10.3389/fphar.2025.1567639

**Published:** 2025-04-28

**Authors:** Xuejing Zhu, Lei Zhang, Wenqian Cui, Liangjie Wang, Fengjing Xu, Mengyuan Liu, Shuangcheng Chen, Haowen Jiang, Zhiying He, Chang Peng, Jinlong Li

**Affiliations:** ^1^ Institute for Regenerative Medicine, State Key Laboratory of Cardiology and Medical Innovation Center, Shanghai East Hospital, School of Life Sciences and Technology, Tongji University, Shanghai, China; ^2^ Shanghai Engineering Research Center of Stem Cells Translational Medicine, Shanghai East Hospital, Shanghai, China; ^3^ School of Pharmacy, Nantong University, Nantong, Jiangsu, China; ^4^ Department of pharmacy, Nantong Health College of Jiangsu Province, Nantong, Jiangsu, China; ^5^ State Key Laboratory of Chemical Biology, Shanghai Institute of Materia Medica, Chinese Academy of Sciences, Shanghai, China; ^6^ Hangzhou Institute for Advanced Study, University of Chinese Academy of Sciences, Hangzhou, China

**Keywords:** metabolic dysfunction-associated steatotic liver disease, diphyllin, p65, targeted protein degradation technology, NRF2

## Abstract

**Introduction:**

Metabolic dysfunction-associated steatotic liver disease (MASLD), characterized by hepatic steatosis, inflammation and fibrosis, is becoming a global epidemic. However, the currently available effective clinical strategies remain limited.

**Methods:**

We conducted the choline-deficient, L-amino acid-defined, high-fat diet (CDAHFD) induced MASH mouse model to explore the effects of diphyllin on MASLD mice. We employ the targeted protein degradation technology applied for the discovery of compound/protein-protein interaction to identify p65 as a potential target protein.

**Results:**

We determine that diphyllin, a natural arylnaphthalene lignan lactone, is effective on MASLD, evidenced by the inhibition of hepatic lipid accumulation through promoting fatty acid oxidation in vivo and in vitro. To uncover the underlying mechanisms, we design and synthesis diphyllin-based protac and identify p65 as a potential target protein. Under p65 deficiency, the effects of diphyllin on lipid metabolism are blocked in vitro. As p65 as an antagonist of NRF2, diphyllin interacts with p65, leading to the induction of the NRF2 transcriptional activity and the enhancement of antioxidant capacity. When NFR2 is inhibited, the lowering effects of diphyllin on lipid is abolished.

**Discussion:**

Our study presents diphyllin as a potential lead compound for MASLD therapy but also offers a novel approach for elucidating the mechanisms of action of natural products.

## Introduction

Metabolic dysfunction-associated steatotic liver disease (MASLD) is a multifaceted pathological condition characterized by excessive hepatic fat accumulation, inflammation, and fibrosis, and it can ultimately progress to cirrhosis, hepatocellular carcinoma, and end-stage liver disease, leading to liver-related mortality and posing a significant threat to global public health (Michelotti et al., 2013; Pais et al., 2016; Tao et al., 2021). It is estimated that approximately 25% of patients with fatty liver, which is marked by excessive hepatic fat accumulation, will develop metabolic dysfunction-associated steatohepatitis (MASH) (Vieira Barbosa and Lai, 2021). The prevalence of MASH in adults is currently between 3% and 6%, with projections indicating that this figure may increase to approximately 56% within the next decade (Michelotti et al., 2013; Huang et al., 2021; Sheka et al., 2020). Despite decades of research into the pathogenesis and potential therapies for MASH, only one drug has recently been approved for its treatment (Kingwell, 2024). The development of effective therapeutic strategies remains both challenging and urgently needed.

The dysregulation of lipid metabolism and reactive oxygen species (ROS) balance in the liver is a critical factor in the progression from MASLD to MASH (Arroyave-Ospina et al., 2021). The accumulation of free fatty acids in hepatocytes under MASLD conditions enhances mitochondrial β-oxidation, leading to excessive ROS production as a byproduct of intensified lipid metabolism (Middleton and Vergis, 2021; Schofield and Schafer, 2021). Studies have shown that elevated ROS levels can impair mitochondrial function by inhibiting key respiratory chain enzymes, resulting in the release of mitochondrial DNA (mtDNA) and mitochondrial dysfunction (Shimada et al., 2012; Jun et al., 2020). Additionally, ROS can disrupt several protein kinases and nuclear transcription factor pathways, ultimately exacerbating hepatic inflammation and fibrosis (Ma et al., 2021). Furthermore, ROS promotes the secretion of pro-inflammatory cytokines such as IL-1β and TNF-α in Kupffer cells and stimulates the synthesis of extracellular matrix components such as collagen and α-SMA in stellate cells, collectively contributing to MASH development and liver injury (Cai et al., 2016; Liang et al., 2016; Wree et al., 2014; Zeng et al., 2016a; Zhao et al., 2019). Given the detrimental effects of ROS on antioxidant balance, inflammatory responses, and fibrosis, therapeutic strategies aimed at reducing ROS levels hold significant promise in the treatment of MASH.

Nuclear factor (erythroid-derived 2)-like 2 (NRF2) is a transcription factor that responds to oxidative stress and plays a crucial role in maintaining redox homeostasis as it activates the transcription of antioxidant response elements (AREs), thereby regulating proteins involved in combating oxidative stress (Solano-Urrusquieta et al., 2020). Under normal conditions, in the absence of ROS, NRF2 interacts with Kelch-like ECH-associated protein-1 (KEAP1) via its Neh2 domain and is degraded through the ubiquitin–proteasome pathway (Li and Kong, 2009). In inflammatory or oxidative stress conditions, elevated ROS levels promote the dissociation of NRF2 from KEAP1, allowing active NRF2 to translocate into the nucleus and induce the expression of antioxidant genes, thereby protecting cells from injury (Smolkova et al., 2020). Studies have shown that Nrf2-deficient mice exhibit exacerbated inflammation and steatosis in the liver when exposed to a methionine- and choline-deficient (MCD) diet, underscoring the critical protective role of NRF2 in liver injury (Chowdhry et al., 2010). Furthermore, research indicates that NRF2 deficiency contributes to the development of insulin resistance in the liver in the context of MASLD, while NRF2 activation enhances insulin sensitivity (Liu et al., 2016; Yu et al., 2019), highlighting its significant protective effect against liver diseases. Based on these findings, both natural and synthetic Nrf2 activators are being explored for the treatment of MASLD and MASH; however, due to their electrophilic nature, the potential side effects of Nrf2 activators cannot be overlooked (Arroyave-Ospina et al., 2021). Therefore, it remains imperative to identify novel Nrf2 activators for the effective treatment of MASLD.

Bioactive natural products are a crucial source of lead compounds for drug discovery, with target identification being key to understanding their mechanisms and advancing drug development (Newman and Cragg, 2020). Classical approaches often rely on phenotypic analysis or affinity-based strategies using cell lysates (Yoshida, 2019). Although these methods are informative, lysate-based strategies disrupt the native spatiotemporal dynamics of protein interactions and localization, potentially altering target conformation, function, and activity, thereby reducing the accuracy of target identification. To address these limitations, targeted protein degradation (TPD) strategies offer a more precise alternative (Chen et al., 2024; Liu et al., 2024; Ni et al., 2024; Wu et al., 2022). By inducing the degradation of natural product-interacting proteins within intact cells, TPD preserves the physiological context, capturing dynamic protein interactions and modifications. Notably, TPD does not rely solely on binding affinity, reducing false negatives caused by low protein abundance or weak interactions (Bekes et al., 2022). These features make TPD a promising tool for uncovering natural product targets, enhancing accuracy, and advancing the understanding of their biological activity.

Diphyllin, a natural arylnaphthalene lignan lactone, is characterized by multiple bioactivities, including antiviral, antitumor, anti-inflammatory, and antioxidant effects (Hou et al., 2023). In our study, we determine that diphyllin exhibits significant biological effects in improving MASLD. Then, we discover a TPD-based strategy, leveraging its dynamic spatiotemporal profiling capabilities, to identify potential molecular targets of diphyllin. This approach aims to deepen our understanding of diphyllin’s mechanism of action and further explore its therapeutic potential.

## Materials and methods

### Materials

Choline-deficient, L-amino acid-defined, high-fat diets (CDAHFD) were obtained from Research Diets (Cat# A06071302). The primary antibodies of p65 (Cat #3034) and GAPDH (Cat# 5174) were obtained from Cell Signaling Technology. Lipofectamine 3000 and Lipofectamine^®^ RNAiMAX Reagent were from Invitrogen. NRF2-IN-3 (HY-149508) was obtained from MedChemExpress. The luciferase activity detection kit (RG027) was from Beyotime. AML 12 cells were from ATCC and were cultured in DMEM supplemented with 10% FBS, 0.5% ITS, and 40 ng/mL dexamethasone.

### Animal study

This study was approved by the Chinese Academy of Sciences Committee on Animal Ethics for the Use of Laboratory Animals following the Animal Welfare Legislation of China. All male mice were housed under specific pathogen-free conditions and reared in line with standardized methods at 22 ± 1°C on a 12-h light/dark cycle with free access to food and water. C57BL/6J mice were fed either a chow diet (with 70% calories from starch) or a choline-deficient amino acid-defined high-fat diet (CDAHFD, Research Diets, Inc. A06071302) for 4 weeks. The CDAHFD mice were randomly divided into two subgroups to receive diphyllin orally or in its vehicle (2% castor oil + 1% DMSO + 0.5% methylcellulose; control group). At the end of the study, mice were fasted for 6 h, and then blood samples and liver tissue were collected. Plasma levels of lactate dehydrogenase (LDH), aspartate aminotransferase (AST), and alanine aminotransferase (ALT) were determined by an Olympus AU 600 auto-analyzer (Olympus, Japan).

### Pharmacokinetic studies

Pharmacokinetic analyses of oral diphyllin were performed in C57BL/6J mice. Briefly, 100 mg/kg diphyllin dissolved in 2% castor oil + 1% DMSO + 0.5% methylcellulose was given to male C57BL/6J mice, and blood samples and tissue were collected at 2.5-h treatment. All samples were subjected to liquid chromatography–tandem mass spectrometry analyses.

### Hepatic triacylglycerol, cholesterol, and hydroxyproline determination

The quantities of total triacylglycerol and total cholesterol were then assayed according to the protocols of the Triglyceride Content Determination Kit and the Cholesterol Content Determination Kit (Fosun Pharmaceutical, China). Triacylglycerol and cholesterol concentration were calculated as the concentration of standards and normalized to tissue weight for liver tissues. Hepatic collagen content was determined as relative hydroxyproline (μg/g liver) in 100-mg liver samples after hydrolysis in 6N HCl for 22 h at 120°C. Total hydroxyproline was calculated based on individual liver weights and the corresponding relative hydroxyproline content.

### Immunohistochemical staining

For histology, terminal mice liver tissue samples were collected from the left lateral lobe and fixed overnight in 4% paraformaldehyde. Liver tissue was paraffin-embedded and sectioned. Sections were stained with Sirius red and oil red to assess hepatic steatosis.

### Cellular triacylglycerol determination

AML 12 cells or L02 cells were seeded into a 12-well plate for 24 h and BSA-conjugated with 1.5 mM FFA (1.0 mM oleate and 0.5 mM palmitate) combined with compounds was added into cells for 24 h. For the quantification of cellular triglyceride levels, cells were cultured with 0.5 mL of PBS, and then total triglyceride was extracted with 1.5 mL of the solvent [chloroform and methanol, 2:1 (v/v)] overnight. The samples were centrifuged at 2,500 rpm for 10 min. The bottom liquid phase was transferred to a clean tube and air-dried. The remaining triglycerides were then dissolved in 1 mL of ethanol containing 1% Triton X-100. Final triglyceride contents were determined using the triglyceride determination kit and normalized to the total protein.

### Immunoblotting

Cells were prepared in RIPA buffer (50 mM Tris-HCl, pH 8.0, 150 mM NaCl, 1% NP-40, 1 mM Na_3_VO_4_, 1 mM dithiothreitol, 1 mM EDTA, and 1 mM EGTA) containing complete protease inhibitors (Roche, Basel, Switzerland). The protein was electrophoresed through SDS-PAGE after boiling for 10 min in SDS loading buffer. Prior to incubation with primary antibodies, membranes were blocked with 5% milk in TBS supplemented with 0.1% (v/v) Tween-20 for 1 h at room temperature. Chemiluminescent detection was completed with enhanced chemiluminescent (ECL) Western blotting reagents (GE Healthcare, RPN2236). Quantification was determined by measuring band intensities using ImageJ software.

### Cellular oxygen consumption rate

AML 12 cells were plated in Seahorse XF96 wells at 10,000 cells per well for 24 h. Seahorse XF96 Extracellular Flux Analyzer with XF96 FluxPaks (Seahorse Bioscience, United States) was used to determine the Seahorse-based oxygen consumption rate (OCR). The concentrations of A, B, and C in XF96 were 1 μM oligomycin, 1 μM FCCP, and 1 μM rotenone, respectively, combined with 1 μM antimycin.

### TMT-based quantitative proteomics analysis

We co-incubated DMSO (NC group) and 20 μM compound D-P (diphyllin–PROTAC group) with L02 cells for 24 h, followed by total protein extraction. Differential proteins between the NC and D-P groups (n = 4) were analyzed using TMT-based quantitative proteomics. The proteomics workflow, including protein extraction, TMT labeling, SCX fractionation, LC–MS/MS acquisition, and data processing, was conducted by Hoogen Biotech Co., Ltd. The outsourced work adhered to standardized protocols to ensure reliable and reproducible results. Proteins from L02 cells were extracted using SDT lysis buffer (4% SDS, 100 mM Tris/HCl, pH 7.6, and 0.1 M DTT) and quantified with the BCA assay. Proteins were digested with trypsin using the FASP method, desalted with a C18 cartridge, freeze-dried, and reconstituted in 40 μL dissolution buffer (Wisniewski et al., 2009). The peptide concentration was measured at OD_280_. Peptides (100 μg per sample) were labeled with TMT reagents following the manufacturer’s protocol (Thermo Fisher Scientific).

Labeled peptides were fractionated using an AKTA Purifier 100 system (buffer A was 10 mM KH_2_PO_4_, 25% ACN, pH 3.0; buffer B was 10 mM KH_2_PO_4_, 500 mM KCl, 25% ACN, pH 3.0). Separation was achieved at 1 mL/min with a gradient from 0% to 100% buffer B over 60 min. Fractions were collected every minute, freeze-dried, and desalted.

Each SCX fraction was analyzed using an Easy-nLC system (buffer A was 0.1% formic acid in water, and buffer B was 0.1% formic acid in 84% acetonitrile, Thermo Fisher Scientific) at a nano-flow rate. The separation system consisted of a trapping column (Thermo Scientific EASY Column, 100 μm × 2 cm, 5 μm, C18) and an analytical column (Thermo Scientific EASY Column, 75 μm × 10 cm, 3 μm, C18) operating at a flow rate of 250 nL/min. The gradient was 0%–35% buffer B over 50 min, 35%–100% over 8 min, and held at 100% for 2 min. Eluted peptides were analyzed using a Q-Exactive mass spectrometer (Thermo Fisher Scientific) in the positive-ion mode over a 60-min acquisition period. The full-scan range was 300–1800 m/z with a resolution of 70,000 at m/z 200, an AGC target of 3 × 10^6^, and a maximum injection time of 10 ms. Dynamic exclusion was set to 40.0 s. For MS/MS, the top 10 precursor ions were fragmented using HCD with a normalized collision energy of 30 eV. The isolation window was 2 m/z, and MS/MS spectra were acquired at a resolution of 17,500 at m/z 200, with an underfill ratio of 0.1%. MS data were analyzed with Proteome Discoverer 1.4, and proteins were identified and quantified based on database searches.

### Cellular thermal shift assay

L02 cells were cultured in four dishes until 80% confluency, with two dishes treated with 40 µM diphyllin for 3 h (experimental group) and two untreated dishes serving as controls. Cells were harvested using trypsin digestion, centrifuged at 1,000 rpm for 5 min, and washed two to three times with PBS. The washed cells were re-suspended in 1 mL of PBS containing PMSF, and 100 µL of the suspension was aliquoted into 10 labeled Eppendorf tubes per group, corresponding to the designated temperature conditions (control, 37°C, 40°C, 43°C, 46°C, 49°C, 52°C, and 55°C). The tubes were incubated in a metal bath set to their respective temperatures for 3 min, followed by immediate freezing in liquid nitrogen for 30 s and thawing at room temperature. This freeze–thaw process was repeated three times to generate cell lysates, which were then centrifuged at 15,000 rpm for 20 min at 4°C. The supernatant was collected for further analysis, while the pellet was discarded.

### Luciferase activity determination

AML 12 cells were transfected with pGL3–4 × ARE luciferase plasmids for 24 h in a 10-cm dish. Then, AML 12 cells were seeded in a 96-well plate and treated with compounds for 24 h. The culture medium was removed, and the luciferase activity detection kit was added into cells for examination.

### RNA interference and quantitative real-time PCR

AML-12 cells were seeded in a 24-well plate or a 12-well plate and transfected with synthesized siRNA by Lipofectamine^®^ RNAiMAX Reagent (13778150, Thermo Fisher Scientific, United States), following the manufacturer’s instructions, for follow-up experiments. The siRNA sequence for p65/NC is as follows: 5-CCA​UGG​AGU​UCC​AGU​ACU​UTT-3; and the si NC sequence is as follows: 5-GGA​GGT​GGT​TGA​CTT​TCA​TTT-3. Total RNA from cells or mouse livers was isolated using the TRIzol method (Invitrogen, Shanghai, China), and the results were analyzed on an ABI StepOne Plus real-time PCR system (Applied Biosystems, United States) using the 2^−ΔΔCT^ method. Beta-actin was used as an internal control, and relative mRNA levels were normalized to actin. The primers are listed in Supplementary Table S1.

### Statistical analysis

All results in this study are presented as mean ± SEM, and data were analyzed with two-tailed unpaired Student’s t-test between two groups or one-way ANOVA among multiple groups using GraphPad Prism 8 software. P < 0.05 was considered statistically significant.

## Results

### Diphyllin has a higher distribution in the liver

To determine the effects of diphyllin on the development of MASLD, we first examined the tissue distribution of diphyllin in mice. We examined its distribution in C57BL/6J mice at 2.5 h after oral injection with a dose of 100 mg/kg diphyllin. The results exhibited that diphyllin had a higher tissue distribution in the liver than in other organs (Supplementary Figure S1). Thus, the result of the tissue distribution of diphyllin indicated that diphyllin might exert its function in the liver.

### Diphyllin treatment improves the liver injury and lipid accumulation in the liver of the CDAHFD-induced MASH mouse model

**FIGURE 1:**
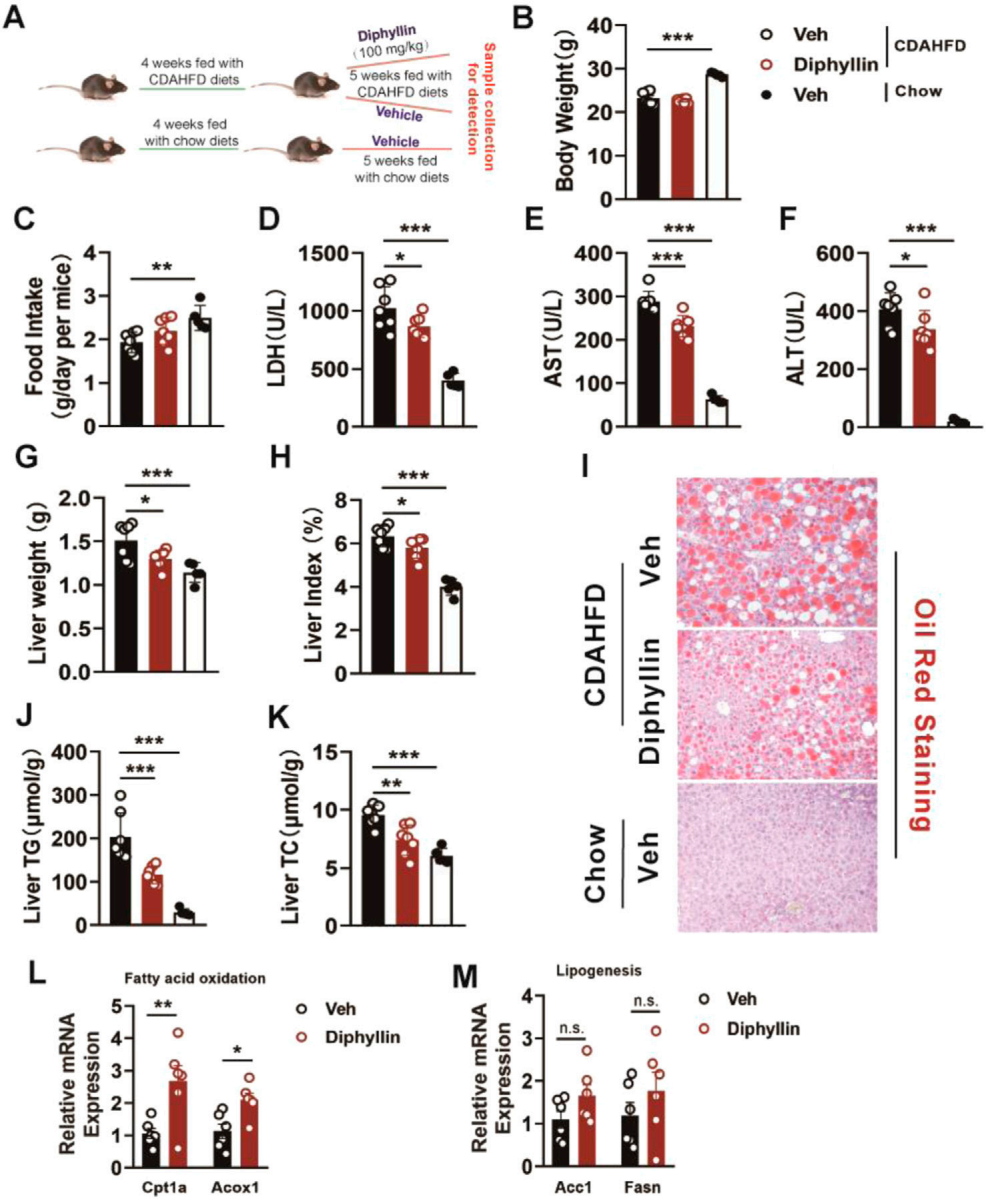
Diphyllin treatment ameliorates liver injury and hepatic steatosis in CDAHFD-induced MASH mice. **(A)** Schematic of mice fed with CDAHFD for 4 weeks and treated with 100 mg/kg diphyllin for 5 weeks together with CDAHFD. **(B)** Body weight. **(C)** Food intake. **(D–F)** Levels of plasma LDH, AST, and ALT from mice treated with diphyllin. **(G, H)** Liver weight and liver index. **(I)** Oil-red staining of liver of mice treated with diphyllin (scale bars, 100 μm). **(J, K)** Content of liver triglycerides and cholesterol in mice treated with diphyllin. **(L, M)** Gene levels in the liver from mice treated with diphyllin. For all, n = 6–7 mice for all mice groups. Bar graphs are presented as mean ± SEM, and one-way ANOVA was used for all comparisons.

To explore the effects of diphyllin on MASLD mice, we prepared the choline-deficient, L-amino acid-defined, high-fat diet (CDAHFD)-induced MASH mouse model (Yang et al., 2024). Next, we examined the effects of diphyllin on the CDAHFD-induced MASH mouse model (Figure 1A). The body weight and food intake of mice were unchanged after 5 weeks of treatment with 100 mg/kg diphyllin (Figures 1B, C). However, the liver injury caused by CDAHFD was alleviated by diphyllin treatment, which was evidenced by the decrease in the levels of plasma lactate dehydrogenase (LDH), aspartate aminotransferase (AST), and alanine aminotransferase (ALT) (Figures 1D–F). Meanwhile, an improvement in the liver weight was observed by diphyllin treatment (Figures 1G, H). Oil-red staining showed that the ballooning degeneration in the liver tissue significantly decreased after diphyllin treatment. Consistently, hepatic steatosis was alleviated by diphyllin treatment, which was evidenced by oil-red staining of liver tissue and hepatic triglyceride (TG) and cholesterol (TC) content detection (Figures 1I–K). Moreover, we detected the expression of the hepatic lipid metabolic genes and found that diphyllin treatment induced the expression of fatty acid oxidative genes, such as Cpt1α and Acox1, and did not affect the expression of lipogenic genes, such as Acc1 and Fasn (Figures 1L, M). Therefore, these data suggested that diphyllin exhibited protective effects on MASLD progression.

### Diphyllin inhibits the occurrence of inflammation and fibrosis in MASH mice

**FIGURE 2:**
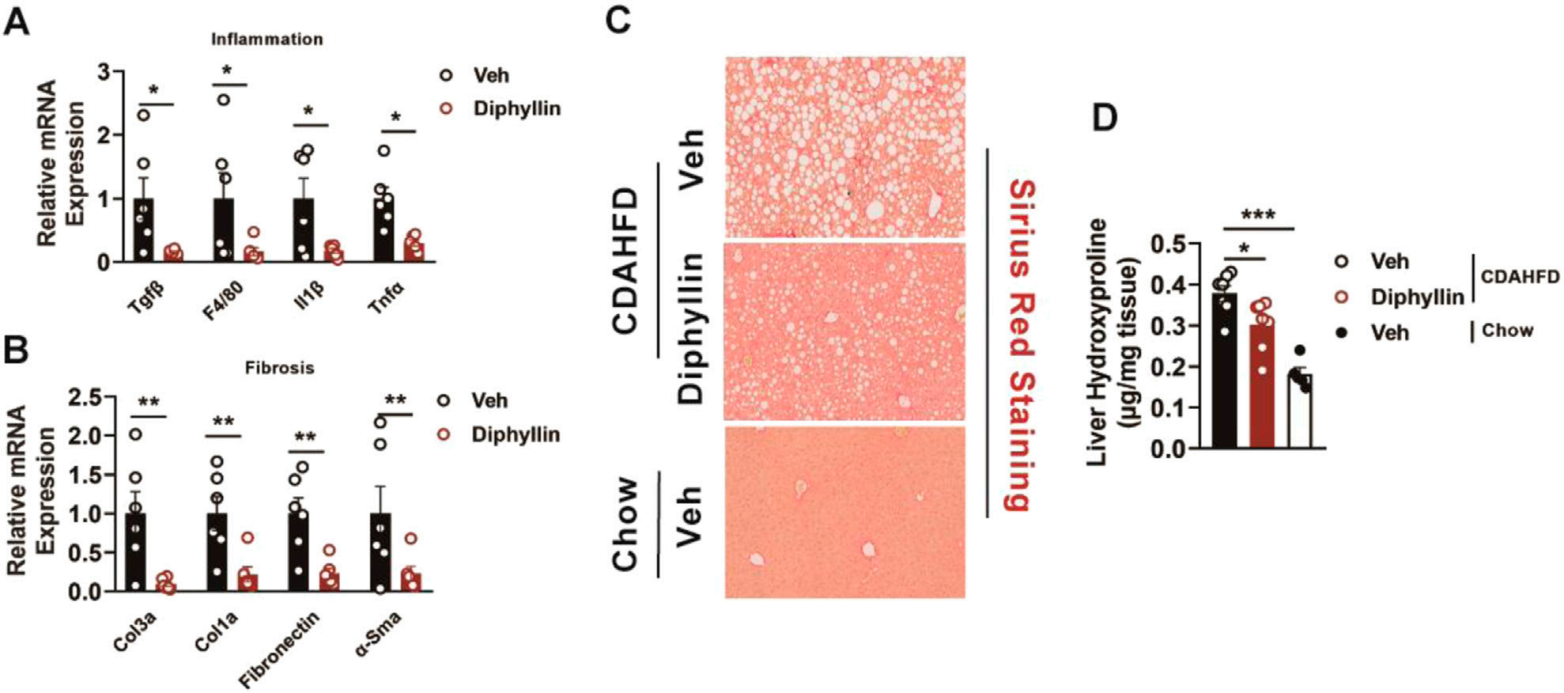
Diphyllin treatment improves inflammation and fibrosis in CDAHFD-induced MASH mice. **(A, B)** Inflammation- and fibrosis-related gene levels in the liver from mice treated with diphyllin. **(C)** Sirius red staining of liver of mice treated with diphyllin (scale bars, 100 μm). **(D)** Content of liver hydroxyproline in mice treated with diphyllin. For all, n = 6–7 mice for all mice groups. Bar graphs are presented as mean ± SEM, and one-way ANOVA was used for all comparisons.

Liver fibrosis and inflammation are the main pathological features during the progression of MASLD, which is regarded as MASH. Thus, we detected the expression of the inflammation and fibrotic genes of the liver from mice treated with diphyllin. The results showed that diphyllin treatment reduced the expressions of inflammatory genes such as Tgfβ, F4/80, Il1b, and Tnfα (Figure 2A). Meanwhile, the fibrotic genes, including Col3a, Col1a, fibronectin, and α-Sma, were also downregulated by diphyllin treatment (Figure 2B). Sirius red staining showed that fibrosis was decreased by diphyllin treatment (Figure 2C). Hydroxyproline is regarded as a crucial indicator of liver fibrosis (Steffen et al., 2022). We then examined the content of hepatic hydroxyproline and found that it was diminished in mice treated with diphyllin, indicating the remarkable improvement effects of diphyllin on fibrosis (Figure 2D). Thus, these results indicated that diphyllin could rescue the inflammation and fibrosis of MASH.

### Diphyllin reduces lipid accumulation via the promotion of fatty acid oxidation *in vitro*

**FIGURE 3:**
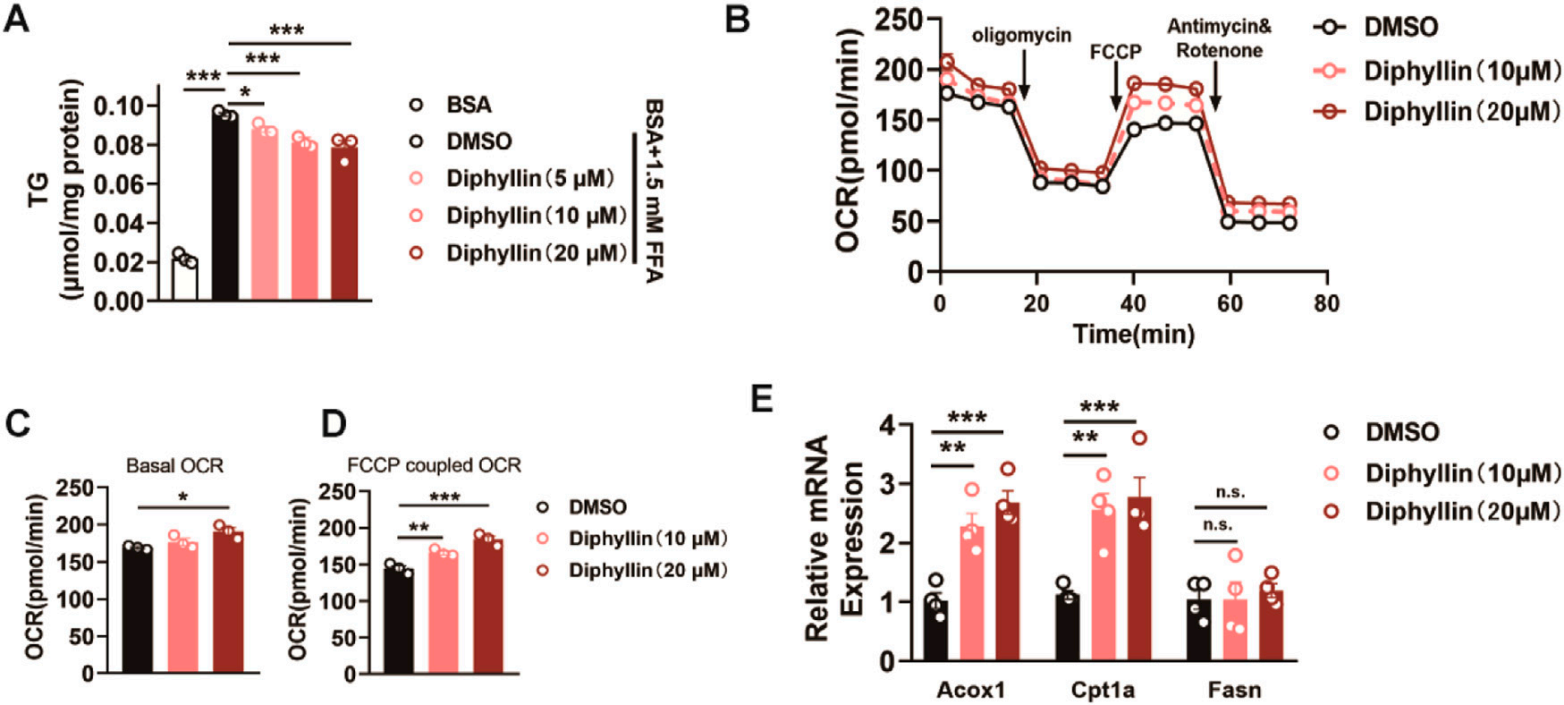
Diphyllin inhibits lipid accumulation via the promotion of fatty acid oxidation. **(A)** Content of triglyceride of AML 12 cells treated with 1.5 mM FFA and diphyllin for 24 h. **(B)** Curve of the oxygen consumption rate of AML 12 cells treated with diphyllin for 24 h. **(C)** Basal oxygen consumption rate. **(D)** Oxygen consumption rate of AML 12 cells treated with FCCP. **(E)** Gene levels in AML 12 cells treated with diphyllin for 24 h. For all, n = 3–4 for all groups. Bar graphs are presented as mean ± SEM, and one-way ANOVA was used for all comparisons.

Based on the protective effects of diphyllin against MASLD and its distribution in the liver, we further investigated the underlying mechanisms of diphyllin for the effects on MASLD. Hepatic steatosis, characterized by lipid accumulation in hepatocytes, is the primary stage of MASLD. Then, we examined the effects of diphyllin on lipid accumulation *in vitro*. We treated AML 12 cells with free fatty acids (palmitate and oleate), resulting in intracellular lipid accumulation. Following 24 h of treatment with diphyllin, the intracellular lipid content was diminished in a dose-dependent manner, indicating that the protective effects of diphyllin on MASLD might have resulted in the regulation of lipid metabolism (Figure 3A). According to the effects of diphyllin on enhancing hepatic fatty acid oxidation gene expression, we detected its role in fatty acid oxidation. We detected the oxygen consumption rate (OCR) in AML 12 cells treated with diphyllin for 24 h. The results showed that both basal OCR and FCCP-induced maximal OCR were dramatically induced by diphyllin, indicating that diphyllin could induce oxidative metabolism (Figures 3B–D). Consistently, we examined the fatty acid oxidative gene expression and found that Cpt1α and Acox1 were induced by diphyllin but not lipogenic genes, such as Fasn (Figure 3E). Thus, these results suggested that diphyllin reduced lipid metabolism via the promotion of fatty acid oxidation.

### Diphyllin regulates lipid metabolism depending on p65

Given the effects of diphyllin on lipid metabolism, we determined to identify the molecular targets of diphyllin; we employed a targeted protein degradation (TPD) strategy. A bioactive diphyllin-based proteolysis-targeting chimera (diphyllin–PROTAC, D-P) was designed (Supplementary Figure S2), incorporating diphyllin as the ligand for the target protein and pomalidomide as the ligand for the E3 ubiquitin ligase. Upon incubation with human liver L02 cells, the D-P molecule facilitates proximity-induced degradation of diphyllin-interacting proteins by recruiting them to the E3 ubiquitin ligase through a linker.

**FIGURE 4:**
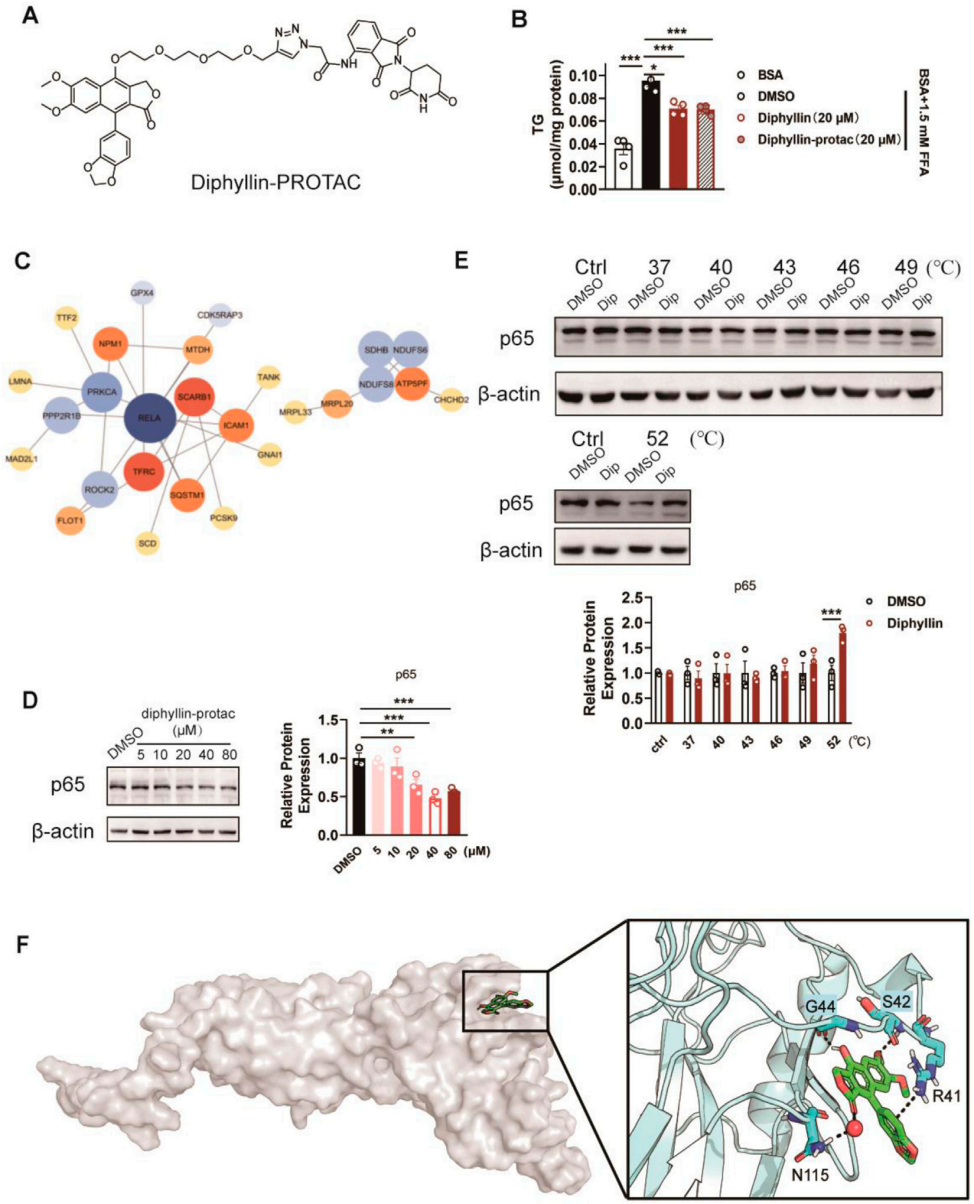
Identification of the target protein of diphyllin by the TPD strategy. **(A)** Structure of diphyllin–PROTAC. **(B)** Content of triglycerides of AML 12 cells treated with 1.5 mM FFA and diphyllin or diphyllin–PROTAC for 24 h. For all, n = 3–4 for all groups. **(C)** Analyses of protein–protein interaction (PPI) networks in L02 cells treated with diphyllin–PROTAC for 24 h. **(D)** Blotting and quantification of p65 in L02 cells treated with diphyllin–PROTAC for 24 h. **(E)** Blotting and quantification of p65 protein stability in L02 cells treated with 40 μM diphyllin under different temperature. For **(C–E)**, all blotting assays were repeated three times. Bar graphs are presented as mean ± SEM, and one-way ANOVA was used for all comparisons. **(F)** Model of the interaction between diphyllin and p65.

Before applying the TPD strategy, we assessed the bioactivity of D-P in improving lipid metabolism. As the results indicate, the lowering effects of diphyllin and D-P were similar in AML 12 cells cultured with free fatty acids for 24 h, indicating that chemical modification of diphyllin did not affect the bioactive activity in cell-based assays (Figures 4A, B). Then, we treated cells with DMSO or D-P for 24 h, extracted total proteins, and performed TMT-labeled quantitative proteomics analysis. Among 780 identified proteins, 439 showed significant differential expression (fold change > 1.2, p < 0.05), including 178 downregulated proteins (Supplementary Table S2). Gene Ontology (GO) enrichment analysis indicated that differentially expressed proteins were associated with biological processes such as positive regulation of NF-κB transcription, translation regulation, endoplasmic reticulum stress response, and ATP metabolism (Supplementary Figures S3A, S3B). In terms of molecular functions, the proteins were enriched in mRNA binding, GDP binding, nucleosome DNA binding, and NF-κB binding.

Protein–protein interaction (PPI) networks, constructed using the STRING database and visualized with Cytoscape 3.10.2, highlighted key hub proteins with high connectivity in the network (Figure 4C). Notably, p65 (RELA, a subunit of NF-κB) emerged as a downregulated hub protein, suggesting its potential significance (Figure 4D). To validate whether diphyllin directly interacts with p65, we conducted cellular thermal shift assays (CESTA). The results showed that p65 exhibited increased stability in the presence of diphyllin with an increase in the temperature, indicating a potential direct interaction between diphyllin and p65 (Figure 4E). This finding suggests that p65 may serve as a primary target of diphyllin, warranting further investigation.

Building on the above experiments, we further investigated the interaction between diphyllin and the p65 protein to elucidate its molecular mechanism. Using AutoDock, we performed global docking of diphyllin with p65 (PDB: 1NFI). The docking grid encompassed the entire p65 protein, and four potential binding sites for diphyllin were identified on the protein surface. To refine these findings, molecular dynamics (MD) simulations were conducted for the docked complexes to determine the optimal binding site. After 1 μs of MD simulation, diphyllin was stably positioned within a pocket at the base of p65. The interaction was primarily stabilized by hydrogen bonds and π–π interactions. Specifically, the 7-hydroxyl group of diphyllin formed strong hydrogen bonds with G44, while the methoxy group on the A-ring interacted with S42. The lactone carbonyl group contributed to the complex’s stability through hydrogen bond water bridges with N115. Notably, the methylenedioxy group on the B-ring engaged in a cation–π interaction with the positively charged guanidinium group of R41, further stabilizing the complex (Figure 4F). These findings highlight the key molecular interactions driving the diphyllin–p65 binding and provide valuable insights into its functional mechanism.

**FIGURE 5:**
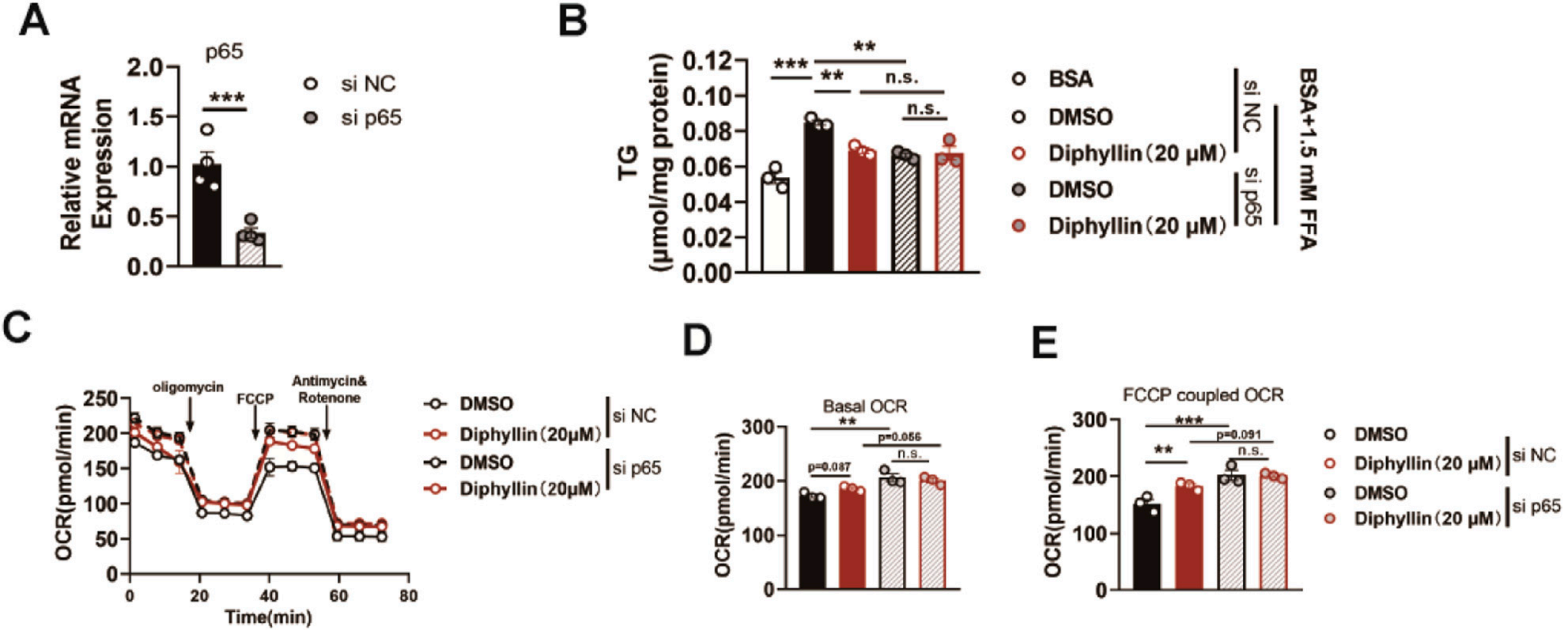
P65 is the target protein of diphyllin regulating lipid metabolism. **(A)** p65 gene expression in AML 12 cells treated with si p65 for 24 h. **(B)** Content of triglyceride in AML 12 cells treated with diphyllin combined with si p65 or not for 24 h. **(C)** Curve of the oxygen consumption rate of AML 12 cells treated with diphyllin combined with si p65 or not for 24 h. **(D)** Basal oxygen consumption rate. **(E)** Oxygen consumption rate of AML 12 cells treated with FCCP. For all, n = 3–4 for all groups. Bar graphs are presented as mean ± SEM, and one-way ANOVA was used for all comparisons.

Then, we determined whether the effects of diphyllin on lipid metabolism were dependent on p65. We constructed siRNA to knockdown p65 in AML 12 cells, validated by p65 mRNA examination (Figure 5A). Once p65 deficiency AML 12 cells were obtained, the effects of diphyllin on lipid accumulation were blocked (Figure 5B). Moreover, the induction of oxidative metabolism was also blocked by p65 knockdown (Figures 5C–E). These findings indicated that the effects of diphyllin on lipid metabolism were dependent on p65.

### Diphyllin activates NRF2 transcription activity to promote fatty acid oxidation

**FIGURE 6:**
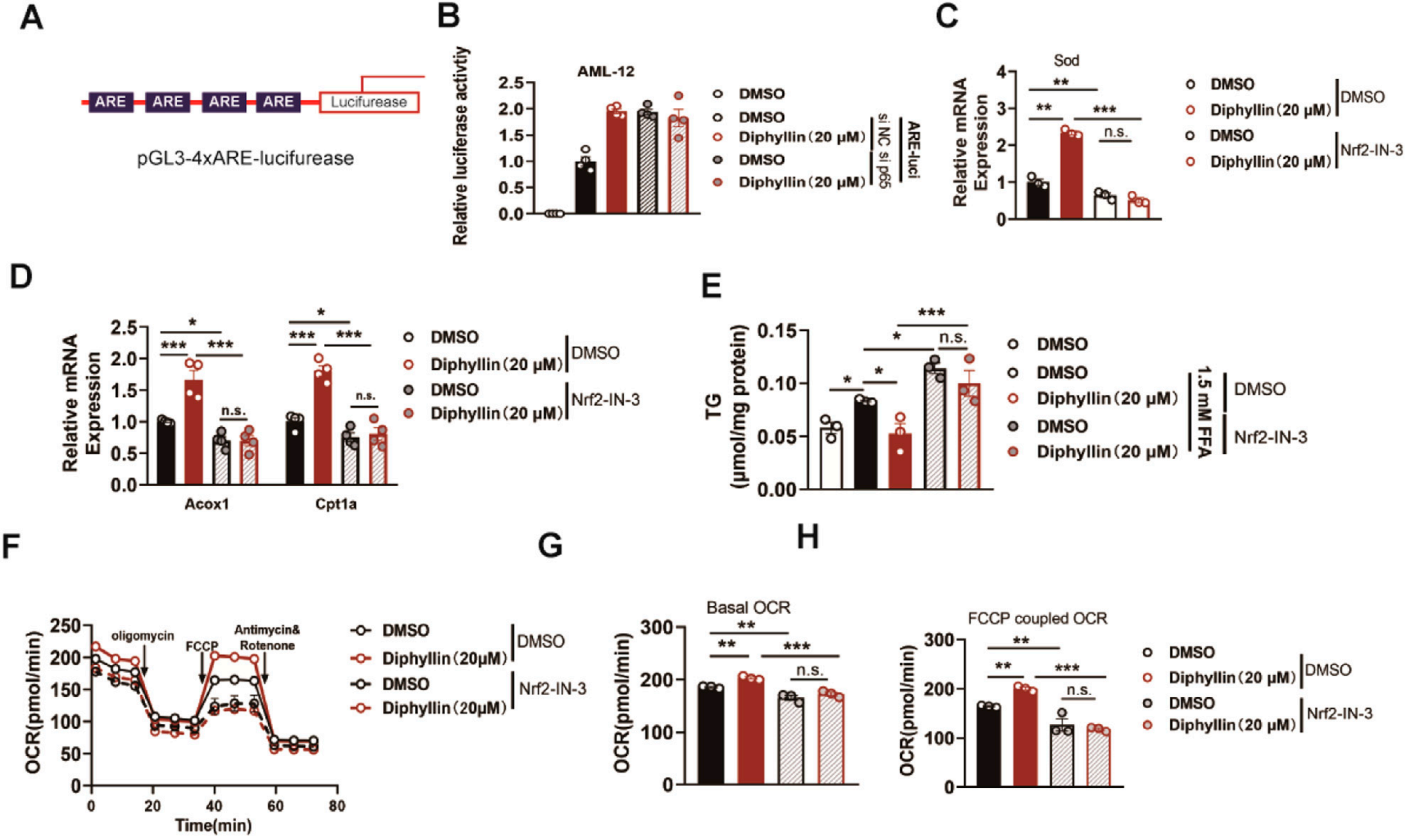
Effect of diphyllin on lipid metabolism depends on NRF2. **(A)** Structure of 4xARE luciferase plasmid. **(B)** Luciferase activity of AML 12 cells with p65 knockdown transfected with pGL-3 4xARE luciferase plasmid and treated with diphyllin for 24 h. **(C)** Sod gene expression in AML 12 cells treated with diphyllin combined with NRF2 inhibitor (10 μM Nrf2-IN-30) or not for 24 h. **(D)** Acox1 and Cpt1α gene expression in AML 12 cells treated with diphyllin together with NRF2 inhibitor (10 μM Nrf2-IN-30) or not for 24 h. **(E)** Content of triglycerides in AML 12 cells treated with diphyllin combined with the NRF2 inhibitor (10 μM Nrf2-IN-30) or not for 24 h. **(F)** Curve of the oxygen consumption rate of AML 12 cells treated with diphyllin combined with the NRF2 inhibitor (10 μM Nrf2-IN-30) or not for 24 h. **(G)** Basal oxygen consumption rate. **(H)** Oxygen consumption rate of AML 12 cells treated with FCCP. For all, n = 3–4 for all groups. Bar graphs are presented as mean ± SEM, and one-way ANOVA was used for all comparisons.

p65 is the core regulator for the inflammation pathways, and how p65 regulates lipid metabolism in hepatocytes. It has been reported that the p65 antagonist affects the NRF2 transcriptional pathway. This effect might induce oxidative damage, consequently leading to a reduction in mitochondrial oxidative metabolism. Then, we assessed the NRF2 anti-oxidative response elements (AREs) to determine the effects of diphyllin on transcriptional activity (Figure 6A). AML 12 cells were transfected with ARE-luciferase plasmids and then cultured with diphyllin. We found that diphyllin induced ARE-luciferase activity, which was blocked by p65 knockdown (Figure 6B). Then, we detected the expression of the NRF2 downstream gene, superoxide dismutase (SOD), in AML 12 cells treated by diphyllin. Once NRF2 was inhibited by NRF2-IN-3, the effect of diphyllin on sod expression was blocked (Figure 6C). Consistently, the impacts of diphyllin on lipid oxidative gene expression, lipid accumulation, and oxidative metabolic capacity were blocked with the inhibition of NRF2 (Figures 6D–H), indicating that the effect of diphyllin on fatty acid oxidative effects was dependent on the p65/NRF2 pathway.

## Discussion

MASLD affects up to a third of the global population; its burden has grown in parallel with the increasing rates of type 2 diabetes mellitus and obesity. The pathogenesis of MASLD is complex and involves the dysfunction of lipid uptake, *de novo* lipogenesis, export, and oxidation, as well as mitochondrial oxidative metabolic dysfunction, endoplasmic reticulum stress, and inflammatory responses. Hepatic steatosis is the primary pathological feature of MASLD. As for MASLD therapy, rectifying the dysfunctions in hepatic lipid metabolism is as a potentially efficacious means. Emerging therapies targeting specific pathways in hepatic lipid metabolism are under clinical investigation, such as peroxisome proliferator-activated receptor (PPAR) and stearoyl-CoA desaturase 1 (SCD 1) (Sangro et al., 2023). In our study, we found that diphyllin, as a natural product, had a protective effect on the development of MASLD. Based on *in vivo* and *in vitro* studies, we determined that diphyllin treatment induced the capacity of hepatic fatty acid oxidation, resulting in the inhibition of hepatic steatosis-associated MASLD development. Therefore, diphyllin could potentially serve as a regulator of hepatic lipid metabolism, holding promise for having a therapeutic effect on MASLD.

The targeted protein degradation (TPD) technology utilized in our study was developed based on the ubiquitin–proteasome system (UPS). Its mechanism of action specifically targets the proteasome-mediated degradation pathway, without interfering with lysosomal or peroxisomal protein degradation pathways. Consequently, the diphyllin-targeting proteins identified through this technique were substrates that depend on the proteasome for degradation. It remains unclear whether there are potential target proteins in the liver that can interact with diphyllin and be degraded via non-proteasomal pathways, such as autophagy–lysosomal pathways.

Our approach focuses on leveraging a widely studied generic PROTAC design using CRBN as the E3 ubiquitin ligase, pomalidomide as the ligand, and a triethylene glycol linker. Instead of constructing a PROTAC toolbox, we analyzed the natural cellular responses following protein degradation, which disrupts the regulatory protein network. By integrating proteomics with bioinformatics, we visualized differentially expressed proteins within the regulatory network and prioritized downregulated proteins at key nodes for validation. Unlike traditional strategies that focus on highly differential proteins, our method targets proteins with significant regulatory impacts, aligning with the multi-target nature of natural products while identifying functionally relevant mechanisms. Sørensen et al. (2007) found that diphyllin, as a novel and naturally potent vacuolar H + -ATPase (V-ATPase) inhibitor, abrogates acidification of the osteoclastic resorption lacunae and bone resorption. Inhibition of V-ATPase causes lysosomal deacidification, resulting in the reduction of autophagy flux (Cypess et al., 2012; Shen et al., 2011). It has been reported that inhibition of autophagy modulates major pathological changes, including hepatic lipid metabolism, inflammation, and fibrosis, in the development of MASLD (Chen and Lin, 2022). However, V-ATPase decrease was not observed in the proteome data of diphyllin–PROTAC. Thus, under the condition of MASLD, p65 might be the main target protein for the protective effects of diphyllin.

As we know, NF-κB/p65, which is a critical regulator of immune and inflammatory responses, serves as an important transcription factor (Giridharan and Srinivasan, 2018). During MASLD progression, liver inflammation and fibrosis are involved. Diphyllin treatment also reduces liver inflammation and fibrosis in a MASH mouse model. Hepatocytes are the predominant cell type in the liver. Given the remarkable effect of diphyllin on lipid accumulation, our initial focus is to explore its impact on hepatocytes. As a result, we did not investigate the direct effect of diphyllin on Kupffer cells or hepatic stellate cells (HSCs) within the liver. Additionally, it has been reported that p65 has an impact on metabolic regulation. Hepatic p65 knockout mice fed with HFD exhibited an improvement in systemic insulin sensitivity (Ke et al., 2015). In the liver with p65 knockdown by siRNA, a decrease in hepatic lipid accumulation was observed in HFD-fed mice (Zeng et al., 2016b), which builds the linkage between p65 and hepatic lipid metabolism. Moreover, it has been reported that p65 could interact with PGC-1α and inhibit its transcriptional activity, resulting in a decrease in the mitochondrial content and increase in hepatic steatosis in mice fed with HFDs (Zhou et al., 2013). In our study, we determined that the effects of diphyllin on lipid metabolism depend on p65. Nuclear factor erythroid 2-related factor 2 (NRF2) is a transcription factor that regulates the cellular defense against oxidative insults through the expression of genes involved in oxidative stress response (He et al., 2020). It has been reported that NF-κB/p65 was the negative regulator of NRF2-ARE signaling, leading to cellular oxidative damage (Liu et al., 2008; Yu et al., 2011). Oxidative damage-induced mitochondria dysfunction also contributes to hepatic steatosis, inflammation, and fibrosis in MASLD (Garcia-Berumen et al., 2022; Tang et al., 2021). In our study, we found that diphyllin interacted with p62 and increased NRF2-ARE signaling, which contributed to the improvement in mitochondrial function. NRF2 deficiency abolished the inhibitory effects of diphyllin on lipid metabolism. However, further investigation is required to clarify whether its molecular docking pattern and binding region directly interfere with key p65 phosphorylation sites, such as Ser536. Thus, our research determined the role of diphyllin in regulating hepatic lipid metabolism via the p65/NRF2 pathway.

Collectively, our study determined that chronic diphyllin administration alleviated CDAHFD-induced MASLD by decreasing hepatic steatosis, inflammation, and fibrosis. Based on the proteolysis-targeting chimeras and proteome, p65 was identified as the target of diphyllin. Diphyllin interacted with p65 and relieved the inhibition of NRF2, resulting in antioxidant effects and an increase in the capacity of fatty acid oxidation in hepatocytes. Our results revealed that diphyllin is a promising lead compound for the treatment of MASLD and provided a potential strategy for MASLD therapy.

## Data Availability

The datasets presented in this study can be found in online repositories. The names of the repository/repositories and accession number(s) can be found in the article/Supplementary Material.

## Abbreviations

MASLD, metabolic dysfunction-associated steatotic liver disease; MASH, metabolic dysfunction-associated steatohepatitis; ROS, reactive oxygen species; NRF2, nuclear factor (erythroid-derived 2)-like 2; KEAP1, Kelch-like ECH-associated protein-1; AREs, antioxidant response elements; MCD, methionine- and choline-deficient; TPD, targeted protein degradation; CDAHFD, choline-deficient, L-amino acid-defined, high-fat diet; LDH, lactate dehydrogenase; AST, aspartate aminotransferase; ALT, alanine aminotransferase; TG, triglyceride; TC, cholesterol; Cpt1a, carnitine palmitoyltransferase 1A; Acox1, acyl-coenzyme A oxidase 1; Acc1, acetyl-CoA carboxylase 1; FASN, fatty acid synthase; Sod, superoxide dismutase; PPAR, peroxisome proliferator-activated receptor; SCD 1, stearoyl-CoA desaturase 1.
