# Supplementary material for: Unveiling p65 as the target of diphyllin in ameliorating metabolic dysfunction-associated steatotic liver disease via targeted protein degradation technology
Source: Front Pharmacol. 2025 Apr 28;16:1567639. doi: 10.3389/fphar.2025.1567639 (PMC12066529; doi:10.3389/fphar.2025.1567639)
Supplement: Supplementary file 1 [file DataSheet1.docx]

**Supplementary information**

**Unveiling p65 as the target of diphyllin in ameliorating metabolic dysfunction-associated steatotic liver disease via targeted protein degradation technology**

Scheme S1.

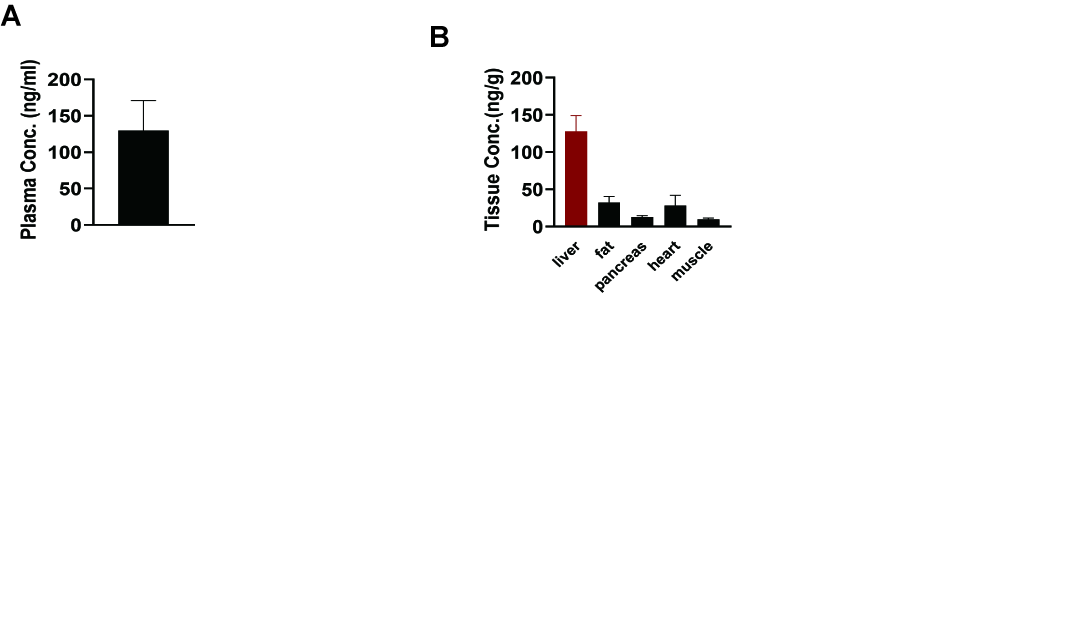


**Figure S1 The plasma and tissue distribution of diphyllin in mice.** (A-B) The concentration of diphyllin in plasma and tissue was detected in 2.5 hours of mice orally treated with 100 mg/kg diphyllin. n=2 for all groups. Bar graphs are presented as mean ± SEM.


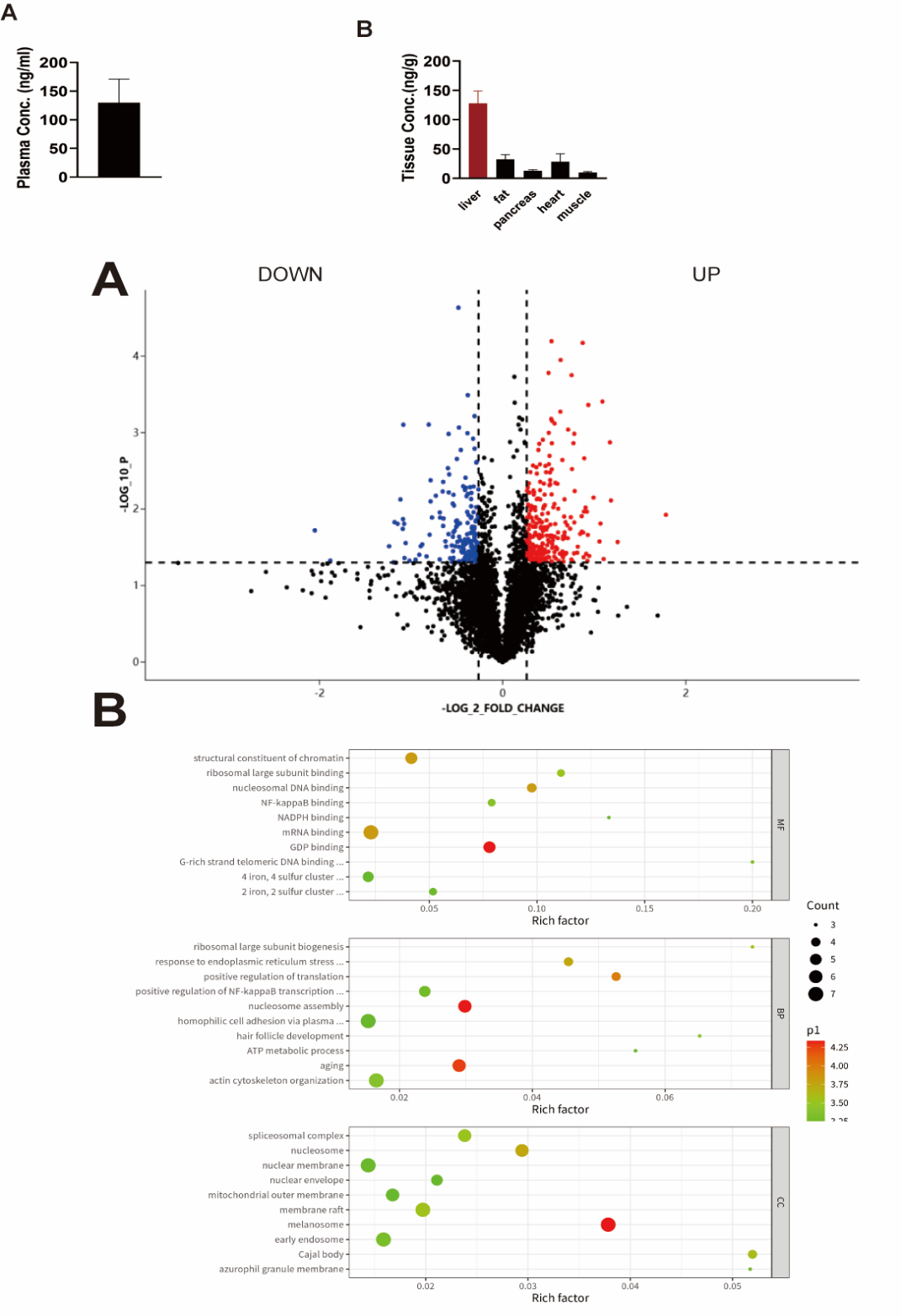


**Figure S2 Analyses of database of diphyllin-PROTAC treated cells.** (A) The volcano plot of protein changes by diphyllin-PROTAC in L02 cells. (B) Gene Ontology (GO) enrichment analysis of protein changes by diphyllin-PROTAC in L02 cells.

**Figure S3 1H NMR spectrum of 2-bromo-N-(2-(2,6-dioxopiperidin-3-yl)-1,3-dioxoisoindolin-4-yl)acetamide (1a)**

**Figure S4 13C NMR spectrum of 2-bromo-N-(2-(2,6-dioxopiperidin-3-yl)-1,3-dioxoisoindolin-4-yl)acetamide (1a)**

 **Figure S5 1H NMR spectrum of 2-azido-N-(2-(2,6-dioxopiperidin-3-yl)-1,3-dioxoisoindolin-4-yl)acetamide (1b)**

**Figure S6 13C NMR spectrum of 2-azido-N-(2-(2,6-dioxopiperidin-3-yl)-1,3-dioxoisoindolin-4-yl)acetamide (1b)**

**Figure S7 13C NMR spectrum of propargyl-PEG3-OH (3a)**

**Figure S8 13C NMR spectrum of propargyl-PEG3-OH (3a)**

**Figure S9 1H NMR spectrum of diphyllin-7-O-PEG3-propyne (2a)**

**Figure S10 1H NMR spectrum of Diphyllin-PROTAC (D-P)**

**Figure S11 13C NMR spectrum of Diphyllin-PROTAC (D-P)**

**Table S1. The primers sequence**

| Gene | Forward | Reverse |
| --- | --- | --- |
| Acc1 | GTCTGCTGGGAAGTTAATCCAG | TCCTGCAGCTCTAGCAGAGG |
| Fasn | GGAGGTGGTGATAGCCGGTAT | TGGGTAATCCATAGAGCCCAG |
| Cpt1a | CTCCGCCTGAGCCATGAAG | CACCAGTGATGATGCCATTCT |
| Acox1 | CTCCCACTCTGGTCTTCCTG | GGTGTAAAAGGTGGCCTGAA |
| Fibronectin | CCCTATCTCTGATACCGTTGTCC | TGCCGCAACTACTGTGATTCGG |
| Col1a1 | GAGCGGAGAGTACTGGATCG | TACTCGAACGGGAATCCATC |
| α-Sma | CTGACAGAGGCACCACTGAA | CATCTCCAGAGTCCAGCACA |
| Col3a1 | ATTCTGCCACCCCGAACTCAA | ACAGTCATGGGGCTGGCATTT |
| F4/80 | ATGGACAAACCAACTTTCAAGGC | GCAGACTGAGTTAGGACCACAA |
| Il1b | GCAACTGTTCCTGAACTCAACT | ATCTTTTGGGGTCCGTCAACT |
| Tnfa | CTGAACTTCGGGGTGATCGG | GGCTTGTCACTCGAATTTTGAGA |
| Tgfb1 | ACCATGCCAACTTCTGTCTGGGAC | ACAACTGCTCCACCTTGGGCTTG |
| Sod | GCACATTAACGCGCAGATCA | AGCCTCCAGCAACTCTCCTT |
| Gapdh | CATCACTGCCACCCAGAAGACTG | ATGCCAGTGAGCTTCCCGTTCAG |

**Table S2. The protein database**

| ID | Description | Score | Coverage | mean(DP) | mean(NC) | Fold_change(DP/NC) | log_2_fold_change | p | log_10_p | type |
| --- | --- | --- | --- | --- | --- | --- | --- | --- | --- | --- |
| P05783 | Keratin, type I cytoskeletal 18 OS=Homo sapiens OX=9606 GN=KRT18 PE=1 SV=2 - [K1C18_HUMAN] | 8177.50722981216 | 73.02 | 1.01723170750085 | 0.802026977559685 | 1.26832604882689 | 0.342925667053792 | 0.03327556330541 | 1.47787458383644 | up |
| P02545 | Prelamin-A/C OS=Homo sapiens OX=9606 GN=LMNA PE=1 SV=1 - [LMNA_HUMAN] | 6586.14623580805 | 63.7 | 1.0588621169915 | 0.863787801373937 | 1.22583592325254 | 0.293765888856553 | 0.00610996775445076 | 2.21396108175435 | up |
| Q13707 | ACTA2 protein (Fragment) OS=Homo sapiens OX=9606 GN=ACTA2 PE=3 SV=1 - [Q13707_HUMAN] | 5422.34512782292 | 37.27 | 1.03893187419935 | 0.769257662087881 | 1.35056421976939 | 0.433562242097927 | 0.0446070961397875 | 1.3505960478078 | up |
| P06748 | Nucleophosmin OS=Homo sapiens OX=9606 GN=NPM1 PE=1 SV=2 - [NPM_HUMAN] | 4841.74155421946 | 58.84 | 1.11772708058537 | 0.78823185278848 | 1.41801815878317 | 0.503876007484829 | 0.00100835805925552 | 2.99638522627672 | up |
| B4DLR3 | cDNA FLJ54020, highly similar to Heterogeneous nuclear ribonucleoprotein U OS=Homo sapiens OX=9606 PE=2 SV=1 - [B4DLR3_HUMAN] | 4631.62960579297 | 43.37 | 1.13359979896154 | 0.803805911024959 | 1.41029044874284 | 0.495992315679776 | 0.0254094738664014 | 1.59500432744409 | up |
| B3KX72 | cDNA FLJ44920 fis, clone BRAMY3011501, highly similar to Heterogeneous nuclear ribonucleoprotein U OS=Homo sapiens OX=9606 PE=2 SV=1 - [B3KX72_HUMAN] | 4499.64492987044 | 45.07 | 1.11216269054762 | 0.887643554646321 | 1.25293839483886 | 0.325315481183782 | 0.0424839662316371 | 1.37177494503144 | up |
| P02786 | Transferrin receptor protein 1 OS=Homo sapiens OX=9606 GN=TFRC PE=1 SV=2 - [TFR1_HUMAN] | 4390.00285498505 | 53.68 | 1.10102322153575 | 0.758831520639109 | 1.45094555456583 | 0.536993384579312 | 0.00302554490625478 | 2.51919639672464 | up |
| P22626 | Heterogeneous nuclear ribonucleoproteins A2/B1 OS=Homo sapiens OX=9606 GN=HNRNPA2B1 PE=1 SV=2 - [ROA2_HUMAN] | 4322.84489555578 | 52.41 | 1.06758136374418 | 0.854104015929162 | 1.24994303250381 | 0.321862344009684 | 0.00929079761147549 | 2.03194700038817 | up |
| O60814 | Histone H2B type 1-K OS=Homo sapiens OX=9606 GN=H2BC12 PE=1 SV=3 - [H2B1K_HUMAN] | 3923.30209671079 | 82.54 | 1.11307051654763 | 0.752905939957002 | 1.47836596509146 | 0.564003448555617 | 0.0419175380475808 | 1.37760423279315 | up |
| Q99877 | Histone H2B type 1-N OS=Homo sapiens OX=9606 GN=H2BC15 PE=1 SV=3 - [H2B1N_HUMAN] | 3923.30209671079 | 82.54 | 1.1529228166742 | 0.685004716569825 | 1.68308741353999 | 0.751110107033708 | 0.0456092371242161 | 1.34094719185716 | up |
| B4DR52 | Histone H2B OS=Homo sapiens OX=9606 PE=1 SV=1 - [B4DR52_HUMAN] | 3923.30209671079 | 63.86 | 1.16449505062146 | 0.741602911137449 | 1.57024066806236 | 0.650985695429338 | 0.040399339773923 | 1.39362573228754 | up |
| Q99880 | Histone H2B type 1-L OS=Homo sapiens OX=9606 GN=H2BC13 PE=1 SV=3 - [H2B1L_HUMAN] | 3908.65716331157 | 82.54 | 1.1389410274124 | 0.721069747876435 | 1.5795157552603 | 0.659482327665693 | 0.0246147755729269 | 1.60880411958587 | up |
| P52272 | Heterogeneous nuclear ribonucleoprotein M OS=Homo sapiens OX=9606 GN=HNRNPM PE=1 SV=3 - [HNRPM_HUMAN] | 3170.48767365744 | 59.04 | 1.06642517730125 | 0.823659883945606 | 1.29473973188146 | 0.372662117014866 | 0.0131499846342878 | 1.88107475464555 | up |
| P05186 | Alkaline phosphatase, tissue-nonspecific isozyme OS=Homo sapiens OX=9606 GN=ALPL PE=1 SV=4 - [PPBT_HUMAN] | 3136.49996582375 | 38.55 | 1.04555879113979 | 0.855443219437033 | 1.22224218672033 | 0.28953018283358 | 0.0117074413942896 | 1.93153800755403 | up |
| B4DUQ1 | Heterogeneous nuclear ribonucleoprotein K OS=Homo sapiens OX=9606 PE=1 SV=1 - [B4DUQ1_HUMAN] | 3007.38825998195 | 51.48 | 1.1002727696514 | 0.822779538989012 | 1.33726316408324 | 0.419283405713701 | 0.0310940462148558 | 1.50732276028406 | up |
| A0A172Q3A8 | Folate receptor (Fragment) OS=Homo sapiens OX=9606 PE=2 SV=1 - [A0A172Q3A8_HUMAN] | 2787.97726386902 | 49.76 | 1.1163250920359 | 0.737552213830197 | 1.51355398452225 | 0.597940133251698 | 0.00466696544282403 | 2.33096541489509 | up |
| F5GZS6 | 4F2 cell-surface antigen heavy chain OS=Homo sapiens OX=9606 GN=SLC3A2 PE=1 SV=1 - [F5GZS6_HUMAN] | 2782.0297598737 | 32.55 | 1.04390840442702 | 0.867367337852545 | 1.20353667802567 | 0.26728010890791 | 0.00576986839645491 | 2.23883409245003 | up |
| A0A7P0T937 | Calnexin OS=Homo sapiens OX=9606 GN=CANX PE=1 SV=1 - [A0A7P0T937_HUMAN] | 2620.98236612984 | 36.04 | 1.0145455720736 | 0.81888827651736 | 1.23893039034378 | 0.30909513152453 | 0.0129330735397458 | 1.88829825294412 | up |
| Q12771 | p37 AUF1 OS=Homo sapiens OX=9606 PE=2 SV=1 - [Q12771_HUMAN] | 1754.61514888093 | 47.2 | 1.06415788335441 | 0.744182235474499 | 1.42996947874722 | 0.515984354465248 | 0.0221734887476473 | 1.654165970185 | up |
| A8K8D9 | Glucose-6-phosphate 1-dehydrogenase OS=Homo sapiens OX=9606 PE=2 SV=1 - [A8K8D9_HUMAN] | 1617.91002191634 | 42.14 | 0.943368651227805 | 1.16536096132836 | 0.809507682626066 | -0.304883322409019 | 0.0440077989899563 | 1.35647035173744 | down |
| P27105 | Stomatin OS=Homo sapiens OX=9606 GN=STOM PE=1 SV=3 - [STOM_HUMAN] | 1377.75499881943 | 44.1 | 1.05921656407372 | 0.842919668720864 | 1.25660439942169 | 0.329530536137887 | 0.01672247472662 | 1.77669945174236 | up |
| A0A0C4DGS1 | Dolichyl-diphosphooligosaccharide--protein glycosyltransferase 48 kDa subunit OS=Homo sapiens OX=9606 GN=DDOST PE=1 SV=1 - [A0A0C4DGS1_HUMAN] | 1046.30252957023 | 20.27 | 1.05188155916845 | 0.848685246409846 | 1.23942482047163 | 0.309670764831544 | 0.0316824235743792 | 1.49918160408 | up |
| B4DR70 | cDNA FLJ58049, highly similar to RNA-binding protein FUS OS=Homo sapiens OX=9606 PE=2 SV=1 - [B4DR70_HUMAN] | 1032.85842941747 | 23.31 | 1.09384812704097 | 0.834654301129601 | 1.31054033455597 | 0.390161756236652 | 0.0460304620746854 | 1.33695466543888 | up |
| Q96RS2 | 40S ribosomal protein SA OS=Homo sapiens OX=9606 GN=RPSA PE=2 SV=1 - [Q96RS2_HUMAN] | 985.971071673211 | 26.44 | 1.0526631130961 | 0.815232440996401 | 1.29124291448646 | 0.368760432526314 | 0.00283978192288173 | 2.54671500971548 | up |
| Q9Y3F4 | Serine-threonine kinase receptor-associated protein OS=Homo sapiens OX=9606 GN=STRAP PE=1 SV=1 - [STRAP_HUMAN] | 956.012148453052 | 40.86 | 0.905257964844174 | 1.19942686877007 | 0.754742109264616 | -0.405944326267795 | 0.0261165600515675 | 1.58308402682292 | down |
| A8K2T7 | Receptor protein-tyrosine kinase OS=Homo sapiens OX=9606 PE=2 SV=1 - [A8K2T7_HUMAN] | 938.789810602686 | 18.68 | 1.04299969460684 | 0.866842489345586 | 1.20321708664079 | 0.266896959692097 | 0.016009698265093 | 1.79561685315503 | up |
| A0A0B4J1Z1 | Serine/arginine-rich-splicing factor 7 OS=Homo sapiens OX=9606 GN=SRSF7 PE=1 SV=1 - [A0A0B4J1Z1_HUMAN] | 896.176785812203 | 70.07 | 1.04844235090991 | 0.8667338275961 | 1.20964743445837 | 0.274586618841665 | 0.00521984621484097 | 2.28234229183103 | up |
| A0A384MTQ5 | Terpene cyclase/mutase family member OS=Homo sapiens OX=9606 PE=2 SV=1 - [A0A384MTQ5_HUMAN] | 844.593182076059 | 16.53 | 1.0895321999029 | 0.854198842820596 | 1.27550184486931 | 0.351064985637277 | 0.021839775950418 | 1.66075182127848 | up |
| A8K6A6 | cDNA FLJ78619, highly similar to Homo sapiens melanoma cell adhesion molecule (MCAM), mRNA OS=Homo sapiens OX=9606 PE=2 SV=1 - [A8K6A6_HUMAN] | 771.432079265427 | 22.91 | 1.06923152504037 | 0.858582153284909 | 1.24534562120761 | 0.316546189525423 | 0.0129864833519294 | 1.8865084368884 | up |
| Q86UE4 | Protein LYRIC OS=Homo sapiens OX=9606 GN=MTDH PE=1 SV=2 - [LYRIC_HUMAN] | 770.676023731242 | 26.12 | 1.09511342868821 | 0.773721122521066 | 1.41538520380565 | 0.501194742803292 | 0.000166132941629047 | 3.77954424503651 | up |
| A0A7P0TA85 | Basigin OS=Homo sapiens OX=9606 GN=BSG PE=1 SV=1 - [A0A7P0TA85_HUMAN] | 768.305379408542 | 43.23 | 1.06371729672401 | 0.801098005173444 | 1.32782417363992 | 0.409064122127838 | 0.00566914708825458 | 2.24648227492143 | up |
| B4DZY9 | cDNA FLJ59103, highly similar to T-complex protein 1 subunit epsilon OS=Homo sapiens OX=9606 PE=2 SV=1 - [B4DZY9_HUMAN] | 721.827461539586 | 40.99 | 0.776286965962106 | 1.61644580992104 | 0.48024311189252 | -1.05816317336464 | 0.0278294047253408 | 1.55549608318319 | down |
| Q96PK6 | RNA-binding protein 14 OS=Homo sapiens OX=9606 GN=RBM14 PE=1 SV=2 - [RBM14_HUMAN] | 708.88742246937 | 24.36 | 1.06164879669362 | 0.880019540841534 | 1.20639229860555 | 0.270699123839371 | 0.0215589734358691 | 1.6663719226018 | up |
| Q92522 | Histone H1.10 OS=Homo sapiens OX=9606 GN=H1-10 PE=1 SV=1 - [H1X_HUMAN] | 691.1130461588 | 37.56 | 0.946368031613654 | 1.20442258283712 | 0.785744177416866 | -0.347868418595676 | 0.0279514273571993 | 1.55359600969071 | down |
| Q15738 | Sterol-4-alpha-carboxylate 3-dehydrogenase, decarboxylating OS=Homo sapiens OX=9606 GN=NSDHL PE=1 SV=2 - [NSDHL_HUMAN] | 688.567115398508 | 32.44 | 1.0697532923264 | 0.801073084475189 | 1.33540036865329 | 0.417272343616857 | 0.0171312074190723 | 1.76621202658727 | up |
| P08754 | Guanine nucleotide-binding protein G(i) subunit alpha-3 OS=Homo sapiens OX=9606 GN=GNAI3 PE=1 SV=3 - [GNAI3_HUMAN] | 687.557587822061 | 33.33 | 1.0831994546771 | 0.799886557990304 | 1.35419134608115 | 0.437431604893962 | 0.0361189032815579 | 1.44226544512483 | up |
| C1PHA2 | Tyrosine-protein kinase receptor OS=Homo sapiens OX=9606 GN=KIF5B-ALK PE=2 SV=1 - [C1PHA2_HUMAN] | 686.065039435416 | 12.95 | 0.947434060511984 | 1.16528920958967 | 0.813046283030114 | -0.298590614176894 | 0.0406397693835474 | 1.39104876518617 | down |
| H3BLV0 | Complement decay-accelerating factor (Fragment) OS=Homo sapiens OX=9606 GN=CD55 PE=1 SV=1 - [H3BLV0_HUMAN] | 679.291447372155 | 34.05 | 1.10533500234558 | 0.808613585355089 | 1.36695081849285 | 0.450961337170109 | 0.0046474914324569 | 2.33278140216717 | up |
| A0A8I5KV85 | Cold shock domain-containing protein E1 OS=Homo sapiens OX=9606 GN=CSDE1 PE=4 SV=1 - [A0A8I5KV85_HUMAN] | 667.123924563988 | 27.11 | 0.925869782130867 | 1.16836344561418 | 0.792450145206451 | -0.335607919920313 | 0.0176907469446982 | 1.75225382977472 | down |
| P63096 | Guanine nucleotide-binding protein G(i) subunit alpha-1 OS=Homo sapiens OX=9606 GN=GNAI1 PE=1 SV=2 - [GNAI1_HUMAN] | 590.011237458972 | 24.01 | 1.07897778248291 | 0.765269825674502 | 1.40993117235729 | 0.495624737289004 | 0.0113213847970123 | 1.94610044833165 | up |
| Q7L2H7 | Eukaryotic translation initiation factor 3 subunit M OS=Homo sapiens OX=9606 GN=EIF3M PE=1 SV=1 - [EIF3M_HUMAN] | 587.085870790741 | 22.19 | 0.906294339615575 | 1.13393364965943 | 0.799248121693692 | -0.323284646343099 | 0.0188395572349843 | 1.72492930816006 | down |
| P51151 | Ras-related protein Rab-9A OS=Homo sapiens OX=9606 GN=RAB9A PE=1 SV=1 - [RAB9A_HUMAN] | 571.783333333333 | 28.86 | 1.0782061738049 | 0.799098349471994 | 1.34927843927763 | 0.432188095887466 | 0.0116333121601403 | 1.93429661819681 | up |
| A0A024R394 | Cysteine and histidine-rich domain (CHORD)-containing 1, isoform CRA_c OS=Homo sapiens OX=9606 GN=CHORDC1 PE=4 SV=1 - [A0A024R394_HUMAN] | 553.380991299194 | 43.07 | 0.923061403882424 | 1.24157918725919 | 0.743457536462176 | -0.427677751647908 | 0.0299450342680606 | 1.52367518558792 | down |
| Q7Z3Z9 | L1 cell adhesion molecule (Fragment) OS=Homo sapiens OX=9606 GN=L1CAM PE=2 SV=1 - [Q7Z3Z9_HUMAN] | 550.620190192469 | 12.35 | 1.1371652163532 | 0.734473468817155 | 1.54827269415813 | 0.630659592840154 | 0.000533032031782665 | 3.27324669189564 | up |
| Q9H3R2 | Mucin-13 OS=Homo sapiens OX=9606 GN=MUC13 PE=1 SV=3 - [MUC13_HUMAN] | 542.629003782402 | 12.89 | 1.08844571609344 | 0.790579965067908 | 1.37676865615984 | 0.461286158128833 | 0.00889677454611997 | 2.05076741478372 | up |
| Q9NZ45 | CDGSH iron-sulfur domain-containing protein 1 OS=Homo sapiens OX=9606 GN=CISD1 PE=1 SV=1 - [CISD1_HUMAN] | 518.419021749783 | 40.74 | 0.92682346618336 | 1.18437675679823 | 0.78254107982402 | -0.353761605969545 | 0.0483839365972877 | 1.31529879961763 | down |
| Q10471 | Polypeptide N-acetylgalactosaminyltransferase 2 OS=Homo sapiens OX=9606 GN=GALNT2 PE=1 SV=1 - [GALT2_HUMAN] | 506.9872977356 | 19.26 | 1.05576830176275 | 0.860803702893535 | 1.22649135710483 | 0.294537067482194 | 0.0270532289516971 | 1.56778089202824 | up |
| A8K8A6 | cDNA FLJ76931, highly similar to Homo sapiens testes-specific heterogenous nuclear ribonucleoprotein G-T, mRNA OS=Homo sapiens OX=9606 PE=2 SV=1 - [A8K8A6_HUMAN] | 495.037619406888 | 13.01 | 0.96166117563261 | 1.28059492805208 | 0.750948761834779 | -0.413213620661361 | 0.0291079811829745 | 1.53598791450271 | down |
| B3KRY3 | cDNA FLJ35079 fis, clone PLACE6005283, highly similar to Lysosome-associated membrane glycoprotein 1 OS=Homo sapiens OX=9606 PE=2 SV=1 - [B3KRY3_HUMAN] | 492.198823529412 | 9.62 | 1.06950060755462 | 0.835191550265336 | 1.28054529193315 | 0.35675828088679 | 0.0103695649844418 | 1.98423946239777 | up |
| Q9BY44 | Eukaryotic translation initiation factor 2A OS=Homo sapiens OX=9606 GN=EIF2A PE=1 SV=3 - [EIF2A_HUMAN] | 471.147391304348 | 18.97 | 0.941856657198965 | 1.15554373296868 | 0.815076600155376 | -0.294992446008329 | 0.0318644814019237 | 1.49669314531892 | down |
| E9PNM1 | Squalene synthase OS=Homo sapiens OX=9606 GN=FDFT1 PE=1 SV=1 - [E9PNM1_HUMAN] | 464.478274517021 | 23.66 | 1.08748411662775 | 0.729552262184647 | 1.49061852453349 | 0.57591109385193 | 0.0045383549814656 | 2.34310153743693 | up |
| C9J0K6 | Sorcin OS=Homo sapiens OX=9606 GN=SRI PE=1 SV=1 - [C9J0K6_HUMAN] | 443.757153970974 | 31.61 | 0.899572362076893 | 1.27896306031111 | 0.703360706804209 | -0.507663353728687 | 0.0498707898625166 | 1.30215375329325 | down |
| A0A7I2YQP9 | Eukaryotic translation initiation factor 3 subunit I OS=Homo sapiens OX=9606 GN=EIF3I PE=1 SV=1 - [A0A7I2YQP9_HUMAN] | 435.76359448433 | 35.05 | 0.918578030585706 | 1.22296183849505 | 0.751109316473919 | -0.412905201984474 | 0.0250579760260374 | 1.6010540106053 | down |
| P16435 | NADPH--cytochrome P450 reductase OS=Homo sapiens OX=9606 GN=POR PE=1 SV=2 - [NCPR_HUMAN] | 385.791408541299 | 16.1 | 1.05412064373014 | 0.860750066881425 | 1.22465357168 | 0.292373699348144 | 0.0453494352745898 | 1.34342811673303 | up |
| C9JIJ5 | 60S ribosomal protein L7 (Fragment) OS=Homo sapiens OX=9606 GN=RPL7 PE=1 SV=1 - [C9JIJ5_HUMAN] | 383.533098003504 | 53.54 | 1.09853627521174 | 0.4602121227144 | 2.38702159502537 | 1.25521161832379 | 0.0269831603289355 | 1.56890718613044 | up |
| Q8NBU5 | Outer mitochondrial transmembrane helix translocase OS=Homo sapiens OX=9606 GN=ATAD1 PE=1 SV=1 - [ATAD1_HUMAN] | 381.447595703749 | 23.82 | 1.04037241798614 | 0.848691899320323 | 1.22585406885505 | 0.293787244387513 | 0.0161170212538299 | 1.79271522137483 | up |
| P61221 | ATP-binding cassette sub-family E member 1 OS=Homo sapiens OX=9606 GN=ABCE1 PE=1 SV=1 - [ABCE1_HUMAN] | 378.896831214827 | 24.04 | 0.922643156448304 | 1.1668814848108 | 0.7906914013619 | -0.338813359221731 | 0.0206099160183839 | 1.6859237778873 | down |
| A0A1Y6MTK5 | MHC class I antigen OS=Homo sapiens OX=9606 GN=HLA-A PE=3 SV=1 - [A0A1Y6MTK5_HUMAN] | 375.64909233478 | 26.03 | 1.07804991656717 | 0.723990695992594 | 1.48903835716998 | 0.574380917779094 | 0.0156826070846961 | 1.80458173831103 | up |
| Q16352 | Alpha-internexin OS=Homo sapiens OX=9606 GN=INA PE=1 SV=2 - [AINX_HUMAN] | 375.498235294118 | 6.81 | 1.06351562873367 | 0.881114691903623 | 1.20701157125864 | 0.27143950684475 | 0.0367845729255089 | 1.43433428181726 | up |
| O00767 | Stearoyl-CoA desaturase OS=Homo sapiens OX=9606 GN=SCD PE=1 SV=2 - [SCD_HUMAN] | 373.881230943891 | 14.21 | 1.1409033349325 | 0.663298713321833 | 1.72004454707713 | 0.782445929470995 | 0.00103651504347616 | 2.9844243903851 | up |
| E9PK91 | Bcl-2-associated transcription factor 1 OS=Homo sapiens OX=9606 GN=BCLAF1 PE=1 SV=1 - [E9PK91_HUMAN] | 367.48829314907 | 17.68 | 1.10234672907167 | 0.772825155147977 | 1.42638567304476 | 0.512364117377931 | 0.00201435297148782 | 2.69586442646464 | up |
| Q9UQ35 | Serine/arginine repetitive matrix protein 2 OS=Homo sapiens OX=9606 GN=SRRM2 PE=1 SV=2 - [SRRM2_HUMAN] | 357.801571410038 | 7.05 | 1.08299328843684 | 0.845348623358591 | 1.28112030765968 | 0.35740596288803 | 0.0203200182183922 | 1.69207590701097 | up |
| P27694 | Replication protein A 70 kDa DNA-binding subunit OS=Homo sapiens OX=9606 GN=RPA1 PE=1 SV=2 - [RFA1_HUMAN] | 353.500845165973 | 18.34 | 0.931021513028903 | 1.12179266130383 | 0.829940812722741 | -0.268919640639946 | 0.0425935829663009 | 1.37065582559792 | down |
| A5YRV2 | MUC1 isoform T11 OS=Homo sapiens OX=9606 GN=MUC1 PE=2 SV=1 - [A5YRV2_HUMAN] | 347.084444444444 | 19.35 | 1.11486708144058 | 0.648249659168917 | 1.7198112882464 | 0.782250269318378 | 0.0149578099948204 | 1.82513198780767 | up |
| P61011 | Signal recognition particle 54 kDa protein OS=Homo sapiens OX=9606 GN=SRP54 PE=1 SV=1 - [SRP54_HUMAN] | 345.478013305706 | 17.66 | 0.912523964788491 | 1.18167662766865 | 0.772228157367232 | -0.372900935384252 | 0.0119470570304052 | 1.92273906315637 | down |
| Q8IV08 | 5'-3' exonuclease PLD3 OS=Homo sapiens OX=9606 GN=PLD3 PE=1 SV=1 - [PLD3_HUMAN] | 345.364107421467 | 21.43 | 1.08240966141657 | 0.825683080930822 | 1.31092629413737 | 0.390586573376786 | 0.00168973997552647 | 2.77218012135226 | up |
| A0A8I5QJM4 | Magnesium transporter protein 1 OS=Homo sapiens OX=9606 GN=MAGT1 PE=4 SV=1 - [A0A8I5QJM4_HUMAN] | 340.030104434101 | 18.54 | 1.04808079129338 | 0.872994712476806 | 1.20055800603859 | 0.263705110359265 | 0.0250611405182862 | 1.60099916839665 | up |
| Q9NTK5 | Obg-like ATPase 1 OS=Homo sapiens OX=9606 GN=OLA1 PE=1 SV=2 - [OLA1_HUMAN] | 334.327383456542 | 21.46 | 0.954544478688183 | 1.1501023093928 | 0.829964839556001 | -0.268877875141831 | 0.00936214381561246 | 2.02862469178717 | down |
| P21291 | Cysteine and glycine-rich protein 1 OS=Homo sapiens OX=9606 GN=CSRP1 PE=1 SV=3 - [CSRP1_HUMAN] | 317.505327178311 | 53.37 | 0.966253022311681 | 1.1965576478106 | 0.807527346534184 | -0.308416978063806 | 0.0392470586553205 | 1.40619288560993 | down |
| A8K556 | cDNA FLJ78217 OS=Homo sapiens OX=9606 PE=2 SV=1 - [A8K556_HUMAN] | 296.42 | 13.73 | 1.09382802399853 | 0.763095752718538 | 1.43340861235533 | 0.519449927805101 | 0.00418236997112257 | 2.37857755226677 | up |
| P05026 | Sodium/potassium-transporting ATPase subunit beta-1 OS=Homo sapiens OX=9606 GN=ATP1B1 PE=1 SV=1 - [AT1B1_HUMAN] | 295.323333333333 | 23.76 | 1.0701721331355 | 0.825560324288364 | 1.29629792233292 | 0.374397324455348 | 0.00343993701366868 | 2.46344950942456 | up |
| A0A8I5KRR9 | 7-dehydrocholesterol reductase OS=Homo sapiens OX=9606 GN=DHCR7 PE=4 SV=1 - [A0A8I5KRR9_HUMAN] | 294.568117786115 | 17.16 | 1.10259564352183 | 0.726043170085775 | 1.51863647913879 | 0.602776568649527 | 0.0239904547226994 | 1.61996152019859 | up |
| Q8TCJ2 | Dolichyl-diphosphooligosaccharide--protein glycosyltransferase subunit STT3B OS=Homo sapiens OX=9606 GN=STT3B PE=1 SV=1 - [STT3B_HUMAN] | 288.743971978141 | 9.56 | 1.0681455878546 | 0.823161461303681 | 1.29761369800151 | 0.375860954272124 | 0.0405994906621437 | 1.39147941479739 | up |
| Q9BXY0 | Protein MAK16 homolog OS=Homo sapiens OX=9606 GN=MAK16 PE=1 SV=2 - [MAK16_HUMAN] | 282.12 | 7.67 | 0.911042792088469 | 1.27294778617783 | 0.715695334860496 | -0.482582519166714 | 2.32855111961625e-05 | 4.63291422338572 | down |
| B3KT00 | Zinc phosphodiesterase ELAC protein 2 OS=Homo sapiens OX=9606 PE=2 SV=1 - [B3KT00_HUMAN] | 276.501222968645 | 8.55 | 0.930119344977385 | 1.14241811448216 | 0.814167189040939 | -0.296603012885903 | 0.0261482166980732 | 1.58255792456326 | down |
| O75955 | Flotillin-1 OS=Homo sapiens OX=9606 GN=FLOT1 PE=1 SV=3 - [FLOT1_HUMAN] | 276.045 | 14.99 | 1.03618668652055 | 0.850361768878745 | 1.21852454383835 | 0.285135310492739 | 0.0317876519475161 | 1.49774155084895 | up |
| P18583 | Protein SON OS=Homo sapiens OX=9606 GN=SON PE=1 SV=4 - [SON_HUMAN] | 268.541379687815 | 3.75 | 1.05820143488829 | 0.871733002789369 | 1.2139054406593 | 0.27965604465364 | 0.0290119030239229 | 1.53742378289392 | up |
| A0A1B0GW44 | Cathepsin D OS=Homo sapiens OX=9606 GN=CTSD PE=1 SV=1 - [A0A1B0GW44_HUMAN] | 266.728214913782 | 18.77 | 0.945520457933399 | 1.1703668986211 | 0.807883800411128 | -0.30778029278416 | 0.0231410281473607 | 1.63561734941167 | down |
| Q9H3N1 | Thioredoxin-related transmembrane protein 1 OS=Homo sapiens OX=9606 GN=TMX1 PE=1 SV=1 - [TMX1_HUMAN] | 266.602926357849 | 17.5 | 1.01601890060306 | 0.842211072722451 | 1.20637086534469 | 0.270673492099283 | 0.0264420789042926 | 1.57770440315122 | up |
| P04080 | Cystatin-B OS=Homo sapiens OX=9606 GN=CSTB PE=1 SV=2 - [CYTB_HUMAN] | 263.148571428571 | 51.02 | 0.963117442117113 | 1.16638633004633 | 0.825727648984753 | -0.276262081100472 | 0.0411543559107956 | 1.38558419087931 | down |
| A8K3S0 | cDNA FLJ76127, highly similar to Homo sapiens replication factor C (activator 1) 5, 36.5kDa (RFC5), transcript variant 1, mRNA OS=Homo sapiens OX=9606 PE=2 SV=1 - [A8K3S0_HUMAN] | 261.95684789687 | 15.59 | 0.93008446834568 | 1.13101101726237 | 0.82234784113506 | -0.282179332936004 | 0.0283096665078958 | 1.54806524665869 | down |
| Q86Y74 | CROP protein (Fragment) OS=Homo sapiens OX=9606 GN=CROP PE=2 SV=1 - [Q86Y74_HUMAN] | 251.234851762232 | 30.68 | 1.0253734361461 | 0.839195293989938 | 1.22185317707274 | 0.289070935373372 | 0.0408696349805436 | 1.38859924109055 | up |
| Q13501 | Sequestosome-1 OS=Homo sapiens OX=9606 GN=SQSTM1 PE=1 SV=1 - [SQSTM_HUMAN] | 250.651311528621 | 9.09 | 1.16545936785078 | 0.555616823428775 | 2.09759553474029 | 1.06873651953629 | 0.0154835137512255 | 1.8101304758493 | up |
| B4DVZ8 | Leukotriene A(4) hydrolase OS=Homo sapiens OX=9606 PE=2 SV=1 - [B4DVZ8_HUMAN] | 244.743670076389 | 17.47 | 0.91293644057955 | 1.23076797057927 | 0.741761617463828 | -0.430972477528028 | 0.0293348721926329 | 1.53261579959543 | down |
| Q5BKZ1 | DBIRD complex subunit ZNF326 OS=Homo sapiens OX=9606 GN=ZNF326 PE=1 SV=2 - [ZN326_HUMAN] | 237.697899055856 | 12.54 | 1.09151365153045 | 0.884671074873141 | 1.23380732402376 | 0.303117115412035 | 0.00329113575819052 | 2.48265420284176 | up |
| Q5JTV8 | Torsin-1A-interacting protein 1 OS=Homo sapiens OX=9606 GN=TOR1AIP1 PE=1 SV=2 - [TOIP1_HUMAN] | 236.250041586111 | 12.69 | 1.07844355567964 | 0.794515314585652 | 1.35736031248442 | 0.440803736257768 | 0.0193850941304216 | 1.7125320857982 | up |
| P16989 | Y-box-binding protein 3 OS=Homo sapiens OX=9606 GN=YBX3 PE=1 SV=4 - [YBOX3_HUMAN] | 233.58 | 12.37 | 1.13051237821124 | 0.789805600608406 | 1.43138055407606 | 0.517407285264284 | 0.0033194610141757 | 2.47893242761287 | up |
| A8KA19 | Exportin-T OS=Homo sapiens OX=9606 PE=2 SV=1 - [A8KA19_HUMAN] | 229.308149824668 | 6.34 | 0.921896139170767 | 1.27067169278292 | 0.725518750757485 | -0.462915194206281 | 0.0254846138571495 | 1.59372194245509 | down |
| P21281 | V-type proton ATPase subunit B, brain isoform OS=Homo sapiens OX=9606 GN=ATP6V1B2 PE=1 SV=3 - [VATB2_HUMAN] | 224.067787580379 | 19.57 | 1.08213285775817 | 0.852598867842493 | 1.26921685985405 | 0.343938590775906 | 0.00605704252466615 | 2.21773937727561 | up |
| A0A6I8PS32 | DnaJ homolog subfamily C member 7 (Fragment) OS=Homo sapiens OX=9606 GN=DNAJC7 PE=1 SV=1 - [A0A6I8PS32_HUMAN] | 223.078584690077 | 22.7 | 0.918279694121537 | 1.17998745665303 | 0.778211402963713 | -0.361765974922684 | 0.0470254561487249 | 1.32766698308405 | down |
| Q9NVJ2 | ADP-ribosylation factor-like protein 8B OS=Homo sapiens OX=9606 GN=ARL8B PE=1 SV=1 - [ARL8B_HUMAN] | 222.254994284481 | 20.43 | 1.06085065119922 | 0.830171351411072 | 1.27786950175533 | 0.35374051329329 | 0.0366091166502159 | 1.43641075018335 | up |
| F2Z2V0 | Copine-1 (Fragment) OS=Homo sapiens OX=9606 GN=CPNE1 PE=1 SV=1 - [F2Z2V0_HUMAN] | 218.045555555556 | 12.38 | 0.924263427661997 | 1.12725456554629 | 0.819924315155981 | -0.286437350020659 | 0.0444820412487329 | 1.35181529150873 | down |
| B2RDD7 | Protein arginine N-methyltransferase 5 OS=Homo sapiens OX=9606 PE=2 SV=1 - [B2RDD7_HUMAN] | 217.979942426354 | 12.4 | 0.941977082148769 | 1.15251863617957 | 0.817320477585758 | -0.291026213865141 | 0.0371212472789353 | 1.43037743985462 | down |
| B3KVN0 | Solute carrier family 2, facilitated glucose transporter member 1 OS=Homo sapiens OX=9606 PE=2 SV=1 - [B3KVN0_HUMAN] | 217.73 | 6.97 | 1.14167452381262 | 0.684837737137513 | 1.66707303336494 | 0.737317309222595 | 0.018055398126779 | 1.74339293081196 | up |
| Q04941 | Proteolipid protein 2 OS=Homo sapiens OX=9606 GN=PLP2 PE=1 SV=1 - [PLP2_HUMAN] | 209.71353427499 | 8.55 | 1.15679841832513 | 0.718701739453323 | 1.6095667435076 | 0.686672401935796 | 0.038566717493973 | 1.41378732340826 | up |
| B7Z8A2 | cDNA FLJ51671, highly similar to Prenylcysteine oxidase OS=Homo sapiens OX=9606 PE=2 SV=1 - [B7Z8A2_HUMAN] | 196.647825128414 | 10.27 | 1.01874035026776 | 0.811951287205872 | 1.25468161245671 | 0.327321312857049 | 0.0355032812137061 | 1.44973150758844 | up |
| P18859 | ATP synthase-coupling factor 6, mitochondrial OS=Homo sapiens OX=9606 GN=ATP5PF PE=1 SV=1 - [ATP5J_HUMAN] | 196.370211356575 | 37.96 | 0.999812540008611 | 0.784766164198336 | 1.27402605466553 | 0.349394781952452 | 0.0308660125917618 | 1.51051947097936 | up |
| I3L1P8 | Mitochondrial 2-oxoglutarate/malate carrier protein (Fragment) OS=Homo sapiens OX=9606 GN=SLC25A11 PE=1 SV=1 - [I3L1P8_HUMAN] | 195.143646945908 | 27.36 | 1.02901454153471 | 0.839815433658321 | 1.22528653355681 | 0.29311916327121 | 0.0362989814987414 | 1.44010556051986 | up |
| H3BPZ1 | Very-long-chain (3R)-3-hydroxyacyl-CoA dehydratase OS=Homo sapiens OX=9606 GN=HACD3 PE=1 SV=1 - [H3BPZ1_HUMAN] | 195.059907277824 | 11.87 | 1.04635966910694 | 0.784661139760662 | 1.33351789210067 | 0.415237181973004 | 0.0393270324065352 | 1.405308823946 | up |
| B4DE93 | NADH dehydrogenase [ubiquinone] flavoprotein 1, mitochondrial OS=Homo sapiens OX=9606 GN=NDUFV1 PE=1 SV=1 - [B4DE93_HUMAN] | 192.858593140528 | 11.29 | 0.934076806487061 | 1.13624093316856 | 0.822076356536695 | -0.282655693582913 | 0.00242583428216342 | 2.61513887074076 | down |
| Q86UA8 | SMARCA1 protein (Fragment) OS=Homo sapiens OX=9606 GN=SMARCA1 PE=2 SV=2 - [Q86UA8_HUMAN] | 191.576664622304 | 9.84 | 1.01557908495548 | 0.82151504366595 | 1.23622700860539 | 0.305943689939343 | 0.0467428449416074 | 1.33028485837852 | up |
| B4DTT0 | N-acetylglucosamine-6-sulfatase OS=Homo sapiens OX=9606 PE=2 SV=1 - [B4DTT0_HUMAN] | 188.031246501548 | 8.96 | 0.859269319548487 | 1.34346113295476 | 0.639593731795309 | -0.644772294831055 | 0.0132910147420212 | 1.87644186028851 | down |
| E9PKH6 | NADH dehydrogenase [ubiquinone] iron-sulfur protein 8, mitochondrial (Fragment) OS=Homo sapiens OX=9606 GN=NDUFS8 PE=1 SV=1 - [E9PKH6_HUMAN] | 180.095424877252 | 18.12 | 0.919547695303155 | 1.21434658189578 | 0.757236615157761 | -0.401183922581385 | 0.0052703588921647 | 2.27815980991465 | down |
| J3KT68 | Sigma intracellular receptor 2 OS=Homo sapiens OX=9606 GN=TMEM97 PE=1 SV=1 - [J3KT68_HUMAN] | 178.995 | 25 | 1.06917770084072 | 0.619867788975827 | 1.72484797541627 | 0.786469211294756 | 0.00581420663634845 | 2.23550953750691 | up |
| B2R7M1 | V-type proton ATPase subunit OS=Homo sapiens OX=9606 PE=2 SV=1 - [B2R7M1_HUMAN] | 177.074358295705 | 11.4 | 1.06773433456818 | 0.865438308056777 | 1.2337497943275 | 0.30304984417693 | 0.0103645308676916 | 1.98445035070314 | up |
| Q8NCD8 | cDNA FLJ90316 fis, clone NT2RP2001495, weakly similar to Human transporter protein (g17) OS=Homo sapiens OX=9606 PE=2 SV=1 - [Q8NCD8_HUMAN] | 174.294285714286 | 6.3 | 1.07998535045992 | 0.675940419862791 | 1.59775228514837 | 0.676043750762532 | 0.0201207993095902 | 1.6963547706912 | up |
| B4DEP8 | Phosphatidylinositol 4-kinase type 2 OS=Homo sapiens OX=9606 PE=2 SV=1 - [B4DEP8_HUMAN] | 170.419562391586 | 5.57 | 1.06203368044784 | 0.780593481373267 | 1.36054643779429 | 0.444186199095625 | 0.00978870267032787 | 2.00927486289067 | up |
| B7ZKQ9 | SCARB1 protein OS=Homo sapiens OX=9606 GN=SCARB1 PE=1 SV=1 - [B7ZKQ9_HUMAN] | 169.595330659884 | 12.89 | 1.05017753996426 | 0.833944654741683 | 1.2592892513827 | 0.332609699741487 | 0.00269970238508687 | 2.56868410978125 | up |
| F8VV56 | CD63 antigen OS=Homo sapiens OX=9606 GN=CD63 PE=1 SV=1 - [F8VV56_HUMAN] | 167.268570296275 | 28.97 | 1.13288266789091 | 0.730243390353225 | 1.55137681882054 | 0.633549149698174 | 0.000112417247869022 | 3.94916705104742 | up |
| Q9BPW0 | Serine/threonine-protein phosphatase (Fragment) OS=Homo sapiens OX=9606 GN=PPP5C PE=2 SV=2 - [Q9BPW0_HUMAN] | 166.67 | 9.92 | 0.932960238480594 | 1.29892273476856 | 0.718256916680136 | -0.477428114178523 | 0.0480032470156681 | 1.31872938526413 | down |
| Q8IVL5 | Prolyl 3-hydroxylase 2 OS=Homo sapiens OX=9606 GN=P3H2 PE=1 SV=1 - [P3H2_HUMAN] | 165.35 | 4.8 | 1.0852225170939 | 0.90076769678772 | 1.20477512788699 | 0.268763891553022 | 0.040160181602976 | 1.39620433199371 | up |
| A5YKK6 | CCR4-NOT transcription complex subunit 1 OS=Homo sapiens OX=9606 GN=CNOT1 PE=1 SV=2 - [CNOT1_HUMAN] | 165.004909930113 | 2.99 | 0.924855332311218 | 1.11218131511486 | 0.831568845602935 | -0.266092385676271 | 0.0143203651369727 | 1.84404590835842 | down |
| P28838 | Cytosol aminopeptidase OS=Homo sapiens OX=9606 GN=LAP3 PE=1 SV=3 - [AMPL_HUMAN] | 161.06 | 10.79 | 0.95482911631569 | 1.17522967420023 | 0.812461714741397 | -0.299628263710092 | 0.0348452060909755 | 1.45785696251419 | down |
| P49006 | MARCKS-related protein OS=Homo sapiens OX=9606 GN=MARCKSL1 PE=1 SV=2 - [MRP_HUMAN] | 160.41 | 11.28 | 1.05984569593334 | 0.880415197228643 | 1.20380213707067 | 0.267598283023604 | 0.0129468932497312 | 1.88783443284671 | up |
| B7Z591 | Calcium load-activated calcium channel OS=Homo sapiens OX=9606 GN=TMCO1 PE=2 SV=1 - [B7Z591_HUMAN] | 157.978524282094 | 25 | 1.04957108479723 | 0.854186432784244 | 1.22873771405632 | 0.297176991370631 | 0.0448497589830866 | 1.34823988645705 | up |
| D3DT44 | GCS light chain OS=Homo sapiens OX=9606 GN=GCLM PE=3 SV=1 - [D3DT44_HUMAN] | 157.57 | 8.27 | 0.850024970623076 | 1.44997904599689 | 0.586232589339708 | -0.770454923564261 | 0.0128983381060792 | 1.88946624302281 | down |
| Q9Y6H1 | Coiled-coil-helix-coiled-coil-helix domain-containing protein 2 OS=Homo sapiens OX=9606 GN=CHCHD2 PE=1 SV=1 - [CHCH2_HUMAN] | 157.223333333333 | 18.54 | 1.0805672397129 | 0.826027881927828 | 1.30814862712747 | 0.387526463953933 | 0.00730262467490555 | 2.13652101974828 | up |
| Q10589 | Bone marrow stromal antigen 2 OS=Homo sapiens OX=9606 GN=BST2 PE=1 SV=1 - [BST2_HUMAN] | 156.8 | 11.67 | 0.943987064809249 | 1.15360705564102 | 0.81829168796537 | -0.289312897478146 | 0.0489800876375563 | 1.30998044214979 | down |
| B2R6S2 | cDNA, FLJ93084, highly similar to Homo sapiens mannose-6-phosphate receptor (cation dependent) (M6PR), mRNA OS=Homo sapiens OX=9606 PE=2 SV=1 - [B2R6S2_HUMAN] | 150.68720810507 | 16.97 | 1.05209405910964 | 0.830719758262786 | 1.26648493507581 | 0.340829916197131 | 0.00647455607822185 | 2.18879000321394 | up |
| E7ET76 | Glutathione hydrolase 1 proenzyme (Fragment) OS=Homo sapiens OX=9606 GN=GGT1 PE=1 SV=1 - [E7ET76_HUMAN] | 150.52 | 5.83 | 1.05637470917071 | 0.865518526515577 | 1.2205107999519 | 0.287485061172412 | 0.00461108450926284 | 2.33619691821213 | up |
| Q9NSB2 | Keratin, type II cuticular Hb4 OS=Homo sapiens OX=9606 GN=KRT84 PE=2 SV=2 - [KRT84_HUMAN] | 149.270956368879 | 7.33 | 1.10186676100741 | 0.819800130595273 | 1.3440675597445 | 0.426605657207963 | 0.0149038422673858 | 1.82670175437902 | up |
| E9PR30 | FAU ubiquitin-like and ribosomal protein S30 OS=Homo sapiens OX=9606 GN=FAU PE=1 SV=1 - [E9PR30_HUMAN] | 143.715714285714 | 22.45 | 1.08228754589309 | 0.684717262727909 | 1.5806342337292 | 0.660503559830891 | 0.0227645284077528 | 1.64274134215052 | up |
| Q5BKY2 | Eukaryotic translation initiation factor 3 subunit H OS=Homo sapiens OX=9606 GN=EIF3H PE=2 SV=1 - [Q5BKY2_HUMAN] | 138.318414462365 | 19.48 | 0.948452830206309 | 1.19418469331705 | 0.794226249519096 | -0.332378051545495 | 0.0170204745347685 | 1.76902833585199 | down |
| A0A068F4C8 | Glucosylceramidase OS=Homo sapiens OX=9606 PE=2 SV=1 - [A0A068F4C8_HUMAN] | 137.676666666667 | 5.78 | 1.05215345824849 | 0.837309638653235 | 1.25658825561929 | 0.329512001479496 | 0.0317795113881986 | 1.49785278437975 | up |
| K4DIA7 | Tetraspanin (Fragment) OS=Homo sapiens OX=9606 GN=CD151 PE=1 SV=1 - [K4DIA7_HUMAN] | 137.102857142857 | 16.96 | 1.09563762452674 | 0.812369009542891 | 1.34869451155362 | 0.431563605086279 | 0.0220539707520568 | 1.65651320572201 | up |
| A8K274 | cDNA FLJ78227, highly similar to Homo sapiens pituitary tumor-transforming 1 interacting protein (PTTG1IP), mRNA OS=Homo sapiens OX=9606 PE=2 SV=1 - [A8K274_HUMAN] | 136.519263302691 | 20.11 | 1.14573218740843 | 0.625035515848896 | 1.8330673351455 | 0.874259782220539 | 6.72141612473798e-05 | 4.17253921647496 | up |
| G3V192 | Ferritin OS=Homo sapiens OX=9606 GN=FTH1 PE=1 SV=1 - [G3V192_HUMAN] | 134.93 | 21.57 | 1.13366881966588 | 0.721299204905502 | 1.57170396411903 | 0.652329506580764 | 0.00228113433649175 | 2.64184913827479 | up |
| B7ZLC9 | GEMIN5 protein OS=Homo sapiens OX=9606 GN=GEMIN5 PE=2 SV=1 - [B7ZLC9_HUMAN] | 134.040759108485 | 5.04 | 0.934523140394713 | 1.28196912615416 | 0.728974763376894 | -0.456059224616658 | 0.00169109899385741 | 2.77183096884955 | down |
| Q96JB5 | CDK5 regulatory subunit-associated protein 3 OS=Homo sapiens OX=9606 GN=CDK5RAP3 PE=1 SV=2 - [CK5P3_HUMAN] | 133.202022521253 | 6.52 | 0.948536807962388 | 1.16243331476499 | 0.81599244955755 | -0.293372291999754 | 0.0216024649015036 | 1.6654966918099 | down |
| P05114 | Non-histone chromosomal protein HMG-14 OS=Homo sapiens OX=9606 GN=HMGN1 PE=1 SV=3 - [HMGN1_HUMAN] | 131.342857142857 | 42 | 1.0817460171659 | 0.730182069240374 | 1.48147436473107 | 0.567033662279389 | 0.000759675721234085 | 3.1193717531568 | up |
| Q13951 | Core-binding factor subunit beta OS=Homo sapiens OX=9606 GN=CBFB PE=1 SV=2 - [PEBB_HUMAN] | 131.333840544862 | 17.58 | 0.944132110978601 | 1.13308447262941 | 0.833240710454422 | -0.2631947666275 | 0.00554683402029632 | 2.25595482946061 | down |
| Q53GB9 | Microsomal glutathione S-transferase 3 variant (Fragment) OS=Homo sapiens OX=9606 PE=2 SV=1 - [Q53GB9_HUMAN] | 124.89 | 8.55 | 1.09600224775365 | 0.882591987049722 | 1.2417994541479 | 0.312432202650502 | 0.0465915417125757 | 1.33169291852395 | up |
| P62330 | ADP-ribosylation factor 6 OS=Homo sapiens OX=9606 GN=ARF6 PE=1 SV=2 - [ARF6_HUMAN] | 122.092857142857 | 12.57 | 1.02709639947769 | 0.828476279916258 | 1.23974146801344 | 0.310039296658453 | 0.0462914701920893 | 1.33449902603999 | up |
| C9J712 | Profilin OS=Homo sapiens OX=9606 GN=PFN2 PE=1 SV=1 - [C9J712_HUMAN] | 120.281542290941 | 37.36 | 0.897738892244242 | 1.26026709044715 | 0.712340184909309 | -0.489361716232353 | 0.0158695858343288 | 1.79943440734803 | down |
| P62487 | DNA-directed RNA polymerase II subunit RPB7 OS=Homo sapiens OX=9606 GN=POLR2G PE=1 SV=1 - [RPB7_HUMAN] | 119.713333333333 | 12.21 | 0.927541214127107 | 1.16352797826777 | 0.797179983164657 | -0.327022609687361 | 0.0340319944246344 | 1.46811259859058 | down |
| A0A0C4DFM1 | Transmembrane 9 superfamily member OS=Homo sapiens OX=9606 GN=TM9SF4 PE=1 SV=1 - [A0A0C4DFM1_HUMAN] | 116.575714285714 | 7.84 | 1.05997627968488 | 0.796263557828732 | 1.33118773208113 | 0.412714043108416 | 0.0318075685243403 | 1.49746952852929 | up |
| O43653 | Prostate stem cell antigen OS=Homo sapiens OX=9606 GN=PSCA PE=1 SV=2 - [PSCA_HUMAN] | 115.483333333333 | 8.77 | 1.09333789240465 | 0.508824164624213 | 2.14875386905439 | 1.10350023742785 | 0.0449100518568673 | 1.3476564434646 | up |
| A0A7P0T989 | Cathepsin X OS=Homo sapiens OX=9606 GN=CTSZ PE=1 SV=1 - [A0A7P0T989_HUMAN] | 114.628203266126 | 10.03 | 0.920538733561691 | 1.23409443549192 | 0.745922440850127 | -0.422902464430891 | 0.0337185731531054 | 1.47213081141514 | down |
| B2R6P4 | cDNA, FLJ93048, highly similar to Homo sapiens synaptobrevin-like 1 (SYBL1), mRNA OS=Homo sapiens OX=9606 PE=2 SV=1 - [B2R6P4_HUMAN] | 108.516564532428 | 19.09 | 1.03096062409642 | 0.820922941420983 | 1.25585554024336 | 0.328670522063359 | 0.0289924671753537 | 1.53771482586436 | up |
| P05204 | Non-histone chromosomal protein HMG-17 OS=Homo sapiens OX=9606 GN=HMGN2 PE=1 SV=3 - [HMGN2_HUMAN] | 104.976864651572 | 50 | 1.18387820660582 | 0.625500105282193 | 1.89269065921534 | 0.920438636670629 | 0.0108043082226715 | 1.96640303486074 | up |
| A0A024RAJ6 | Beta-hexosaminidase OS=Homo sapiens OX=9606 GN=HEXB PE=2 SV=1 - [A0A024RAJ6_HUMAN] | 104.139735668909 | 7.01 | 0.884539527885524 | 1.28310006686833 | 0.689376885502335 | -0.536635168288657 | 0.0200294422677748 | 1.69833114373688 | down |
| J3KMY5 | NPC intracellular cholesterol transporter 2 OS=Homo sapiens OX=9606 GN=NPC2 PE=1 SV=1 - [J3KMY5_HUMAN] | 103.704913685534 | 23.65 | 0.888584673835364 | 1.42969426129217 | 0.621520766987101 | -0.686125497770885 | 0.0456109143266315 | 1.34093122170709 | down |
| Q8IVS2 | Malonyl-CoA-acyl carrier protein transacylase, mitochondrial OS=Homo sapiens OX=9606 GN=MCAT PE=1 SV=2 - [FABD_HUMAN] | 103.69 | 2.82 | 0.999850578939864 | 0.738773359020788 | 1.35339284603484 | 0.436580667668293 | 0.0391212958671234 | 1.4075867679279 | up |
| P05362 | Intercellular adhesion molecule 1 OS=Homo sapiens OX=9606 GN=ICAM1 PE=1 SV=2 - [ICAM1_HUMAN] | 103.397717310309 | 5.45 | 1.07925296716473 | 0.808149847570154 | 1.33546144989039 | 0.417338331005087 | 0.0177632626093024 | 1.75047726360903 | up |
| Q9BYC9 | 39S ribosomal protein L20, mitochondrial OS=Homo sapiens OX=9606 GN=MRPL20 PE=1 SV=1 - [RM20_HUMAN] | 102.93 | 12.08 | 1.01668602825065 | 0.742479708973019 | 1.36931153264364 | 0.453450712095651 | 0.017324631977348 | 1.76133598222291 | up |
| A0A7I2V610 | Insulin-degrading enzyme OS=Homo sapiens OX=9606 GN=IDE PE=1 SV=1 - [A0A7I2V610_HUMAN] | 101.637370085019 | 9.16 | 0.955115637028271 | 1.18138267021763 | 0.808472699918922 | -0.30672903653358 | 0.0459541830339333 | 1.33767495030899 | down |
| B4DIP7 | cDNA FLJ57114, highly similar to Niemann-Pick C1 protein OS=Homo sapiens OX=9606 PE=2 SV=1 - [B4DIP7_HUMAN] | 100.62 | 3.48 | 1.08618078737412 | 0.780046787066297 | 1.3924559467249 | 0.477631685592693 | 0.0185734924547825 | 1.73110642629627 | up |
| O75569 | Interferon-inducible double-stranded RNA-dependent protein kinase activator A OS=Homo sapiens OX=9606 GN=PRKRA PE=1 SV=1 - [PRKRA_HUMAN] | 100.399435798658 | 6.39 | 0.920352123209783 | 1.1971082116541 | 0.768812805935136 | -0.379295727941611 | 0.00633682033349913 | 2.19812860615803 | down |
| O75380 | NADH dehydrogenase [ubiquinone] iron-sulfur protein 6, mitochondrial OS=Homo sapiens OX=9606 GN=NDUFS6 PE=1 SV=1 - [NDUS6_HUMAN] | 99.92 | 25.81 | 0.946449431089973 | 1.42365053802516 | 0.664804603244036 | -0.588997723226833 | 0.0010412439980188 | 2.98244748895594 | down |
| P98179 | RNA-binding protein 3 OS=Homo sapiens OX=9606 GN=RBM3 PE=1 SV=1 - [RBM3_HUMAN] | 95.5 | 12.74 | 1.19143459443115 | 0.850073255402478 | 1.40156696715161 | 0.487040678276502 | 0.0296604399223412 | 1.5278224118232 | up |
| B1AUU8 | Epidermal growth factor receptor substrate 15 OS=Homo sapiens OX=9606 GN=EPS15 PE=1 SV=1 - [B1AUU8_HUMAN] | 95.2435692695828 | 4.46 | 0.933864831090595 | 1.14570236019021 | 0.815102476471771 | -0.294946645355042 | 0.0144898068577502 | 1.83893740342961 | down |
| Q9Y3C8 | Ubiquitin-fold modifier-conjugating enzyme 1 OS=Homo sapiens OX=9606 GN=UFC1 PE=1 SV=3 - [UFC1_HUMAN] | 93.8433948277528 | 14.97 | 0.910758339326443 | 1.30191441212432 | 0.699553158675284 | -0.515494403697112 | 0.0306570055725879 | 1.51347026718585 | down |
| B4E0N1 | cDNA FLJ58735, highly similar to Helicase SKI2W OS=Homo sapiens OX=9606 PE=2 SV=1 - [B4E0N1_HUMAN] | 93.6951134633557 | 2.56 | 0.944995251533806 | 1.14219610958444 | 0.827349387381142 | -0.27343139064719 | 0.0392041008391388 | 1.40666850240151 | down |
| B3VL86 | Mutant beta-globin OS=Homo sapiens OX=9606 GN=HBB PE=3 SV=1 - [B3VL86_HUMAN] | 93.5 | 10.07 | 1.0693185462594 | 0.664534201244503 | 1.60912492428055 | 0.68627633391958 | 0.0148394888055869 | 1.82858105948399 | up |
| B3KPA1 | Torsin-1A OS=Homo sapiens OX=9606 PE=2 SV=1 - [B3KPA1_HUMAN] | 91.82 | 4.32 | 1.09006368291939 | 0.881188223201284 | 1.23703841497028 | 0.30689030243763 | 0.0416019193340526 | 1.38088663242712 | up |
| Q96IR7 | 4-hydroxyphenylpyruvate dioxygenase-like protein OS=Homo sapiens OX=9606 GN=HPDL PE=1 SV=1 - [HPDL_HUMAN] | 88.27 | 7.82 | 0.927076714751848 | 1.24898583608244 | 0.742263593364444 | -0.429996485793116 | 0.00503196342439511 | 2.29826252428797 | down |
| D6R9X9 | Ribosomal protein L37 OS=Homo sapiens OX=9606 GN=RPL37 PE=1 SV=1 - [D6R9X9_HUMAN] | 87.6103037568018 | 47.3 | 1.18036943424752 | 0.523812596663798 | 2.2534193369258 | 1.17211580847354 | 0.00134122916211876 | 2.87249701234912 | up |
| C9J6F3 | Programmed cell death protein 10 OS=Homo sapiens OX=9606 GN=PDCD10 PE=1 SV=1 - [C9J6F3_HUMAN] | 86.6971954742699 | 22.04 | 0.946086395569139 | 1.17639724232483 | 0.80422357476753 | -0.314331467401534 | 0.0367617262093385 | 1.43460410377154 | down |
| A0A7I2YQB5 | Procathepsin L OS=Homo sapiens OX=9606 GN=CTSL PE=1 SV=1 - [A0A7I2YQB5_HUMAN] | 86.56 | 5.33 | 1.14878785834297 | 0.681504308131315 | 1.68566485147093 | 0.753317724189385 | 0.000177421024163042 | 3.75099491811767 | up |
| A0A2R8Y484 | Leukocyte surface antigen CD47 (Fragment) OS=Homo sapiens OX=9606 GN=CD47 PE=1 SV=1 - [A0A2R8Y484_HUMAN] | 84.0358047964754 | 9.29 | 1.05513513869101 | 0.836877397656869 | 1.26080013828218 | 0.334339598139001 | 0.047408253361132 | 1.32414604488344 | up |
| Q5TZA2 | Rootletin OS=Homo sapiens OX=9606 GN=CROCC PE=1 SV=2 - [CROCC_HUMAN] | 83.89 | 2.53 | 0.908718457773625 | 1.13248662447778 | 0.80240988116982 | -0.317588722951571 | 0.0418790298115856 | 1.37800338793769 | down |
| P00374 | Dihydrofolate reductase OS=Homo sapiens OX=9606 GN=DHFR PE=1 SV=2 - [DYR_HUMAN] | 82.6737769619538 | 25.67 | 0.963121412450854 | 1.26660533894947 | 0.760395825624479 | -0.395177482900099 | 0.0469266820538203 | 1.32858015144822 | down |
| Q15427 | Splicing factor 3B subunit 4 OS=Homo sapiens OX=9606 GN=SF3B4 PE=1 SV=1 - [SF3B4_HUMAN] | 82.48 | 7.78 | 1.04506332778183 | 0.827276561891781 | 1.2632575077336 | 0.337148754139796 | 0.00571339078279142 | 2.24310607017965 | up |
| P36507 | Dual specificity mitogen-activated protein kinase kinase 2 OS=Homo sapiens OX=9606 GN=MAP2K2 PE=1 SV=1 - [MP2K2_HUMAN] | 81.26 | 8.75 | 0.891031110082097 | 1.5476139686992 | 0.575745068281482 | -0.796497946657034 | 0.0216324518219319 | 1.66489425483782 | down |
| D3DSW1 | STING ER exit protein OS=Homo sapiens OX=9606 GN=hCG_1640171 PE=3 SV=1 - [D3DSW1_HUMAN] | 80.3724964090193 | 6.31 | 0.947106460598314 | 1.17937186752473 | 0.803060075178922 | -0.316420178227179 | 0.0109884202812683 | 1.95906473818986 | down |
| R4GMM1 | MICOS10-NBL1 readthrough (Fragment) OS=Homo sapiens OX=9606 GN=MICOS10-NBL1 PE=4 SV=1 - [R4GMM1_HUMAN] | 79.8766666666667 | 17.39 | 1.06139277348191 | 0.793541982738998 | 1.33753827342366 | 0.419580174570607 | 0.0227087933513619 | 1.64380594172776 | up |
| Q15059 | Bromodomain-containing protein 3 OS=Homo sapiens OX=9606 GN=BRD3 PE=1 SV=1 - [BRD3_HUMAN] | 79.4897922383923 | 6.06 | 1.08595893078415 | 0.885461169881499 | 1.22643314887482 | 0.294468596785614 | 0.0216912551399434 | 1.66371531729976 | up |
| O75394 | 39S ribosomal protein L33, mitochondrial OS=Homo sapiens OX=9606 GN=MRPL33 PE=1 SV=1 - [RM33_HUMAN] | 78.4133333333333 | 26.15 | 1.09307028493705 | 0.822116508327952 | 1.32958075146814 | 0.410971401268379 | 0.0369322100689261 | 1.43259470285484 | up |
| A0A024RDE8 | PDZ and LIM domain 5, isoform CRA_c OS=Homo sapiens OX=9606 GN=PDLIM5 PE=4 SV=1 - [A0A024RDE8_HUMAN] | 77.3870618057893 | 6.38 | 0.957194351345194 | 1.24762507257439 | 0.767213141500987 | -0.382300662571038 | 0.0151715964268757 | 1.8189687182967 | down |
| Q9H3Z4 | DnaJ homolog subfamily C member 5 OS=Homo sapiens OX=9606 GN=DNAJC5 PE=1 SV=1 - [DNJC5_HUMAN] | 76.96 | 12.12 | 1.07964039461468 | 0.817583118553901 | 1.32052676983387 | 0.401113548241169 | 0.0240394108781315 | 1.61907617957744 | up |
| H3BTL1 | Microtubule-associated protein 1 light chain 3 beta, isoform CRA_f OS=Homo sapiens OX=9606 GN=MAP1LC3B PE=3 SV=1 - [H3BTL1_HUMAN] | 76.286604280471 | 34.67 | 1.16940804336824 | 0.54935893705312 | 2.12867756305413 | 1.08995743714954 | 0.000392662209818716 | 3.40598089362883 | up |
| P17252 | Protein kinase C alpha type OS=Homo sapiens OX=9606 GN=PRKCA PE=1 SV=4 - [KPCA_HUMAN] | 74.13 | 6.85 | 0.968380329919876 | 1.17625082762476 | 0.823277065721908 | -0.280550057831803 | 0.0326354938060894 | 1.48630981162934 | down |
| I3L4I0 | FLYWCH family member 2 (Fragment) OS=Homo sapiens OX=9606 GN=FLYWCH2 PE=1 SV=1 - [I3L4I0_HUMAN] | 74.0925472845303 | 8.79 | 1.11352395895417 | 0.611455891615405 | 1.82110267350986 | 0.864812263549103 | 0.0197275454915707 | 1.70492694646492 | up |
| P21912 | Succinate dehydrogenase [ubiquinone] iron-sulfur subunit, mitochondrial OS=Homo sapiens OX=9606 GN=SDHB PE=1 SV=3 - [SDHB_HUMAN] | 74.02 | 16.79 | 0.82566001655645 | 1.42363983818657 | 0.579964113401165 | -0.785964461922911 | 0.00794723664268937 | 2.0997838549227 | down |
| B3KS49 | Gelsolin OS=Homo sapiens OX=9606 PE=2 SV=1 - [B3KS49_HUMAN] | 73.37 | 5.15 | 0.950899922756861 | 1.16429898113199 | 0.816714553707112 | -0.292096158386776 | 0.0141472448008795 | 1.84932813146717 | down |
| M0R3D4 | PRA1 family protein OS=Homo sapiens OX=9606 GN=RABAC1 PE=1 SV=1 - [M0R3D4_HUMAN] | 72.94 | 16.56 | 1.06873831723731 | 0.876106624905428 | 1.21987242974298 | 0.286730283361735 | 0.039208949117563 | 1.40661479755084 | up |
| E9PS95 | Mitochondrial glutamate carrier 1 (Fragment) OS=Homo sapiens OX=9606 GN=SLC25A22 PE=1 SV=1 - [E9PS95_HUMAN] | 72.83 | 20.92 | 1.06904128695356 | 0.826563500866278 | 1.29335651263715 | 0.371120007645119 | 0.0424114289927741 | 1.37251709435894 | up |
| Q8WX93 | Palladin OS=Homo sapiens OX=9606 GN=PALLD PE=1 SV=3 - [PALLD_HUMAN] | 72.78173925807 | 4.19 | 0.92312269039486 | 1.21294212108355 | 0.761060791235623 | -0.393916398436681 | 0.0251510481889126 | 1.59944391068063 | down |
| P30154 | Serine/threonine-protein phosphatase 2A 65 kDa regulatory subunit A beta isoform OS=Homo sapiens OX=9606 GN=PPP2R1B PE=1 SV=3 - [2AAB_HUMAN] | 72.704378088854 | 8.49 | 0.932664747798026 | 1.18565101019987 | 0.786626705307491 | -0.346248929505744 | 0.0130858237590433 | 1.88319893311227 | down |
| A0A7I2V4X2 | Glycogen synthase kinase-3 beta (Fragment) OS=Homo sapiens OX=9606 GN=GSK3B PE=1 SV=1 - [A0A7I2V4X2_HUMAN] | 72.41 | 45.07 | 0.981179098690637 | 1.30516779726731 | 0.751764716188198 | -0.41164689030035 | 0.00562585905555832 | 2.24981115239834 | down |
| K7EIF9 | Signal transducer and activator of transcription OS=Homo sapiens OX=9606 GN=STAT5A PE=1 SV=3 - [K7EIF9_HUMAN] | 71.36 | 7.45 | 0.947701410122249 | 1.15659951007785 | 0.819385968837615 | -0.287384906562323 | 0.00942634020242958 | 2.02565689033792 | down |
| Q15651 | High mobility group nucleosome-binding domain-containing protein 3 OS=Homo sapiens OX=9606 GN=HMGN3 PE=1 SV=2 - [HMGN3_HUMAN] | 71.305 | 31.31 | 1.17551878742144 | 0.687850434618408 | 1.70897440527688 | 0.773130790427376 | 0.00137827572040144 | 2.86066389427827 | up |
| H0YCG9 | Protein wntless homolog (Fragment) OS=Homo sapiens OX=9606 GN=WLS PE=1 SV=1 - [H0YCG9_HUMAN] | 71.1614921118991 | 10.5 | 0.873012749120042 | 1.23194357818344 | 0.708646698258163 | -0.496861555869733 | 0.0162506423672204 | 1.78912946723739 | down |
| P36969 | Phospholipid hydroperoxide glutathione peroxidase OS=Homo sapiens OX=9606 GN=GPX4 PE=1 SV=3 - [GPX4_HUMAN] | 70.0146550639909 | 18.27 | 0.828704926686092 | 1.44895546681259 | 0.571932640903779 | -0.806082850604739 | 0.000787091585039065 | 3.10397473071219 | down |
| Q14525 | Keratin, type I cuticular Ha3-II OS=Homo sapiens OX=9606 GN=KRT33B PE=1 SV=3 - [KT33B_HUMAN] | 69.4188888888889 | 3.47 | 0.867463684537727 | 1.30585516085142 | 0.664287824977572 | -0.59011962184578 | 0.0212596891934793 | 1.67244308894459 | down |
| Q5ZPJ1 | Bax protein (Fragment) OS=Homo sapiens OX=9606 GN=BAX PE=2 SV=1 - [Q5ZPJ1_HUMAN] | 69.03 | 9.82 | 1.1182524487044 | 0.800977909166572 | 1.39610897617383 | 0.481411558488366 | 0.0222185573085741 | 1.65328414401201 | up |
| A0A087WZ06 | Non-specific serine/threonine protein kinase OS=Homo sapiens OX=9606 GN=STK3 PE=1 SV=1 - [A0A087WZ06_HUMAN] | 68.35 | 5 | 0.916667675465392 | 1.1977873579216 | 0.765300843595473 | -0.385901104933994 | 0.010896221590515 | 1.96272407333149 | down |
| Q96JZ5 | Transmembrane 9 superfamily member OS=Homo sapiens OX=9606 GN=SMBP PE=2 SV=1 - [Q96JZ5_HUMAN] | 68.28 | 4.59 | 1.04941422979057 | 0.851265705286489 | 1.23276930254978 | 0.301902842557001 | 0.0405310851402078 | 1.39221176872079 | up |
| I7GPQ7 | cDNA FLJ75793 OS=Homo sapiens OX=9606 PE=2 SV=1 - [I7GPQ7_HUMAN] | 67.79 | 4.79 | 1.03559831200216 | 0.846732163192549 | 1.22305299954298 | 0.290486922688769 | 0.0217769065541506 | 1.66200381245347 | up |
| Q9Y512 | Sorting and assembly machinery component 50 homolog OS=Homo sapiens OX=9606 GN=SAMM50 PE=1 SV=3 - [SAM50_HUMAN] | 67.615 | 5.97 | 1.07374290208429 | 0.787684523748584 | 1.36316363938998 | 0.446958759183311 | 0.0460940817717377 | 1.33635483206964 | up |
| Q5TIH2 | Vesicle transport protein OS=Homo sapiens OX=9606 GN=SFT2D2 PE=1 SV=1 - [Q5TIH2_HUMAN] | 67.02 | 9.26 | 1.10956102291268 | 0.837489439851407 | 1.32486568798951 | 0.40584610979418 | 0.0462979738768419 | 1.33443801445421 | up |
| Q05BW9 | PAPSS1 protein (Fragment) OS=Homo sapiens OX=9606 GN=PAPSS1 PE=2 SV=1 - [Q05BW9_HUMAN] | 66.58 | 7.18 | 0.900551162596298 | 1.26687537088544 | 0.710844320832358 | -0.492394459311924 | 0.0268519803647352 | 1.57102367906086 | down |
| A0A494BXC7 | Nucleoside-diphosphate kinase OS=Homo sapiens OX=9606 GN=CMPK1 PE=1 SV=1 - [A0A494BXC7_HUMAN] | 66.49 | 10.06 | 1.03098809938051 | 0.857529381321721 | 1.20227728849527 | 0.265769671923754 | 0.0261408275160199 | 1.58268066847878 | up |
| Q86W50 | RNA N6-adenosine-methyltransferase METTL16 OS=Homo sapiens OX=9606 GN=METTL16 PE=1 SV=2 - [MET16_HUMAN] | 65.1675749056068 | 3.56 | 0.935958649664241 | 1.26418278731168 | 0.740366550674672 | -0.433688378319976 | 0.0270681308040989 | 1.56754173349782 | down |
| G3V235 | Cyclin-K (Fragment) OS=Homo sapiens OX=9606 GN=CCNK PE=1 SV=8 - [G3V235_HUMAN] | 64.88 | 7.34 | 1.06952535020191 | 0.738566164570269 | 1.44811040839409 | 0.534171602124339 | 6.39904373261083e-05 | 4.19388492174736 | up |
| H0Y612 | E3 ubiquitin-protein ligase TRIM33 (Fragment) OS=Homo sapiens OX=9606 GN=TRIM33 PE=1 SV=1 - [H0Y612_HUMAN] | 63.8 | 1.01 | 0.954495586628584 | 1.22498152870659 | 0.779191819844334 | -0.359949563170342 | 0.0446035363891828 | 1.3506307069062 | down |
| B2RDP6 | cDNA, FLJ96709, highly similar to Homo sapiens glutamate rich WD repeat protein GRWD (GRWD), mRNA OS=Homo sapiens OX=9606 PE=2 SV=1 - [B2RDP6_HUMAN] | 63.73 | 7.4 | 0.918317495399777 | 1.19501971785406 | 0.768453843630993 | -0.379969486323883 | 0.000324637428074397 | 3.48860141097584 | down |
| F8W7Q4 | Protein FAM162A OS=Homo sapiens OX=9606 GN=FAM162A PE=1 SV=1 - [F8W7Q4_HUMAN] | 62.8166666666667 | 25 | 1.06726283188239 | 0.792713191384547 | 1.34634170779765 | 0.429044619145466 | 0.0329013730091389 | 1.4827859781032 | up |
| E7EX90 | Dynactin subunit 1 OS=Homo sapiens OX=9606 GN=DCTN1 PE=1 SV=1 - [E7EX90_HUMAN] | 62.7212054460077 | 6.37 | 0.893341533351002 | 1.11660104324411 | 0.800054360289275 | -0.321830066568156 | 0.019952472450723 | 1.70000328016903 | down |
| B4DNH6 | Perilipin OS=Homo sapiens OX=9606 PE=2 SV=1 - [B4DNH6_HUMAN] | 60.88 | 2.57 | 0.934242813683878 | 1.31893596031005 | 0.708330686096587 | -0.497505051229587 | 0.0141113954868119 | 1.85043003640479 | down |
| A0A0S2Z5P5 | Mini-chromosome maintenance complex-binding protein (Fragment) OS=Homo sapiens OX=9606 GN=MCMBP PE=2 SV=1 - [A0A0S2Z5P5_HUMAN] | 60.69 | 1.56 | 0.877986984638043 | 1.28954572087309 | 0.680849829848296 | -0.554591466435381 | 0.0299083598886449 | 1.52420740210881 | down |
| S4R3Z2 | Aldo-keto reductase family 1 member C3 OS=Homo sapiens OX=9606 GN=AKR1C3 PE=1 SV=1 - [S4R3Z2_HUMAN] | 59.4 | 14 | 0.930570126220375 | 1.20677538999974 | 0.771121232610299 | -0.374970402144876 | 0.0247952540494955 | 1.60563143761571 | down |
| O75116 | Rho-associated protein kinase 2 OS=Homo sapiens OX=9606 GN=ROCK2 PE=1 SV=4 - [ROCK2_HUMAN] | 59.2570275614304 | 5.55 | 0.937907220896519 | 1.21720312692026 | 0.770542894734085 | -0.376052823679259 | 0.0425073939977073 | 1.37153551952377 | down |
| P08243 | Asparagine synthetase [glutamine-hydrolyzing] OS=Homo sapiens OX=9606 GN=ASNS PE=1 SV=4 - [ASNS_HUMAN] | 57.7875463741376 | 5.88 | 0.943974870030653 | 1.15232575594923 | 0.819190984109397 | -0.287728257542905 | 0.0368758520627163 | 1.43325793598027 | down |
| Q9BSD7 | Cancer-related nucleoside-triphosphatase OS=Homo sapiens OX=9606 GN=NTPCR PE=1 SV=1 - [NTPCR_HUMAN] | 57.48 | 5.79 | 0.937001730145382 | 1.1890324038911 | 0.78803716961712 | -0.343664415513313 | 0.00917314479393098 | 2.03748175129684 | down |
| E9PI38 | Transcription factor p65 (Fragment) OS=Homo sapiens OX=9606 GN=RELA PE=1 SV=1 - [E9PI38_HUMAN] | 56.93 | 9.19 | 0.93527212116713 | 1.24892863081728 | 0.7488595409612 | -0.417232948381538 | 0.0366363770942841 | 1.43608747948437 | down |
| H3BQF7 | IST1 homolog (Fragment) OS=Homo sapiens OX=9606 GN=IST1 PE=1 SV=1 - [H3BQF7_HUMAN] | 56.19 | 12.65 | 1.05701679325876 | 0.875804932197719 | 1.20690892960184 | 0.271316817958106 | 0.0336921794747163 | 1.47247089453305 | up |
| A3KMH1 | von Willebrand factor A domain-containing protein 8 OS=Homo sapiens OX=9606 GN=VWA8 PE=1 SV=2 - [VWA8_HUMAN] | 55.83 | 0.79 | 1.03481845704489 | 0.753252871358761 | 1.37379955177366 | 0.458171518887962 | 0.0064685169161568 | 2.18919528176744 | up |
| B4E2S7 | cDNA FLJ58780, highly similar to Homo sapiens lysosomal-associated membrane protein 2 (LAMP2), transcript variant LAMP2B, mRNA OS=Homo sapiens OX=9606 PE=2 SV=1 - [B4E2S7_HUMAN] | 55.6603059129143 | 5.51 | 1.03182113840032 | 0.777863514231486 | 1.32648095652067 | 0.407603963821116 | 0.0147351426221133 | 1.83164565623774 | up |
| A8K2X6 | cDNA FLJ77840, highly similar to Homo sapiens solute carrier family 43, member 3 (SLC43A3), mRNA OS=Homo sapiens OX=9606 PE=2 SV=1 - [A8K2X6_HUMAN] | 54.7555728776693 | 9.16 | 1.05197028195269 | 0.867235576865736 | 1.21301559808536 | 0.278598102095448 | 0.0306339427658448 | 1.51379710338108 | up |
| B1AJQ6 | Syntaxin-12 (Fragment) OS=Homo sapiens OX=9606 GN=STX12 PE=1 SV=2 - [B1AJQ6_HUMAN] | 52.98 | 6.98 | 1.06771816926755 | 0.848829936960413 | 1.25787053775572 | 0.330983445151184 | 0.0144832854923465 | 1.83913290848809 | up |
| T2C6S4 | WWC family member 3 OS=Homo sapiens OX=9606 GN=WWC3 PE=2 SV=1 - [T2C6S4_HUMAN] | 52.7714474570745 | 1.81 | 1.09986974525102 | 0.749203741499152 | 1.4680515917475 | 0.553902669714091 | 0.00947854076047156 | 2.02325851798421 | up |
| J3QR51 | Ras-related protein Rab-31 OS=Homo sapiens OX=9606 GN=RAB31 PE=1 SV=2 - [J3QR51_HUMAN] | 52.36 | 14.86 | 1.09883579992921 | 0.812053313160863 | 1.35315721532132 | 0.436329467243378 | 0.0281006470532326 | 1.55128367979552 | up |
| E9PMH2 | Peptidylprolyl isomerase OS=Homo sapiens OX=9606 GN=AIP PE=1 SV=3 - [E9PMH2_HUMAN] | 50.3866666666667 | 11.61 | 0.918561781973657 | 1.22099560367611 | 0.75230556048531 | -0.410609341149798 | 0.0425212398378775 | 1.37139408076919 | down |
| H6UMI1 | GABARAP-a OS=Homo sapiens OX=9606 GN=GABARAP PE=1 SV=1 - [H6UMI1_HUMAN] | 50.2682401120805 | 24.49 | 1.08462736254716 | 0.780552865399689 | 1.38956297597059 | 0.474631219973257 | 0.00329755335103257 | 2.48180816928528 | up |
| Q9BVV7 | Mitochondrial import inner membrane translocase subunit Tim21 OS=Homo sapiens OX=9606 GN=TIMM21 PE=1 SV=1 - [TIM21_HUMAN] | 50.11 | 3.23 | 1.06832057551272 | 0.827314072696168 | 1.29131198268044 | 0.368837599785043 | 0.0126705977802911 | 1.89720289525408 | up |
| C9JER0 | POM121 and ZP3 fusion protein OS=Homo sapiens OX=9606 GN=POMZP3 PE=4 SV=1 - [C9JER0_HUMAN] | 49.78 | 5.19 | 1.0818146061438 | 0.871965992412752 | 1.24066146565005 | 0.311109506720553 | 0.0435540811341589 | 1.36097114419242 | up |
| B7ZL13 | FNBP1 protein OS=Homo sapiens OX=9606 GN=FNBP1 PE=2 SV=1 - [B7ZL13_HUMAN] | 48.57 | 3.79 | 0.939348860196387 | 1.16125759981398 | 0.808906534042799 | -0.305955080319781 | 0.0210107652980982 | 1.67755812852275 | down |
| K7EK42 | Tubulin-folding cofactor B OS=Homo sapiens OX=9606 GN=TBCB PE=1 SV=1 - [K7EK42_HUMAN] | 48.44 | 9.42 | 0.938912908295326 | 1.1888739393631 | 0.789749760010987 | -0.340532501321054 | 0.0261918337273206 | 1.58183409493948 | down |
| C9J470 | Condensin complex subunit 2 (Fragment) OS=Homo sapiens OX=9606 GN=NCAPH PE=1 SV=8 - [C9J470_HUMAN] | 47.8739662347008 | 4.28 | 0.935046357798269 | 1.12699573383769 | 0.829680476796669 | -0.269372256372174 | 0.0276266960229446 | 1.55867105083457 | down |
| A0A7L7TUW1 | MHC class I antigen (Fragment) OS=Homo sapiens OX=9606 GN=HLA-C PE=3 SV=1 - [A0A7L7TUW1_HUMAN] | 46.85 | 8.88 | 0.948923148778141 | 1.21801822702384 | 0.77907138639196 | -0.360172566247289 | 0.0402470574634905 | 1.39526586620502 | down |
| A0A7P0T9T8 | RNA helicase OS=Homo sapiens OX=9606 GN=DDX20 PE=1 SV=1 - [A0A7P0T9T8_HUMAN] | 46.67 | 2.79 | 0.920193984630581 | 1.32255009867155 | 0.695772497053139 | -0.523312442588444 | 0.0191247288993971 | 1.71840471242691 | down |
| B4DPU0 | Phospholipase A2 group XV OS=Homo sapiens OX=9606 GN=PLA2G15 PE=1 SV=1 - [B4DPU0_HUMAN] | 45.55 | 3.77 | 0.840691708597772 | 1.31814576683713 | 0.637783566695356 | -0.648861169690649 | 0.00441839395640697 | 2.35473556382315 | down |
| B4E324 | cDNA FLJ60397, highly similar to Lysosomal protective protein OS=Homo sapiens OX=9606 PE=2 SV=1 - [B4E324_HUMAN] | 44.3939770124401 | 4.17 | 0.907766310224161 | 1.28265562127655 | 0.707724111730561 | -0.498741023007887 | 0.00221064718769474 | 2.65548056391778 | down |
| Q86VV8 | Rotatin OS=Homo sapiens OX=9606 GN=RTTN PE=1 SV=3 - [RTTN_HUMAN] | 44.3120400420209 | 1.57 | 0.9630661225699 | 1.21939838236459 | 0.789787928619665 | -0.340462777549516 | 0.0126671235104677 | 1.89732199485306 | down |
| P38432 | Coilin OS=Homo sapiens OX=9606 GN=COIL PE=1 SV=1 - [COIL_HUMAN] | 43.87 | 2.78 | 1.05667166888244 | 0.722041556905693 | 1.46344993411571 | 0.549373390749407 | 0.0255327187107904 | 1.59290293927994 | up |
| Q8NHP8 | Putative phospholipase B-like 2 OS=Homo sapiens OX=9606 GN=PLBD2 PE=1 SV=2 - [PLBL2_HUMAN] | 43.5491581442942 | 1.19 | 0.821096409728597 | 1.29223206685829 | 0.635409405777142 | -0.654241648890719 | 0.00588575285255706 | 2.23019797822421 | down |
| Q86TW5 | Full-length cDNA clone CS0DC006YI13 of Neuroblastoma of Homo sapiens (human) (Fragment) OS=Homo sapiens OX=9606 PE=2 SV=1 - [Q86TW5_HUMAN] | 43.2762204380993 | 13.67 | 1.01792364900804 | 0.674202233903946 | 1.50981945448888 | 0.594376041148585 | 0.0328303561367747 | 1.48372440611022 | up |
| A0A0A0MTR2 | Prolyl 3-hydroxylase OGFOD1 OS=Homo sapiens OX=9606 GN=OGFOD1 PE=1 SV=1 - [A0A0A0MTR2_HUMAN] | 43.2 | 4.61 | 0.916838279189717 | 1.32940166740012 | 0.689662350870037 | -0.536037883649687 | 0.0432795050644355 | 1.36371771437592 | down |
| P23258 | Tubulin gamma-1 chain OS=Homo sapiens OX=9606 GN=TUBG1 PE=1 SV=2 - [TBG1_HUMAN] | 42.43 | 3.1 | 0.898957330646315 | 1.19005487720833 | 0.755391493168045 | -0.40470355791015 | 0.00388795735346283 | 2.41027850744364 | down |
| B2R8X4 | cDNA, FLJ94105, highly similar to Homo sapiens GA binding protein transcription factor, alpha subunit 60kDa (GABPA), mRNA OS=Homo sapiens OX=9606 PE=2 SV=1 - [B2R8X4_HUMAN] | 42.17 | 3.96 | 0.919179690741343 | 1.24958573163057 | 0.73558753711273 | -0.443031058174455 | 0.0478539398909313 | 1.32008230026015 | down |
| P09417 | Dihydropteridine reductase OS=Homo sapiens OX=9606 GN=QDPR PE=1 SV=2 - [DHPR_HUMAN] | 41.99 | 3.28 | 0.984940376687419 | 1.20747484151678 | 0.815702607476426 | -0.293884831074594 | 0.0327654820378828 | 1.48458343842499 | down |
| A4FU79 | ARID1A protein (Fragment) OS=Homo sapiens OX=9606 GN=ARID1A PE=2 SV=1 - [A4FU79_HUMAN] | 41.2 | 7.93 | 0.919118715174286 | 1.16638854139126 | 0.788003896264248 | -0.343725331820924 | 0.0311006111437047 | 1.5072310767696 | down |
| B4DWU3 | Palmitoyl-protein thioesterase 1 OS=Homo sapiens OX=9606 PE=2 SV=1 - [B4DWU3_HUMAN] | 41.02 | 6.03 | 0.897124076216491 | 1.3004035952447 | 0.689881264168356 | -0.535580014642752 | 0.00651092175797918 | 2.18635752355681 | down |
| A0A8I5KX20 | Cytochrome b5 type B OS=Homo sapiens OX=9606 GN=CYB5B PE=4 SV=1 - [A0A8I5KX20_HUMAN] | 40.88 | 18.46 | 1.0769851224314 | 0.794391524131056 | 1.35573591826708 | 0.439076185472607 | 0.0287035884178643 | 1.54206380596083 | up |
| Q92896 | Golgi apparatus protein 1 OS=Homo sapiens OX=9606 GN=GLG1 PE=1 SV=2 - [GSLG1_HUMAN] | 40.5719044967415 | 3.31 | 0.836635394241157 | 1.34550663616979 | 0.621799530192416 | -0.685478568252334 | 0.0110681911362821 | 1.95592334964711 | down |
| K7EQ85 | Alanyl-tRNA-editing protein Aarsd1 (Fragment) OS=Homo sapiens OX=9606 GN=AARSD1 PE=1 SV=1 - [K7EQ85_HUMAN] | 39.89 | 13.83 | 0.884824528534292 | 1.19016294746077 | 0.743448223137917 | -0.427695824463398 | 0.0463739109359485 | 1.33372627640788 | down |
| B3KNP0 | cDNA FLJ30074 fis, clone BGGI11000123, highly similar to Homo sapiens formin binding protein 4 (FNBP4), mRNA (Fragment) OS=Homo sapiens OX=9606 PE=2 SV=1 - [B3KNP0_HUMAN] | 39.7 | 0.86 | 1.06698321319238 | 0.752006768074457 | 1.41884788606948 | 0.504719927117582 | 0.00259331407866356 | 2.58614488223944 | up |
| B4DGK8 | cDNA FLJ57723, moderately similar to Protein-tyrosine phosphatase mitochondrial 1, mitochondrial OS=Homo sapiens OX=9606 PE=2 SV=1 - [B4DGK8_HUMAN] | 39.21 | 6.57 | 1.11996007404479 | 0.788672599234143 | 1.42005703650964 | 0.50594887665747 | 0.0267109224386448 | 1.57331111373468 | up |
| Q13637 | Ras-related protein Rab-32 OS=Homo sapiens OX=9606 GN=RAB32 PE=1 SV=3 - [RAB32_HUMAN] | 38.99 | 9.33 | 1.07585927700832 | 0.889112435777809 | 1.21003737403261 | 0.27505160824268 | 0.0321615820287197 | 1.49266259644583 | up |
| H0YE28 | Selenoprotein H (Fragment) OS=Homo sapiens OX=9606 GN=SELENOH PE=1 SV=1 - [H0YE28_HUMAN] | 38.6 | 17.02 | 0.960320937839502 | 1.15870628098791 | 0.828787203104425 | -0.270926367635477 | 0.0120301655397179 | 1.91972839655946 | down |
| K7EQJ5 | 40S ribosomal protein S15 OS=Homo sapiens OX=9606 GN=RPS15 PE=1 SV=2 - [K7EQJ5_HUMAN] | 38.5 | 21.28 | 0.953321949185477 | 1.17390803511196 | 0.812092532524963 | -0.300283972638124 | 0.020891043522534 | 1.68003986615854 | down |
| F8WBV5 | Thioredoxin domain-containing protein 9 OS=Homo sapiens OX=9606 GN=TXNDC9 PE=1 SV=1 - [F8WBV5_HUMAN] | 38.25 | 17.19 | 0.90259602547512 | 1.34235848712207 | 0.672395663404511 | -0.572617674901297 | 0.0439432663526087 | 1.35710766461644 | down |
| Q06203 | Amidophosphoribosyltransferase OS=Homo sapiens OX=9606 GN=PPAT PE=1 SV=1 - [PUR1_HUMAN] | 38.19 | 2.32 | 0.914794085045263 | 1.30095603979151 | 0.70317063533666 | -0.508053270621544 | 0.0144188427663563 | 1.84106959401002 | down |
| B0QYG7 | Protein kinase C and casein kinase substrate in neurons protein 2 (Fragment) OS=Homo sapiens OX=9606 GN=PACSIN2 PE=1 SV=1 - [B0QYG7_HUMAN] | 37.55 | 9.21 | 0.938583075302766 | 1.22265620311335 | 0.767659030324939 | -0.381462440828527 | 0.025926147706295 | 1.58626200903813 | down |
| A0A1W2PPP6 | Elongator complex protein 4 OS=Homo sapiens OX=9606 GN=ELP4 PE=1 SV=1 - [A0A1W2PPP6_HUMAN] | 37.13 | 6.6 | 0.832647083527623 | 1.52937346112642 | 0.544436728302033 | -0.877163698964159 | 0.0290128839372839 | 1.53740909933256 | down |
| Q05CN4 | PPFIBP1 protein (Fragment) OS=Homo sapiens OX=9606 GN=PPFIBP1 PE=2 SV=1 - [Q05CN4_HUMAN] | 36.51 | 6.85 | 1.01791546798764 | 0.843490139496261 | 1.20679000301717 | 0.271174650439399 | 0.0431710355265106 | 1.36480753396927 | up |
| D6RAZ0 | RING-type E3 ubiquitin transferase OS=Homo sapiens OX=9606 GN=MIB2 PE=1 SV=1 - [D6RAZ0_HUMAN] | 36.3 | 0.74 | 1.047024390808 | 0.773150436170763 | 1.35423113255122 | 0.437473991001389 | 0.044205613279244 | 1.35452257994014 | up |
| B4DN40 | cDNA FLJ54368, highly similar to Phosphoglucomutase-2 OS=Homo sapiens OX=9606 PE=2 SV=1 - [B4DN40_HUMAN] | 35.77 | 5.75 | 0.925518131112143 | 1.34405861156239 | 0.688599532156027 | -0.538262894166012 | 0.0491180441671697 | 1.30875893470721 | down |
| B4DUV1 | Fibulin-1 OS=Homo sapiens OX=9606 PE=2 SV=1 - [B4DUV1_HUMAN] | 35.76 | 2.65 | 1.1039097731037 | 0.813976495610327 | 1.35619367273741 | 0.439563218847482 | 0.00124275043815713 | 2.90561607506952 | up |
| Q9UNY4 | Transcription termination factor 2 OS=Homo sapiens OX=9606 GN=TTF2 PE=1 SV=2 - [TTF2_HUMAN] | 35.4524613894425 | 1.89 | 1.07903206773524 | 0.666597046899967 | 1.61871714366771 | 0.69485090961851 | 0.0375508581984334 | 1.42538013305169 | up |
| Q8NCN4 | E3 ubiquitin-protein ligase RNF169 OS=Homo sapiens OX=9606 GN=RNF169 PE=1 SV=2 - [RN169_HUMAN] | 35.42 | 1.13 | 1.04600196747839 | 0.861066213123261 | 1.21477530012975 | 0.280689479783143 | 0.0374464686409653 | 1.42658913182446 | up |
| A0A024R8P7 | Glutamate receptor OS=Homo sapiens OX=9606 GN=GRIN2C PE=3 SV=1 - [A0A024R8P7_HUMAN] | 35.0454329388981 | 2.04 | 1.12330233357195 | 0.710597002679403 | 1.58078676005723 | 0.660642768735777 | 0.0426900648934126 | 1.36967318502202 | up |
| P56937 | 3-keto-steroid reductase/17-beta-hydroxysteroid dehydrogenase 7 OS=Homo sapiens OX=9606 GN=HSD17B7 PE=1 SV=1 - [DHB7_HUMAN] | 34.84 | 3.52 | 1.11657872678914 | 0.839302628155382 | 1.33036486403379 | 0.411821971547812 | 0.00748759465843554 | 2.12565767420178 | up |
| Q9P1X0 | Cystine/glutamate transporter (Fragment) OS=Homo sapiens OX=9606 GN=xCT PE=4 SV=1 - [Q9P1X0_HUMAN] | 34.63 | 9.78 | 1.05504760991921 | 0.829913434561298 | 1.27127428715132 | 0.34627533641753 | 0.0441611598621853 | 1.3549595286862 | up |
| Q9BQB6 | Vitamin K epoxide reductase complex subunit 1 OS=Homo sapiens OX=9606 GN=VKORC1 PE=1 SV=1 - [VKOR1_HUMAN] | 34.21 | 15.34 | 1.02394707048472 | 0.812210129690505 | 1.26069231723926 | 0.334216216541188 | 0.00973588063907072 | 2.01162475915255 | up |
| M0QZR8 | DNA polymerase OS=Homo sapiens OX=9606 GN=POLD1 PE=1 SV=1 - [M0QZR8_HUMAN] | 33.72 | 2.87 | 0.910211685668708 | 1.26460472280614 | 0.719759834242089 | -0.474412499093975 | 0.02301974917386 | 1.63789941280883 | down |
| B4DL53 | cDNA FLJ60290, highly similar to Mus musculus strawberry notch homolog 2, mRNA OS=Homo sapiens OX=9606 PE=2 SV=1 - [B4DL53_HUMAN] | 33.68 | 2.97 | 0.940953076748475 | 1.15022942058938 | 0.818056867530241 | -0.289726958746998 | 0.0401443307880512 | 1.39637577744154 | down |
| C9JNE2 | ADP-ribose glycohydrolase OARD1 (Fragment) OS=Homo sapiens OX=9606 GN=OARD1 PE=1 SV=1 - [C9JNE2_HUMAN] | 33.59 | 9.92 | 0.923299874478807 | 1.26145443905842 | 0.731932795898664 | -0.450216904676765 | 0.0355179241716594 | 1.44955242428808 | down |
| A0A3B3IU56 | Hydroxymethylbilane synthase OS=Homo sapiens OX=9606 GN=HMBS PE=1 SV=1 - [A0A3B3IU56_HUMAN] | 33.11 | 5.3 | 0.973051814264865 | 1.20639316217884 | 0.806579351384467 | -0.310111621993237 | 0.0336277288662318 | 1.4733024628718 | down |
| Q8NBP7 | Proprotein convertase subtilisin/kexin type 9 OS=Homo sapiens OX=9606 GN=PCSK9 PE=1 SV=3 - [PCSK9_HUMAN] | 32.74 | 3.32 | 1.11303068136906 | 0.76651441403278 | 1.45206751626912 | 0.538108535373673 | 0.00138007802645559 | 2.86009635888778 | up |
| B7ZM65 | PHD and RING finger domain-containing protein 1 OS=Homo sapiens OX=9606 GN=PHRF1 PE=1 SV=1 - [B7ZM65_HUMAN] | 32.53 | 1.46 | 0.895376671994696 | 1.30508188150528 | 0.686069345290404 | -0.543573688969793 | 0.00847874286526505 | 2.07166853537906 | down |
| Q8N3C0 | Activating signal cointegrator 1 complex subunit 3 OS=Homo sapiens OX=9606 GN=ASCC3 PE=1 SV=3 - [ASCC3_HUMAN] | 31.91 | 0.68 | 0.90782049358122 | 1.15712935553029 | 0.784545383143601 | -0.350071190533432 | 0.0204839746350849 | 1.68858577061588 | down |
| O43760 | Synaptogyrin-2 OS=Homo sapiens OX=9606 GN=SYNGR2 PE=1 SV=1 - [SNG2_HUMAN] | 31.91 | 8.04 | 1.06335150349986 | 0.847948656720181 | 1.25402817148487 | 0.326569758351275 | 0.0468038367525062 | 1.3297185441023 | up |
| B4E3I5 | cDNA FLJ56216, highly similar to Amyloid-like protein 2 (CDEIbox-binding protein) (CDEBP) OS=Homo sapiens OX=9606 PE=2 SV=1 - [B4E3I5_HUMAN] | 31.76 | 2.9 | 1.16115655229612 | 0.686738146835711 | 1.69082867705309 | 0.757730486307602 | 0.00300076881740797 | 2.52276746181988 | up |
| B4DGC3 | Apolipoprotein D OS=Homo sapiens OX=9606 PE=2 SV=1 - [B4DGC3_HUMAN] | 31.52 | 4.69 | 1.09594941259816 | 0.756344576101059 | 1.44900809396658 | 0.535065653620054 | 0.00642892191388584 | 2.1918618491762 | up |
| A0A3S8NFS6 | ATIII-T1 OS=Homo sapiens OX=9606 PE=3 SV=1 - [A0A3S8NFS6_HUMAN] | 31.5 | 5.07 | 1.10030377408951 | 0.827983366433078 | 1.32889598836941 | 0.410228190441085 | 0.00440294865340766 | 2.35625637919452 | up |
| Q14694 | Ubiquitin carboxyl-terminal hydrolase 10 OS=Homo sapiens OX=9606 GN=USP10 PE=1 SV=2 - [UBP10_HUMAN] | 31.24 | 3.76 | 0.96677073774807 | 1.17193699355755 | 0.824934056235673 | -0.277649297400669 | 0.0452794774232279 | 1.34409859404413 | down |
| A8K6N3 | cDNA FLJ76886, highly similar to Homo sapiens loss of heterozygosity, 11, chromosomal region 2, gene A (LOH11CR2A), transcript variant 1, mRNA OS=Homo sapiens OX=9606 PE=2 SV=1 - [A8K6N3_HUMAN] | 30.97 | 2.67 | 0.908586825599055 | 1.1508585328628 | 0.789486109416868 | -0.341014212021221 | 0.0288421219491859 | 1.53997279121607 | down |
| Q9BRP8 | Partner of Y14 and mago OS=Homo sapiens OX=9606 GN=PYM1 PE=1 SV=1 - [PYM1_HUMAN] | 30.97 | 6.37 | 0.960050178612222 | 1.29554903834703 | 0.741037313290075 | -0.432381906818817 | 0.032591531655625 | 1.48689522916494 | down |
| Q9H089 | Large subunit GTPase 1 homolog OS=Homo sapiens OX=9606 GN=LSG1 PE=1 SV=2 - [LSG1_HUMAN] | 30.12 | 3.5 | 1.053324246938 | 0.862566142004714 | 1.22115185797804 | 0.28824261975937 | 0.0224722550267014 | 1.64835334518149 | up |
| Q9NUM4 | Transmembrane protein 106B OS=Homo sapiens OX=9606 GN=TMEM106B PE=1 SV=2 - [T106B_HUMAN] | 29.93 | 4.74 | 1.09954131257882 | 0.843427818128404 | 1.30365786964288 | 0.382565300142281 | 0.0223099050297401 | 1.65150227844532 | up |
| A8MVI5 | Pre-mRNA-splicing factor ISY1 homolog OS=Homo sapiens OX=9606 GN=ISY1 PE=1 SV=1 - [A8MVI5_HUMAN] | 29.2 | 4.37 | 0.905169085675925 | 1.36882216554413 | 0.661275882624317 | -0.596675808847131 | 0.00292021031125837 | 2.53458586987696 | down |
| E9PS78 | Cathepsin B (Fragment) OS=Homo sapiens OX=9606 GN=CTSB PE=1 SV=1 - [E9PS78_HUMAN] | 28.68 | 8.7 | 0.83940454653581 | 1.44726860412386 | 0.579992230981866 | -0.785894519473019 | 0.00419910648492083 | 2.37684311197146 | down |
| F8WBP3 | Sperm-associated antigen 8 OS=Homo sapiens OX=9606 GN=SPAG8 PE=1 SV=1 - [F8WBP3_HUMAN] | 27.52 | 7.29 | 0.947574002932438 | 1.22416710909073 | 0.774056087519182 | -0.36948998813769 | 0.0357513725496648 | 1.44670728031596 | down |
| B3KPI5 | cDNA FLJ31840 fis, clone NT2RP7000109, highly similar to Tudor and KH domain-containing protein OS=Homo sapiens OX=9606 PE=2 SV=1 - [B3KPI5_HUMAN] | 27.31 | 1.08 | 1.09047403213304 | 0.87010613130693 | 1.25326554186569 | 0.325692125230197 | 0.032820866216714 | 1.48384996112038 | up |
| A0A1B0GW05 | Probable C-mannosyltransferase DPY19L1 OS=Homo sapiens OX=9606 GN=DPY19L1 PE=1 SV=1 - [A0A1B0GW05_HUMAN] | 27.08 | 1.87 | 1.05241332169573 | 0.728417589313598 | 1.4447939439346 | 0.530863750676539 | 0.000664388450575128 | 3.17757792573161 | up |
| P54851 | Epithelial membrane protein 2 OS=Homo sapiens OX=9606 GN=EMP2 PE=1 SV=1 - [EMP2_HUMAN] | 26.74 | 7.19 | 1.05919149131395 | 0.766717618127536 | 1.38146230929281 | 0.466196201355531 | 0.0175186684841201 | 1.75649890570073 | up |
| H7C3J6 | Sterile alpha motif domain-containing protein 11 (Fragment) OS=Homo sapiens OX=9606 GN=SAMD11 PE=1 SV=1 - [H7C3J6_HUMAN] | 26.2 | 1.29 | 1.05510544698722 | 0.547394642190048 | 1.92750415452715 | 0.946733969717095 | 0.0409794786835928 | 1.38743357073422 | up |
| Q9BS19 | Epididymis secretory sperm binding protein OS=Homo sapiens OX=9606 PE=1 SV=1 - [Q9BS19_HUMAN] | 26.06 | 4.33 | 0.761318350930771 | 1.64968023050975 | 0.46149449866143 | -1.11561464485202 | 0.0074851204098284 | 2.12580120896109 | down |
| A0A0A0MTS7 | Titin OS=Homo sapiens OX=9606 GN=TTN PE=1 SV=1 - [A0A0A0MTS7_HUMAN] | 26.04 | 1.15 | 0.900085128475354 | 1.1907662430125 | 0.755887340405489 | -0.403756867782193 | 0.0483202947770796 | 1.31587042497325 | down |
| A0A6Q8PF00 | DNA-binding protein SMUBP-2 (Fragment) OS=Homo sapiens OX=9606 GN=IGHMBP2 PE=1 SV=1 - [A0A6Q8PF00_HUMAN] | 25.43 | 12.96 | 0.940067827099285 | 1.16480537062664 | 0.807060004018995 | -0.309252154615461 | 0.0181060703330016 | 1.74217579694602 | down |
| R4GN18 | Membrane cofactor protein (Fragment) OS=Homo sapiens OX=9606 GN=CD46 PE=1 SV=1 - [R4GN18_HUMAN] | 25.26 | 10.26 | 1.14852477219121 | 0.811050413882684 | 1.41609541470173 | 0.501918475646489 | 0.0360089345455601 | 1.4435897286101 | up |
| Q8N516 | Elongator complex protein 1 OS=Homo sapiens OX=9606 GN=IKBKAP PE=2 SV=1 - [Q8N516_HUMAN] | 22.59 | 1.5 | 1.14181719605522 | 0.725762032335324 | 1.57326664276049 | 0.653763204502861 | 0.0358248352154609 | 1.44581579856302 | up |
| A5PKX9 | INADL protein OS=Homo sapiens OX=9606 GN=INADL PE=2 SV=1 - [A5PKX9_HUMAN] | 22.11 | 1.02 | 1.0906117260991 | 0.773593536326865 | 1.40979942939736 | 0.495489926598238 | 0.0420504948399034 | 1.37622888916512 | up |
| H7C2K6 | Band 4.1-like protein 1 (Fragment) OS=Homo sapiens OX=9606 GN=EPB41L1 PE=1 SV=1 - [H7C2K6_HUMAN] | 20.9 | 10.07 | 1.20418874086053 | 0.738098341180719 | 1.63147466086188 | 0.706176580483539 | 0.0162950217262066 | 1.78794505615218 | up |
| S4R360 | 39S ribosomal protein L42, mitochondrial OS=Homo sapiens OX=9606 GN=MRPL42 PE=1 SV=1 - [S4R360_HUMAN] | 20.15 | 4.69 | 1.03766232696423 | 0.785931993824966 | 1.32029531195714 | 0.400860654921256 | 0.00140470367315047 | 2.85241528194307 | up |
| Q9Y6X1 | Stress-associated endoplasmic reticulum protein 1 OS=Homo sapiens OX=9606 GN=SERP1 PE=1 SV=1 - [SERP1_HUMAN] | 19.26 | 24.24 | 1.05317506713915 | 0.853283932451552 | 1.23426098521894 | 0.303647485468207 | 0.0381647059956558 | 1.41833807888585 | up |
| B4DHG3 | Procollagen-lysine 5-dioxygenase OS=Homo sapiens OX=9606 PE=2 SV=1 - [B4DHG3_HUMAN] | 17.84 | 0.71 | 1.08679228362105 | 0.847958534130097 | 1.28165734511531 | 0.358010604718357 | 0.030837889858486 | 1.51091534703382 | up |
| Q5RI15 | Cytochrome c oxidase assembly protein COX20, mitochondrial OS=Homo sapiens OX=9606 GN=COX20 PE=1 SV=2 - [COX20_HUMAN] | 14.17 | 8.47 | 1.03319242632332 | 0.804223681526745 | 1.28470778721897 | 0.361440249013771 | 0.0485976692407229 | 1.31338455913618 | up |
| J3KNV1 | Zinc finger protein 292 OS=Homo sapiens OX=9606 GN=ZNF292 PE=1 SV=1 - [J3KNV1_HUMAN] | 0 | 0.92 | 1.11431765404321 | 0.817980913174094 | 1.36227830759425 | 0.446021469847479 | 0.0387312886135242 | 1.41193805353204 | up |
| Q8WXH0 | Nesprin-2 OS=Homo sapiens OX=9606 GN=SYNE2 PE=1 SV=3 - [SYNE2_HUMAN] | 0 | 1.03 | 1.10497619130887 | 0.732981011009183 | 1.50750998281321 | 0.592167555762131 | 0.0336400366510842 | 1.47314353970662 | up |
| Q96T58 | Msx2-interacting protein OS=Homo sapiens OX=9606 GN=SPEN PE=1 SV=1 - [MINT_HUMAN] | 0 | 1.15 | 1.0138956102222 | 0.538536600524866 | 1.88268654207353 | 0.912792818411785 | 0.038497246911792 | 1.41457032747251 | up |
| Q5JXX6 | Mortality factor 4-like protein 2 (Fragment) OS=Homo sapiens OX=9606 GN=MORF4L2 PE=1 SV=1 - [Q5JXX6_HUMAN] | 0 | 8.96 | 1.10771971889867 | 0.854616365179194 | 1.29616020009915 | 0.374244040448829 | 0.0429893077765466 | 1.36663954793362 | up |
| H0YID3 | Serine dehydratase-like (Fragment) OS=Homo sapiens OX=9606 GN=SDSL PE=1 SV=1 - [H0YID3_HUMAN] | 0 | 6.67 | 0.875905186421656 | 1.62683852358429 | 0.538409420310409 | -0.893224442524171 | 0.0306431567840615 | 1.51366649676532 | down |
| B4DPT1 | cDNA FLJ51524, highly similar to Set1/Ash2 histone methyltransferase complex subunit ASH2 OS=Homo sapiens OX=9606 PE=2 SV=1 - [B4DPT1_HUMAN] | 0 | 2.04 | 1.06483862909552 | 0.620515124880844 | 1.7160558806685 | 0.779096532703836 | 0.0379835924968668 | 1.42040396198497 | up |
| Q9HDB2 | Uncharacterized protein CATX-3 (Fragment) OS=Homo sapiens OX=9606 GN=CATX-3 PE=2 SV=1 - [Q9HDB2_HUMAN] | 0 | 21.88 | 0.90813283622136 | 1.2532106502698 | 0.724645003635945 | -0.464653688618317 | 0.0154082133753651 | 1.81224771599953 | down |
| O15050 | TPR and ankyrin repeat-containing protein 1 OS=Homo sapiens OX=9606 GN=TRANK1 PE=2 SV=4 - [TRNK1_HUMAN] | 0 | 1.26 | 1.16989622480118 | 0.662261718707461 | 1.76651645679366 | 0.820907189378018 | 0.0311091778540681 | 1.50711146617061 | up |
| Q9P225 | Dynein axonemal heavy chain 2 OS=Homo sapiens OX=9606 GN=DNAH2 PE=1 SV=3 - [DYH2_HUMAN] | 0 | 1.2 | 1.0988446612974 | 0.734454040826709 | 1.49613808382146 | 0.581243332746361 | 0.0315558650689777 | 1.50091990962536 | up |
| Q05DQ6 | Kinetochore protein NDC80 (Fragment) OS=Homo sapiens OX=9606 GN=NDC80 PE=2 SV=1 - [Q05DQ6_HUMAN] | 0 | 2.1 | 0.780240777752302 | 1.65724835131006 | 0.47080498051817 | -1.08679851255081 | 0.0135512975704992 | 1.86801911802004 | down |
| D3DVL8 | Adenylate cyclase OS=Homo sapiens OX=9606 GN=ADCY1 PE=3 SV=1 - [D3DVL8_HUMAN] | 0 | 2.35 | 1.10765540385067 | 0.801015434929792 | 1.38281405769384 | 0.467607175231684 | 0.00273092286691819 | 2.56369056604294 | up |
| O75663 | TIP41-like protein OS=Homo sapiens OX=9606 GN=TIPRL PE=1 SV=2 - [TIPRL_HUMAN] | 0 | 2.21 | 0.920401094360936 | 1.2129277247224 | 0.758826000594215 | -0.398158982445717 | 0.0281229339721225 | 1.55093937279042 | down |
| H6UKR8 | TOX2 variant 5 OS=Homo sapiens OX=9606 PE=2 SV=1 - [H6UKR8_HUMAN] | 0 | 2 | 1.03098439083617 | 0.672706749168053 | 1.53259112103633 | 0.615972852682226 | 0.0475459322962207 | 1.32288663239369 | up |
| B9ZVT1 | RNA-binding protein 12B OS=Homo sapiens OX=9606 GN=RBM12B PE=1 SV=2 - [B9ZVT1_HUMAN] | 0 | 1.93 | 1.02518499018199 | 0.791426039335774 | 1.29536424027999 | 0.373357822821811 | 0.00743197407770434 | 2.1288958138058 | up |
| P53803 | DNA-directed RNA polymerases I, II, and III subunit RPABC4 OS=Homo sapiens OX=9606 GN=POLR2K PE=1 SV=1 - [RPAB4_HUMAN] | 0 | 8.62 | 1.13558222784441 | 0.758834866896681 | 1.49648135237706 | 0.581574301555186 | 0.0444022367671822 | 1.35259515170573 | up |
| Q8TE58 | A disintegrin and metalloproteinase with thrombospondin motifs 15 OS=Homo sapiens OX=9606 GN=ADAMTS15 PE=2 SV=1 - [ATS15_HUMAN] | 0 | 1.16 | 1.11593394696081 | 0.869497434238203 | 1.28342408271568 | 0.359997959934469 | 0.0170687975681984 | 1.76779707220042 | up |
| Q5T9G4 | Armadillo repeat-containing protein 12 OS=Homo sapiens OX=9606 GN=ARMC12 PE=1 SV=1 - [ARM12_HUMAN] | 0 | 1.47 | 1.08661909460657 | 0.873468689100206 | 1.24402752859515 | 0.315018410652015 | 0.00908352822258936 | 2.04174543011575 | up |
| Q6ZWB5 | C3orf57 protein OS=Homo sapiens OX=9606 GN=C3orf57 PE=2 SV=1 - [Q6ZWB5_HUMAN] | 0 | 2.17 | 1.09311388366491 | 0.858026153015623 | 1.27398667257758 | 0.349350185359671 | 0.0477503035417709 | 1.32102386332427 | up |
| Q9C0C9 | (E3-independent) E2 ubiquitin-conjugating enzyme OS=Homo sapiens OX=9606 GN=UBE2O PE=1 SV=3 - [UBE2O_HUMAN] | 0 | 1.39 | 0.945117043070736 | 1.20762772012175 | 0.782622845867971 | -0.353610869724872 | 0.00517891034812775 | 2.28576160696828 | down |
| B8ZZT6 | Lysyl oxidase homolog OS=Homo sapiens OX=9606 GN=LOXL3 PE=1 SV=1 - [B8ZZT6_HUMAN] | 0 | 2.12 | 1.04259024634898 | 0.857396156627511 | 1.21599594107106 | 0.282138413151215 | 0.00915318598593726 | 2.03842771300714 | up |
| B3KUZ9 | Non-specific serine/threonine protein kinase OS=Homo sapiens OX=9606 PE=2 SV=1 - [B3KUZ9_HUMAN] | 0 | 1.58 | 1.02697824576319 | 0.792651225281817 | 1.2956243717381 | 0.37364751175684 | 0.0306483074373787 | 1.51359350453557 | up |
| A0A0S2Z3L7 | Beta-N-acetylhexosaminidase (Fragment) OS=Homo sapiens OX=9606 GN=HEXA PE=2 SV=1 - [A0A0S2Z3L7_HUMAN] | 0 | 5.8 | 0.888458812219349 | 1.29372333796637 | 0.686745601741974 | -0.54215232928825 | 0.0229926494663618 | 1.63841098165272 | down |
| Q8N118 | Cytochrome P450 4X1 OS=Homo sapiens OX=9606 GN=CYP4X1 PE=1 SV=1 - [CP4X1_HUMAN] | 0 | 1.18 | 1.12096874315338 | 0.844840859411985 | 1.32684011511184 | 0.407994535717648 | 0.00260326030491657 | 2.58448240379368 | up |
| B7Z7H3 | cDNA FLJ57654, highly similar to Zinc finger protein 307 OS=Homo sapiens OX=9606 PE=2 SV=1 - [B7Z7H3_HUMAN] | 0 | 2.82 | 1.20156933104018 | 0.349220372825301 | 3.44071945550918 | 1.78271026445334 | 0.0118916020980291 | 1.92475963104475 | up |
| B7ZVY5 | SLFN13 protein OS=Homo sapiens OX=9606 GN=SLFN13 PE=2 SV=1 - [B7ZVY5_HUMAN] | 0 | 0.78 | 1.1873152465183 | 0.65282623079081 | 1.8187309126351 | 0.862932107187281 | 0.0123890329486345 | 1.90696259204708 | up |
| A0A126LAV1 | U7 OS=Homo sapiens OX=9606 GN=U7 PE=4 SV=1 - [A0A126LAV1_HUMAN] | 0 | 1.63 | 1.00639948707345 | 0.786936218020096 | 1.27888317252129 | 0.354884478353722 | 0.00943375824192596 | 2.02531525757334 | up |
| Q3T7B8 | tRNA isopentenylpyrophosphate transferase isoform 5 OS=Homo sapiens OX=9606 GN=TRIT1 PE=2 SV=1 - [Q3T7B8_HUMAN] | 0 | 6.33 | 1.12898930376701 | 0.665127740104438 | 1.69740222169916 | 0.763328470886717 | 0.0480014606015276 | 1.31874554759248 | up |
| A0A087WW33 | Signal transducer CD24 (Fragment) OS=Homo sapiens OX=9606 GN=CD24 PE=1 SV=1 - [A0A087WW33_HUMAN] | 0 | 6.56 | 0.953141389630189 | 1.17800166089269 | 0.809117186564831 | -0.305579427791661 | 0.00162104555843142 | 2.79020477941593 | down |
| H0YHS3 | Synembryn-B (Fragment) OS=Homo sapiens OX=9606 GN=RIC8B PE=1 SV=1 - [H0YHS3_HUMAN] | 0 | 4.71 | 0.837786665575383 | 1.69370058149309 | 0.494648626049846 | -1.0155240251702 | 0.047372023513609 | 1.32447806382545 | down |
| Q6ZMF6 | Glycerol-3-phosphate acyltransferase 1, mitochondrial OS=Homo sapiens OX=9606 PE=2 SV=1 - [Q6ZMF6_HUMAN] | 0 | 1.57 | 0.984949301581835 | 1.20129528482819 | 0.819906074735573 | -0.286469445247373 | 0.00246604308167994 | 2.60799934056574 | down |
| O14598 | Testis-specific basic protein Y 1 OS=Homo sapiens OX=9606 GN=VCY PE=1 SV=1 - [VCY1_HUMAN] | 0 | 6.4 | 1.06569852200747 | 0.63614418631176 | 1.67524681501246 | 0.744373664264476 | 0.0127760258819174 | 1.89360421706662 | up |
| H7BXK5 | Bromodomain adjacent to zinc finger domain protein 2B (Fragment) OS=Homo sapiens OX=9606 GN=BAZ2B PE=1 SV=1 - [H7BXK5_HUMAN] | 0 | 8.06 | 1.07673319805252 | 0.676032933935154 | 1.59272299322004 | 0.671495374799098 | 0.0125147608780183 | 1.90257744412602 | up |
| A0A0A0MTG1 | Valine--tRNA ligase OS=Homo sapiens OX=9606 GN=VARS2 PE=1 SV=1 - [A0A0A0MTG1_HUMAN] | 0 | 3.68 | 1.03857236432155 | 0.67381392053904 | 1.54133408744466 | 0.624179602968912 | 0.0382512968880488 | 1.41735383575807 | up |
| F8W1D0 | Twinfilin-1 OS=Homo sapiens OX=9606 GN=TWF1 PE=4 SV=1 - [F8W1D0_HUMAN] | 0 | 4.2 | 1.08432148546802 | 0.818915379502998 | 1.32409466546604 | 0.405006270571237 | 0.0481098789567019 | 1.31776573577053 | up |
| Q9UBV2 | Protein sel-1 homolog 1 OS=Homo sapiens OX=9606 GN=SEL1L PE=1 SV=3 - [SE1L1_HUMAN] | 0 | 1.64 | 1.05373602886184 | 0.831526115550457 | 1.26723143044555 | 0.341680023347077 | 0.037938813897308 | 1.42091625083023 | up |
| Q6P0N0 | Mis18-binding protein 1 OS=Homo sapiens OX=9606 GN=MIS18BP1 PE=1 SV=1 - [M18BP_HUMAN] | 0 | 1.5 | 0.836617124773436 | 1.7570848089397 | 0.476139296473849 | -1.07054439334364 | 0.0436172595440326 | 1.36034162445866 | down |
| B4DQ93 | cDNA FLJ52996, highly similar to Syntenin-1 OS=Homo sapiens OX=9606 PE=2 SV=1 - [B4DQ93_HUMAN] | 0 | 11.65 | 1.15301377651064 | 0.702324929186395 | 1.64170988184392 | 0.715199200802983 | 0.000913787499386594 | 3.03915478738839 | up |
| K7EJT1 | Ubinuclein-1 (Fragment) OS=Homo sapiens OX=9606 GN=UBN1 PE=1 SV=8 - [K7EJT1_HUMAN] | 0 | 5.45 | 0.907461374003087 | 1.39084191736141 | 0.652454720177435 | -0.616050311583637 | 0.0309069242069046 | 1.50994421287376 | down |
| A0A0A0MQV6 | Fibroblast growth factor OS=Homo sapiens OX=9606 GN=FGF2 PE=1 SV=1 - [A0A0A0MQV6_HUMAN] | 0 | 5.9 | 0.979113214561093 | 1.21892738913285 | 0.803258031028112 | -0.316064594955991 | 0.00507489002007394 | 2.29457336507831 | down |
| Q53XS6 | Antigen p97 (Melanoma associated) identified by monoclonal antibodies 133.2 and 96.5 OS=Homo sapiens OX=9606 GN=MFI2 PE=2 SV=1 - [Q53XS6_HUMAN] | 0 | 1.66 | 0.553460695824197 | 2.2913068740379 | 0.241548044958662 | -2.04961791895388 | 0.0189957504657791 | 1.72134354408302 | down |
| Q6UXT9 | Protein ABHD15 OS=Homo sapiens OX=9606 GN=ABHD15 PE=1 SV=2 - [ABH15_HUMAN] | 0 | 2.56 | 1.1551404384675 | 0.917422501787978 | 1.25911500559037 | 0.332410062578232 | 0.0170176545316255 | 1.76910029702261 | up |
| B7Z1K1 | cDNA FLJ60431, highly similar to Regulator of G-protein signaling 11 OS=Homo sapiens OX=9606 PE=2 SV=1 - [B7Z1K1_HUMAN] | 0 | 1.7 | 1.08632113793268 | 0.890935151389244 | 1.21930438622696 | 0.286058324221235 | 0.0182605252665234 | 1.73848673408148 | up |
| Q6P1S2 | Protein C3orf33 OS=Homo sapiens OX=9606 GN=C3orf33 PE=1 SV=2 - [CC033_HUMAN] | 0 | 5.44 | 0.959806128361006 | 1.20835225790916 | 0.794309872869177 | -0.332226159509959 | 0.0301229710328256 | 1.52110219583361 | down |
| A0A8I5KCP1 | Uncharacterized protein OS=Homo sapiens OX=9606 PE=4 SV=1 - [A0A8I5KCP1_HUMAN] | 0 | 2.93 | 1.11945658939802 | 0.772550614532485 | 1.44903980184583 | 0.535097223008685 | 0.0221749652082381 | 1.65413705288889 | up |
| A0A1W2PQW2 | Voltage-dependent T-type calcium channel subunit alpha-1H OS=Homo sapiens OX=9606 GN=CACNA1H PE=1 SV=1 - [A0A1W2PQW2_HUMAN] | 0 | 0.68 | 0.949308027703361 | 1.17376462009633 | 0.808772058255985 | -0.306194939525593 | 0.000609260387150275 | 3.21519705788621 | down |
| Q8IX03 | Protein KIBRA OS=Homo sapiens OX=9606 GN=WWC1 PE=1 SV=1 - [KIBRA_HUMAN] | 0 | 1.98 | 0.949780184264088 | 1.203115129714 | 0.789434162040559 | -0.341109142998511 | 0.00615774774705313 | 2.21057810599903 | down |
| Q96FY7 | ACSS2 protein (Fragment) OS=Homo sapiens OX=9606 GN=ACSS2 PE=2 SV=2 - [Q96FY7_HUMAN] | 0 | 3.37 | 1.09404976853143 | 0.828829342455194 | 1.31999401142169 | 0.400531384347491 | 0.0217031350541265 | 1.66347752705632 | up |
| P0C7P0 | CDGSH iron-sulfur domain-containing protein 3, mitochondrial OS=Homo sapiens OX=9606 GN=CISD3 PE=1 SV=1 - [CISD3_HUMAN] | 0 | 4.72 | 0.895670975564518 | 1.3463389714565 | 0.665264093629825 | -0.588000925799987 | 0.00605836453012517 | 2.21764459883761 | down |
| H0YM74 | Zinc finger protein 592 OS=Homo sapiens OX=9606 GN=ZNF592 PE=1 SV=1 - [H0YM74_HUMAN] | 0 | 2.91 | 1.05785458836129 | 0.814401539889855 | 1.29893490685732 | 0.377329135245282 | 0.0481854717913676 | 1.31708388441519 | up |
| P08572 | Collagen alpha-2(IV) chain OS=Homo sapiens OX=9606 GN=COL4A2 PE=1 SV=4 - [CO4A2_HUMAN] | 0 | 1.52 | 1.11464779441857 | 0.826064298227083 | 1.3493474985069 | 0.432261934507505 | 0.0227486689664925 | 1.64304400900329 | up |
| Q9BS48 | HSF2 protein OS=Homo sapiens OX=9606 GN=HSF2 PE=2 SV=1 - [Q9BS48_HUMAN] | 0 | 4.78 | 1.1226578238559 | 0.704346789070568 | 1.59389925712207 | 0.672560446239638 | 0.0222483511067121 | 1.65270217038156 | up |
| Q9ULB4 | Cadherin-9 OS=Homo sapiens OX=9606 GN=CDH9 PE=2 SV=2 - [CADH9_HUMAN] | 0 | 1.27 | 0.84226715457609 | 1.49752364842078 | 0.562439969121228 | -0.830228973442683 | 0.0415618723011445 | 1.38130489634729 | down |
| Q15483 | S-laminin (Fragment) OS=Homo sapiens OX=9606 PE=2 SV=1 - [Q15483_HUMAN] | 0 | 0.81 | 0.971601036459376 | 1.17478583749339 | 0.827045241311773 | -0.273961844534451 | 0.0134657011160339 | 1.87077102933768 | down |
| B4DT66 | Protein SDA1 OS=Homo sapiens OX=9606 PE=2 SV=1 - [B4DT66_HUMAN] | 0 | 3.23 | 0.996171383716838 | 0.823890450614639 | 1.20910660267233 | 0.273941447537179 | 0.034852863970914 | 1.45776152873788 | up |
| Q96SB3 | Neurabin-2 OS=Homo sapiens OX=9606 GN=PPP1R9B PE=1 SV=3 - [NEB2_HUMAN] | 0 | 2.45 | 1.08538350628385 | 0.756794786122773 | 1.43418470394664 | 0.520230835970169 | 0.00476229805010558 | 2.322183427607 | up |
| B4DIK5 | cDNA FLJ57991, highly similar to Caspase recruitment domain-containing protein9 OS=Homo sapiens OX=9606 PE=2 SV=1 - [B4DIK5_HUMAN] | 0 | 4.09 | 0.786612612211397 | 1.65648014204231 | 0.474869931879512 | -1.07439568529385 | 0.0156136320594263 | 1.80649605914858 | down |
| H0Y580 | Translation initiation factor eIF-2B subunit gamma (Fragment) OS=Homo sapiens OX=9606 GN=EIF2B3 PE=1 SV=1 - [H0Y580_HUMAN] | 0 | 2.7 | 0.924825589995164 | 1.21478416362429 | 0.761308566318451 | -0.393446783248179 | 0.00678676959056398 | 2.16833689476687 | down |
| Q8NEG0 | Golgi associated RAB2 interactor protein 6 OS=Homo sapiens OX=9606 GN=GARIN6 PE=1 SV=1 - [GAR6_HUMAN] | 0 | 6.22 | 1.13483944214236 | 0.866500594199952 | 1.3096810893594 | 0.389215554654503 | 0.0371738793367049 | 1.42976211507827 | up |
| Q6P096 | MYBPC1 protein (Fragment) OS=Homo sapiens OX=9606 GN=MYBPC1 PE=2 SV=1 - [Q6P096_HUMAN] | 0 | 6.52 | 1.14118796984023 | 0.769731555743624 | 1.48257916844496 | 0.568109145495308 | 0.0228764300292819 | 1.64061174821608 | up |
| P0C1H6 | Histone H2B type F-M OS=Homo sapiens OX=9606 GN=H2BW2 PE=1 SV=2 - [H2BFM_HUMAN] | 0 | 3.9 | 0.931334311820733 | 1.22612815595697 | 0.759573383333526 | -0.396738742856395 | 0.039876484988049 | 1.39928313064055 | down |
| D9ZB55 | Apolipoprotein E (Fragment) OS=Homo sapiens OX=9606 GN=APOE PE=3 SV=1 - [D9ZB55_HUMAN] | 0 | 8.79 | 1.06428924483872 | 0.872148854828002 | 1.22030687645473 | 0.287243994892355 | 0.044127353245495 | 1.35529212071389 | up |
| E5RK48 | NADP-retinol dehydrogenase OS=Homo sapiens OX=9606 GN=RDH10 PE=1 SV=1 - [E5RK48_HUMAN] | 0 | 3.41 | 1.10025679414643 | 0.72250292884941 | 1.52284060065832 | 0.606764939412323 | 0.00987808104984983 | 2.00532741476506 | up |
| Q8N1X9 | cDNA FLJ37295 fis, clone BRAMY2015311, moderately similar to TRYPTOPHAN 5-MONOOXYGENASE OS=Homo sapiens OX=9606 PE=2 SV=1 - [Q8N1X9_HUMAN] | 0 | 5.2 | 0.936373926821602 | 1.33030452359487 | 0.703879382662884 | -0.506599866227692 | 0.0170785344142675 | 1.76754940080778 | down |
| Q70YC5 | Protein ZNF365 OS=Homo sapiens OX=9606 GN=ZNF365 PE=1 SV=3 - [ZN365_HUMAN] | 0 | 1.97 | 1.01983396956158 | 0.845578444032992 | 1.20607848598584 | 0.270323794185089 | 0.034703739539503 | 1.45962372480951 | up |
| F8WCI5 | Protein C3orf33 OS=Homo sapiens OX=9606 GN=C3orf33 PE=1 SV=1 - [F8WCI5_HUMAN] | 0 | 5.04 | 0.942311002644268 | 1.15736103835897 | 0.814189325035839 | -0.296563788686787 | 0.0370707251644146 | 1.43096891837229 | down |
| J3QR60 | Cadherin-3 (Fragment) OS=Homo sapiens OX=9606 GN=CDH3 PE=1 SV=1 - [J3QR60_HUMAN] | 0 | 10.17 | 1.06528941971558 | 0.763690487593489 | 1.39492299173776 | 0.480185478681943 | 0.0484782692408013 | 1.31445289363936 | up |
| H0Y933 | Ankyrin-2 (Fragment) OS=Homo sapiens OX=9606 GN=ANK2 PE=1 SV=1 - [H0Y933_HUMAN] | 0 | 5.58 | 1.10532692913085 | 0.614670624384469 | 1.79824264456712 | 0.846587702996369 | 0.0211146566739152 | 1.67541597584629 | up |
| A0A075B6W6 | T cell receptor alpha joining 27 (Fragment) OS=Homo sapiens OX=9606 GN=TRAJ27 PE=4 SV=1 - [A0A075B6W6_HUMAN] | 0 | 35 | 0.706088898698444 | 1.61215211336725 | 0.437979079544585 | -1.19106613501091 | 0.0496644367194801 | 1.30395448581409 | down |
| Q5TC84 | Opioid growth factor receptor-like protein 1 OS=Homo sapiens OX=9606 GN=OGFRL1 PE=2 SV=1 - [OGRL1_HUMAN] | 0 | 2.22 | 0.905965335995779 | 1.42104257782975 | 0.637535672843381 | -0.649422025709013 | 0.0165953175358139 | 1.78001443335888 | down |
| Q969S3 | Zinc finger protein 622 OS=Homo sapiens OX=9606 GN=ZNF622 PE=1 SV=1 - [ZN622_HUMAN] | 0 | 2.31 | 0.960775726003454 | 1.2011054863219 | 0.799909530798666 | -0.3220912534482 | 0.00120304200627285 | 2.91971920825949 | down |
| B4DV98 | cDNA FLJ53522, highly similar to Homo sapiens mucin and cadherin-like (MUCDHL), transcript variant 3, mRNA OS=Homo sapiens OX=9606 PE=2 SV=1 - [B4DV98_HUMAN] | 0 | 0.78 | 0.989280401075506 | 1.34283812961765 | 0.736708601920016 | -0.440834007040945 | 0.0368942901476834 | 1.43304084113716 | down |
| Q5T8T6 | Mediator of RNA polymerase II transcription subunit 22 (Fragment) OS=Homo sapiens OX=9606 GN=MED22 PE=4 SV=1 - [Q5T8T6_HUMAN] | 0 | 9.84 | 0.895401500267754 | 1.3025246939706 | 0.687435335708086 | -0.540704083531254 | 0.0354712606991512 | 1.450123375809 | down |
| H7C2P2 | Protein sidekick-2 (Fragment) OS=Homo sapiens OX=9606 GN=SDK2 PE=1 SV=1 - [H7C2P2_HUMAN] | 0 | 0.45 | 0.885737483177563 | 1.46785540117782 | 0.603422845647355 | -0.728758776794832 | 0.00670256080370575 | 2.17375923753246 | down |
| Q9P1H3 | HCG1773879 OS=Homo sapiens OX=9606 GN=hCG_1773879 PE=2 SV=1 - [Q9P1H3_HUMAN] | 0 | 9.72 | 0.938981143820636 | 1.19170689118128 | 0.787929608168893 | -0.343861346527818 | 0.00739957999712612 | 2.1307929302835 | down |
| Q0P5W4 | Farnesylated proteins-converting enzyme 2 (Fragment) OS=Homo sapiens OX=9606 GN=RCE1 PE=2 SV=1 - [Q0P5W4_HUMAN] | 0 | 4.86 | 1.23671536101992 | 0.544611322536667 | 2.27082197861697 | 1.1832146102133 | 0.00770824044177435 | 2.1130447469347 | up |
| A0A024RDD6 | GRIN_C domain-containing protein OS=Homo sapiens OX=9606 GN=LOC285513 PE=4 SV=1 - [A0A024RDD6_HUMAN] | 0 | 1.03 | 1.1256476755287 | 0.699590476576899 | 1.60900943225599 | 0.686172783382094 | 0.0237106639559242 | 1.6250562845559 | up |
| O76076 | CCN family member 5 OS=Homo sapiens OX=9606 GN=CCN5 PE=1 SV=1 - [CCN5_HUMAN] | 0 | 2.8 | 1.17123305462382 | 0.631464747237643 | 1.85478771340349 | 0.891254075069329 | 0.00216694279352225 | 2.66415255374043 | up |
| B5MCL8 | Phosphatidylinositol 4,5-bisphosphate 5-phosphatase A OS=Homo sapiens OX=9606 GN=INPP5J PE=1 SV=1 - [B5MCL8_HUMAN] | 0 | 2.61 | 0.766983701773339 | 1.73669200377385 | 0.441634843775796 | -1.17907409360585 | 0.0148591179900598 | 1.82800696873281 | down |
| B2R8F3 | cDNA, FLJ93871, highly similar to Homo sapiens melanoma antigen, family B, 2 (MAGEB2), mRNA OS=Homo sapiens OX=9606 PE=2 SV=1 - [B2R8F3_HUMAN] | 0 | 2.51 | 1.09299246214826 | 0.833179846141282 | 1.31183257397578 | 0.391583604019955 | 0.0350669369722513 | 1.45510216722785 | up |
| G3V114 | Docking protein 2 OS=Homo sapiens OX=9606 GN=DOK2 PE=1 SV=1 - [G3V114_HUMAN] | 0 | 4.88 | 0.849657202106704 | 1.59613801177186 | 0.532320636336145 | -0.909632598955111 | 0.0395122892460665 | 1.4032678076222 | down |
| A0A087WYA0 | Fatty-acid amide hydrolase 1 (Fragment) OS=Homo sapiens OX=9606 GN=FAAH PE=1 SV=1 - [A0A087WYA0_HUMAN] | 0 | 12.24 | 1.12672586987414 | 0.903088635001685 | 1.24763597525733 | 0.319197058158994 | 0.0348177048230704 | 1.45819986089039 | up |
| B4DXI9 | cDNA FLJ55940, highly similar to Transcription intermediary factor 1-gamma OS=Homo sapiens OX=9606 PE=2 SV=1 - [B4DXI9_HUMAN] | 0 | 1.74 | 1.01271331206232 | 0.710350999011005 | 1.4256519853879 | 0.511621849782455 | 0.00486839108822649 | 2.31261454123975 | up |
| Q8N8A2 | Serine/threonine-protein phosphatase 6 regulatory ankyrin repeat subunit B OS=Homo sapiens OX=9606 GN=ANKRD44 PE=1 SV=3 - [ANR44_HUMAN] | 0 | 1.41 | 1.10916103813248 | 0.763192317238683 | 1.45331787686956 | 0.539350290875854 | 0.000702688476713702 | 3.15323716833653 | up |
| Q9HCG4 | KIAA1608 protein (Fragment) OS=Homo sapiens OX=9606 GN=KIAA1608 PE=2 SV=2 - [Q9HCG4_HUMAN] | 0 | 0.69 | 1.07024385615441 | 0.563439949999696 | 1.89948166819726 | 0.925605788692318 | 0.0474226314080443 | 1.32401435136402 | up |
| Q4G0A6 | Probable ubiquitin carboxyl-terminal hydrolase MINDY-4 OS=Homo sapiens OX=9606 GN=MINDY4 PE=1 SV=2 - [MINY4_HUMAN] | 0 | 1.72 | 1.06200856785545 | 0.87549337010604 | 1.21304010300709 | 0.278627246627487 | 0.00873290451423958 | 2.05884128840051 | up |
| Q13257 | Mitotic spindle assembly checkpoint protein MAD2A OS=Homo sapiens OX=9606 GN=MAD2L1 PE=1 SV=1 - [MD2L1_HUMAN] | 0 | 3.41 | 0.928531697354682 | 1.21380935832151 | 0.764973256293465 | -0.386518783274521 | 0.0213478621914321 | 1.67064560939542 | down |
| Q86SJ6 | Desmoglein-4 OS=Homo sapiens OX=9606 GN=DSG4 PE=1 SV=1 - [DSG4_HUMAN] | 0 | 1.54 | 0.958502484632754 | 1.33187578123329 | 0.719663573839597 | -0.474605457475416 | 0.0312769187414441 | 1.50477603820494 | down |
| G5E9N5 | ALS2 C-terminal like, isoform CRA_a OS=Homo sapiens OX=9606 GN=ALS2CL PE=1 SV=1 - [G5E9N5_HUMAN] | 0 | 2.06 | 1.07320110036526 | 0.740749299908316 | 1.44880474473361 | 0.534863176133211 | 0.04804593718803 | 1.31834333081374 | up |
| Q53EP8 | Interleukin-4 receptor subunit alpha (Fragment) OS=Homo sapiens OX=9606 PE=2 SV=1 - [Q53EP8_HUMAN] | 0 | 0.73 | 1.10717829225614 | 0.815853395692596 | 1.35707995836216 | 0.440505726014072 | 0.0376921018305843 | 1.42374964424386 | up |
| Q9Y5G5 | Protocadherin gamma-A8 OS=Homo sapiens OX=9606 GN=PCDHGA8 PE=2 SV=1 - [PCDG8_HUMAN] | 0 | 0.97 | 0.762352279904677 | 1.68602849221231 | 0.452158598402073 | -1.14509919615151 | 0.0156049099428504 | 1.80673873333867 | down |
| Q8N2Z9 | Centromere protein S OS=Homo sapiens OX=9606 GN=CENPS PE=1 SV=1 - [CENPS_HUMAN] | 0 | 4.35 | 1.09954196574754 | 0.742977477734822 | 1.4799129162028 | 0.565512284598333 | 0.00856330089214357 | 2.06735879573043 | up |
| B4DEN5 | Beta-1,4-glucuronyltransferase 1 OS=Homo sapiens OX=9606 PE=2 SV=1 - [B4DEN5_HUMAN] | 0 | 2.21 | 1.05949866350811 | 0.646846189089573 | 1.63794528185957 | 0.711887162285142 | 0.0240199955234997 | 1.61942707787065 | up |
| B0QYW5 | Peroxisomal membrane protein PMP34 OS=Homo sapiens OX=9606 GN=SLC25A17 PE=1 SV=1 - [B0QYW5_HUMAN] | 0 | 8 | 0.999490892890545 | 0.756250610282341 | 1.32163978355983 | 0.402329020048432 | 0.0320597891175069 | 1.49403933866947 | up |
| E9PQP6 | Protein farnesyltransferase/geranylgeranyltransferase type-1 subunit alpha (Fragment) OS=Homo sapiens OX=9606 GN=FNTA PE=1 SV=1 - [E9PQP6_HUMAN] | 0 | 4.02 | 0.952223394696075 | 1.20759391464963 | 0.788529474307888 | -0.342763412613372 | 0.0374738967889646 | 1.42627114368362 | down |
| Q69YU5 | Protein BRAWNIN OS=Homo sapiens OX=9606 GN=BRAWNIN PE=1 SV=2 - [BWNIN_HUMAN] | 0 | 9.86 | 1.05545895547523 | 0.665930927684141 | 1.58493758376071 | 0.664426026880807 | 0.0487206525722924 | 1.31228690332576 | up |
| A0A8I5F967 | Sphingosine 1-phosphate receptor 3 OS=Homo sapiens OX=9606 GN=S1PR3 PE=4 SV=1 - [A0A8I5F967_HUMAN] | 0 | 2.48 | 1.03040255195627 | 0.724889352512044 | 1.42146183881098 | 0.507375368310437 | 0.042753695126625 | 1.36902634400593 | up |
| Q96T98 | Protocadherin-psi1 OS=Homo sapiens OX=9606 GN=PCDH-psi1 PE=2 SV=1 - [Q96T98_HUMAN] | 0 | 1.02 | 0.636814249424206 | 2.34516797882991 | 0.271543128327181 | -1.88074674001684 | 0.0472019638672995 | 1.32603993189582 | down |
| H0Y7G9 | Serine protease HTRA1 (Fragment) OS=Homo sapiens OX=9606 GN=HTRA1 PE=1 SV=1 - [H0Y7G9_HUMAN] | 0 | 4.98 | 1.09579725008786 | 0.739971871174137 | 1.48086338518398 | 0.566438553112522 | 0.0365374057954189 | 1.43726229137676 | up |
| Q2V4X9 | Alpha-1 collagen type II (Fragment) OS=Homo sapiens OX=9606 GN=COL2A1 PE=4 SV=1 - [Q2V4X9_HUMAN] | 0 | 2.08 | 1.05307140752213 | 0.570685554738032 | 1.84527433501543 | 0.883835316201449 | 0.0425679999700392 | 1.37091675438901 | up |
| K7VKE0 | Krueppel-like factor 1 OS=Homo sapiens OX=9606 GN=KLF1 PE=4 SV=1 - [K7VKE0_HUMAN] | 0 | 4.25 | 1.14722951638485 | 0.652464031120043 | 1.75830308134454 | 0.814183771428718 | 0.0372882857473786 | 1.42842758197972 | up |
| Q8N9Z6 | cDNA FLJ36019 fis, clone TESTI2016421, highly similar to PROTEIN KINASE C-BINDING PROTEIN NELL1 OS=Homo sapiens OX=9606 PE=2 SV=1 - [Q8N9Z6_HUMAN] | 0 | 1.97 | 0.872829721370247 | 1.28150265513387 | 0.681098644527636 | -0.554064333853302 | 0.0490299658551328 | 1.30953840919993 | down |
| E7EMT6 | TRAF family member-associated NF-kappa-B activator (Fragment) OS=Homo sapiens OX=9606 GN=TANK PE=1 SV=1 - [E7EMT6_HUMAN] | 0 | 11.52 | 1.16936778947414 | 0.624117961479073 | 1.87363264903144 | 0.905838120888443 | 0.00954528809006151 | 2.02021095946142 | up |
| B7ZMN0 | LINGO2 protein OS=Homo sapiens OX=9606 GN=LINGO2 PE=2 SV=1 - [B7ZMN0_HUMAN] | 0 | 1.65 | 1.11519775879739 | 0.776471565738945 | 1.43623772975652 | 0.52229456751366 | 0.0159729722014725 | 1.79661426416232 | up |
| F8VNU2 | Rabphilin-3A (Fragment) OS=Homo sapiens OX=9606 GN=RPH3A PE=1 SV=1 - [F8VNU2_HUMAN] | 0 | 16.87 | 1.12515437318853 | 0.590549079320475 | 1.9052681861484 | 0.929994086219495 | 0.0217732660473653 | 1.66207642075407 | up |
| Q6UWE9 | SFVP2550 OS=Homo sapiens OX=9606 GN=UNQ2550 PE=2 SV=1 - [Q6UWE9_HUMAN] | 0 | 7.77 | 1.03528223164702 | 0.768091886906087 | 1.34786247491454 | 0.430673302988341 | 0.0148257577568159 | 1.82898310024406 | up |
| B4DHR3 | cDNA FLJ57725 OS=Homo sapiens OX=9606 PE=2 SV=1 - [B4DHR3_HUMAN] | 0 | 4.35 | 0.909920982941476 | 1.40252821971557 | 0.648771960628364 | -0.624216626035863 | 0.0264044404511026 | 1.57832303141629 | down |
| B0QY71 | Transcription factor MafF (Fragment) OS=Homo sapiens OX=9606 GN=MAFF PE=1 SV=1 - [B0QY71_HUMAN] | 0 | 12.9 | 1.05918491711653 | 0.800744585025029 | 1.32275002157326 | 0.403540441274746 | 0.0445995976786632 | 1.35066905892742 | up |
| Q9UGQ3 | Solute carrier family 2, facilitated glucose transporter member 6 OS=Homo sapiens OX=9606 GN=SLC2A6 PE=1 SV=2 - [GTR6_HUMAN] | 0 | 1.97 | 1.04716550730381 | 0.503024783949735 | 2.08173740284027 | 1.05778809384692 | 0.0265684571107803 | 1.57563366530106 | up |
| A0A087WWN9 | Fatty acid desaturase 6 (Fragment) OS=Homo sapiens OX=9606 GN=FADS6 PE=1 SV=1 - [A0A087WWN9_HUMAN] | 0 | 5.69 | 1.05180667899049 | 0.814750783484816 | 1.29095509978124 | 0.368438823689706 | 0.0154382691635117 | 1.81140139149198 | up |
| H7C3C1 | Interleukin-1 receptor-associated kinase 1 (Fragment) OS=Homo sapiens OX=9606 GN=IRAK1 PE=1 SV=1 - [H7C3C1_HUMAN] | 0 | 2.75 | 1.09423552067377 | 0.809226415862814 | 1.35219945768462 | 0.43530797365301 | 0.0369099849587391 | 1.43285613178451 | up |
| F2Z3H2 | Phosphatidylserine lipase ABHD16A OS=Homo sapiens OX=9606 GN=ABHD16A PE=1 SV=1 - [F2Z3H2_HUMAN] | 0 | 15.38 | 1.15583878689312 | 0.628250887709509 | 1.83977262826815 | 0.879527479141459 | 0.020246763191304 | 1.69364439667228 | up |
| Q9BTY2 | Plasma alpha-L-fucosidase OS=Homo sapiens OX=9606 GN=FUCA2 PE=1 SV=2 - [FUCO2_HUMAN] | 0 | 1.5 | 1.06510316443931 | 0.8053906270507 | 1.32246779223104 | 0.40323258694847 | 0.0138812690214405 | 1.85757082899527 | up |
| K7EIL3 | Intercellular adhesion molecule 5 (Fragment) OS=Homo sapiens OX=9606 GN=ICAM5 PE=1 SV=8 - [K7EIL3_HUMAN] | 0 | 8.97 | 1.1124331656337 | 0.739925456568296 | 1.50343950969474 | 0.588266822804492 | 0.0329387681795858 | 1.48229264629595 | up |
| Q9BUV8 | Respirasome Complex Assembly Factor 1 OS=Homo sapiens OX=9606 GN=RAB5IF PE=1 SV=1 - [RCAF1_HUMAN] | 0 | 4.38 | 1.04797665654983 | 0.766181604518448 | 1.36779146141012 | 0.451848288320865 | 0.00919458803449955 | 2.03646772464794 | up |
| D6RBD9 | Thioredoxin domain-containing protein 15 (Fragment) OS=Homo sapiens OX=9606 GN=TXNDC15 PE=1 SV=1 - [D6RBD9_HUMAN] | 0 | 5.16 | 0.887034669236646 | 1.32900876622818 | 0.667440796311758 | -0.583288223114117 | 0.00351664509925288 | 2.45387145858477 | down |
| Q9UE56 | Proto-oncogene c-sis for PDGF B chain (platelet-derived growth factor) OS=Homo sapiens OX=9606 PE=4 SV=1 - [Q9UE56_HUMAN] | 0 | 17.5 | 0.924489992013662 | 1.28615926032734 | 0.718799001438105 | -0.476339690242755 | 0.000859313996039802 | 3.0658481145972 | down |
| Q5UBZ3 | SH2 domain containing adapter protein 2 transcript variant 3 OS=Homo sapiens OX=9606 GN=SH2D2A PE=2 SV=1 - [Q5UBZ3_HUMAN] | 0 | 1.66 | 0.95177252977183 | 1.14450489961213 | 0.831601970506532 | -0.266034918176609 | 0.0314549401390843 | 1.50231113697047 | down |
| X5D9N8 | Patched domain containing 1 isoform C OS=Homo sapiens OX=9606 GN=PTCHD1 PE=2 SV=1 - [X5D9N8_HUMAN] | 0 | 7.73 | 0.868397726974348 | 2.0492960529302 | 0.423754159743128 | -1.23870056448776 | 0.030662030545337 | 1.51339908805194 | down |
| M5AP16 | Receptor (G protein-coupled) activity modifying protein 3 (Fragment) OS=Homo sapiens OX=9606 GN=RAMP3 PE=4 SV=1 - [M5AP16_HUMAN] | 0 | 31.58 | 1.10797582060701 | 0.76071308365877 | 1.45649633798596 | 0.542502074168866 | 0.028644254834583 | 1.54296247121287 | up |
| A0A0U1RR08 | RNA polymerase II elongation factor ELL2 (Fragment) OS=Homo sapiens OX=9606 GN=ELL2 PE=1 SV=1 - [A0A0U1RR08_HUMAN] | 0 | 9.23 | 1.19359522239344 | 0.623884213625684 | 1.91316785442751 | 0.935963456081622 | 0.000435021411390621 | 3.36148936690754 | up |
| H7C0J7 | Eukaryotic translation initiation factor 4E transporter (Fragment) OS=Homo sapiens OX=9606 GN=EIF4ENIF1 PE=1 SV=1 - [H7C0J7_HUMAN] | 0 | 7.45 | 1.06495406202726 | 0.739434908169135 | 1.44022692229141 | 0.526296140745462 | 0.0230338317755883 | 1.63763380929718 | up |
| V9H0U8 | Prolactin (Fragment) OS=Homo sapiens OX=9606 PE=2 SV=1 - [V9H0U8_HUMAN] | 0 | 7.25 | 1.04971688818478 | 0.690152934912423 | 1.52099170355334 | 0.605012283716518 | 0.020186694766612 | 1.69493478368788 | up |
| Q86TX6 | Full-length cDNA clone CS0DI006YN07 of Placenta of Homo sapiens (human) OS=Homo sapiens OX=9606 PE=2 SV=1 - [Q86TX6_HUMAN] | 0 | 25.58 | 0.823147045896722 | 1.75231774761921 | 0.469747594016605 | -1.09004232251122 | 0.0181106135642966 | 1.74206683609817 | down |
| I3L1H3 | Lipopolysaccharide-induced tumor necrosis factor-alpha factor OS=Homo sapiens OX=9606 GN=LITAF PE=1 SV=1 - [I3L1H3_HUMAN] | 0 | 10.29 | 1.14890621748273 | 0.719360896633611 | 1.59712075379584 | 0.675473394976115 | 0.00745960068874782 | 2.12728441962047 | up |
| A1E4E8 | G protein-coupled receptor 144 (Fragment) OS=Homo sapiens OX=9606 GN=GPR144 PE=2 SV=1 - [A1E4E8_HUMAN] | 0 | 4.62 | 1.08457030321622 | 0.777476463320853 | 1.39498795704203 | 0.480252667294077 | 0.0140481095907914 | 1.85238211344749 | up |
| A0A2X0SF83 | ARHGAP21 (Fragment) OS=Homo sapiens OX=9606 GN=ARHGAP21 PE=4 SV=1 - [A0A2X0SF83_HUMAN] | 0 | 1.64 | 1.10301181167083 | 0.785314252265594 | 1.40454831742668 | 0.490106255018138 | 0.0170808581123505 | 1.76749031490596 | up |
| A0A2U3TZL8 | Kinesin-like protein OS=Homo sapiens OX=9606 GN=KIF23 PE=1 SV=1 - [A0A2U3TZL8_HUMAN] | 0 | 1.48 | 0.937629565387301 | 1.1579067973658 | 0.809762553877716 | -0.304429165362755 | 0.0245471635118576 | 1.60999868449085 | down |
| A0A3B3IUC3 | Probable G-protein-coupled receptor 158 OS=Homo sapiens OX=9606 GN=GPR158 PE=1 SV=1 - [A0A3B3IUC3_HUMAN] | 0 | 1.58 | 1.07747850137855 | 0.541450388511592 | 1.98998564640514 | 0.992758024771531 | 0.00709078831976369 | 2.14930547935974 | up |
| A0A494C0R9 | Lipopolysaccharide-responsive and beige-like anchor protein OS=Homo sapiens OX=9606 GN=LRBA PE=1 SV=1 - [A0A494C0R9_HUMAN] | 0 | 0.85 | 1.14605643371675 | 0.827498990879399 | 1.38496414660133 | 0.469848628844873 | 0.0368347115991691 | 1.43374272614518 | up |
| A0A384P5W4 | Epididymis secretory sperm binding protein OS=Homo sapiens OX=9606 PE=2 SV=1 - [A0A384P5W4_HUMAN] | 0 | 2.61 | 1.07229578140363 | 0.876635496515234 | 1.22319457250611 | 0.290653910375862 | 0.0358369653581912 | 1.4456687731168 | up |
| A0A5H1ZRS1 | Pericentriolar material 1 protein OS=Homo sapiens OX=9606 GN=PCM1 PE=1 SV=1 - [A0A5H1ZRS1_HUMAN] | 0 | 0.6 | 0.949817485750683 | 1.23893266678652 | 0.766641732205083 | -0.383375561389413 | 0.00101838080609602 | 2.99208979463046 | down |
| A0A590UKC9 | DNA mismatch repair protein Msh3 OS=Homo sapiens OX=9606 GN=MSH3 PE=1 SV=1 - [A0A590UKC9_HUMAN] | 0 | 2.22 | 0.777678861838518 | 1.64773066044893 | 0.471969649230559 | -1.0832340071486 | 0.00079035016979789 | 3.10218044906821 | down |
| E9PIH7 | PH domain-containing protein (Fragment) OS=Homo sapiens OX=9606 PE=4 SV=2 - [E9PIH7_HUMAN] | 0 | 5.28 | 0.956980874035434 | 1.18390280352273 | 0.808327230232008 | -0.306988646139645 | 0.0171544011716024 | 1.76562443775983 | down |
| A0A5C2G398 | IGL c3741_light_IGKV1-39_IGKJ4 (Fragment) OS=Homo sapiens OX=9606 PE=2 SV=1 - [A0A5C2G398_HUMAN] | 0 | 5.61 | 0.885751833988526 | 1.42103931815531 | 0.623312685773075 | -0.681972019848072 | 0.0129321410179453 | 1.88832956829018 | down |
| A0A5C2GDX8 | IG c669_heavy_IGHV3-21_IGHD5-18_IGHJ4 (Fragment) OS=Homo sapiens OX=9606 PE=2 SV=1 - [A0A5C2GDX8_HUMAN] | 0 | 4.92 | 1.10606283927684 | 0.759960967648288 | 1.45542058916469 | 0.541436125105675 | 0.0320996019648639 | 1.49350035281429 | up |
| A0A669KB46 | Sodium-dependent phosphate transport protein 2C (Fragment) OS=Homo sapiens OX=9606 GN=SLC34A3 PE=1 SV=1 - [A0A669KB46_HUMAN] | 0 | 4.86 | 0.813218789711367 | 1.57149960193995 | 0.517479475468834 | -0.950426452036824 | 0.0459898100710813 | 1.33733838397351 | down |
| A0A6I8PLD9 | Keratinocyte-associated protein 2 OS=Homo sapiens OX=9606 GN=KRTCAP2 PE=1 SV=1 - [A0A6I8PLD9_HUMAN] | 0 | 4.39 | 0.99805673869495 | 0.725984859214654 | 1.37476247063143 | 0.459182373514047 | 0.0338178597634043 | 1.47085388113672 | up |
| A0A6Q8PHS7 | Phosphatidylinositol-3,5-bisphosphate 3-phosphatase OS=Homo sapiens OX=9606 GN=MTMR2 PE=1 SV=1 - [A0A6Q8PHS7_HUMAN] | 0 | 3.61 | 0.895751563731338 | 1.29378162531442 | 0.692351434125253 | -0.530423566318025 | 0.00896427365425358 | 2.04748489413628 | down |
| A0A6Q8PGJ2 | Intraflagellar transport protein 172 homolog OS=Homo sapiens OX=9606 GN=IFT172 PE=1 SV=1 - [A0A6Q8PGJ2_HUMAN] | 0 | 0.69 | 1.04520256533985 | 0.867685833606219 | 1.20458641233757 | 0.268537890611854 | 0.0205673412680718 | 1.68682184574981 | up |
| A0A6Q8PHK8 | Ankyrin repeat domain-containing protein 26 (Fragment) OS=Homo sapiens OX=9606 GN=ANKRD26 PE=1 SV=1 - [A0A6Q8PHK8_HUMAN] | 0 | 3.49 | 0.971054290248205 | 1.19818089610559 | 0.810440471388248 | -0.303221874335149 | 0.0371054631371578 | 1.43056214333239 | down |
| A0A7P0T939 | A-kinase anchor protein 9 OS=Homo sapiens OX=9606 GN=AKAP9 PE=1 SV=1 - [A0A7P0T939_HUMAN] | 0 | 1.7 | 1.02910452096144 | 0.742466165031985 | 1.38606251628599 | 0.470992329554314 | 0.0436797738430113 | 1.35971961890649 | up |
| A0A7I2YQA6 | Golgi-specific brefeldin A-resistance guanine nucleotide exchange factor 1 (Fragment) OS=Homo sapiens OX=9606 GN=GBF1 PE=1 SV=1 - [A0A7I2YQA6_HUMAN] | 0 | 5 | 0.985242781611338 | 1.18736646524736 | 0.829771439945533 | -0.269214093206882 | 0.0424328831579351 | 1.37229745852229 | down |
| A0A7S5BZ99 | IGH c440_heavy_IGHV1-2_IGHD3-16_IGHJ6 (Fragment) OS=Homo sapiens OX=9606 PE=2 SV=1 - [A0A7S5BZ99_HUMAN] | 0 | 7.38 | 0.95157578855624 | 1.19896279450001 | 0.793665819257607 | -0.333396420499512 | 0.0487549752389157 | 1.31198105978991 | down |
| A0A8I5KUN1 | Protein dopey-2 OS=Homo sapiens OX=9606 GN=DOP1B PE=4 SV=1 - [A0A8I5KUN1_HUMAN] | 0 | 0.9 | 1.12544876511113 | 0.893334004986188 | 1.25982976001068 | 0.333228796438056 | 0.0229453759593313 | 1.63930482200831 | up |
| A0A8I5KXB5 | CDK5 regulatory subunit-associated protein 2 OS=Homo sapiens OX=9606 GN=CDK5RAP2 PE=4 SV=1 - [A0A8I5KXB5_HUMAN] | 0 | 1.34 | 0.970324473633031 | 1.2327521592001 | 0.787120481916369 | -0.345343613716658 | 0.0409118220113995 | 1.38815117873002 | down |
| A0A804HLD3 | Dynein axonemal heavy chain 14 OS=Homo sapiens OX=9606 GN=DNAH14 PE=1 SV=1 - [A0A804HLD3_HUMAN] | 0 | 0.87 | 1.05093186160733 | 0.853494793628255 | 1.23132779420922 | 0.300214875654797 | 0.0237929304201562 | 1.62355206544792 | up |
| A0A804HKG2 | Exostosin-2 OS=Homo sapiens OX=9606 GN=EXT2 PE=1 SV=1 - [A0A804HKG2_HUMAN] | 0 | 1.65 | 0.976237260467909 | 1.23175944663094 | 0.792555123598263 | -0.335416814176414 | 0.0383234078303515 | 1.41653587913128 | down |
| I3L306 | Kinesin-like protein KIF22 (Fragment) OS=Homo sapiens OX=9606 GN=KIF22 PE=1 SV=2 - [I3L306_HUMAN] | 0 | 6.85 | 1.07014157812232 | 0.794390402444919 | 1.34712299497667 | 0.429881577706035 | 0.0324454289704658 | 1.48884647953344 | up |
| C9JW07 | LisH domain-containing protein ARMC9 OS=Homo sapiens OX=9606 GN=ARMC9 PE=1 SV=2 - [C9JW07_HUMAN] | 0 | 6.9 | 1.08752299404737 | 0.587582418414568 | 1.85084331995801 | 0.888182771444627 | 0.0107718898770754 | 1.9677080951067 | up |
